# Supplementary material for: Marine biogeographic realms and species endemicity
Source: Nat Commun. 2017 Oct 20;8:1057. doi: 10.1038/s41467-017-01121-2 (PMC5648874; doi:10.1038/s41467-017-01121-2)
Supplement: Supplementary file 1 — Supplementary Information [file 41467_2017_1121_MOESM1_ESM.pdf]

Supplementary Information File

from the paper

Costello MJ, Tsai P, Wong PS, Cheung AKL, Basher Z, Chauhdary C. 2017. Marine biogeographic realms and species endemicity. *Nature Communications.*

Supplementary Table 1. Seas that had no significant difference in species and genus composition as determined by the SIMPROF test.

Species level analysis	Genus level analysis
Gulf of Aqaba + Gulf of Suez	Same
Molukka Sea + Halamahera Sea	Add Gulf of Boni
Makassar Strait + Gulf of Boni	Add Bali Sea + Flores Sea + Savu Sea + Java Sea. Excluded Gulf of Boni
Gulf of Riga + Gulf of Bothnia + Gulf of Finland	Excluded Gulf of Riga
Laptev Sea + East Siberian Sea	Same
Hudson Strait + Hudson Bay	Same
Baffin Bay + Northwestern Passages	Same
Labrador Sea + Gulf of St-Lawrence	Same
Gulf of Alaska + Bering Sea	Not grouped
Barentsz Sea + Greenland Sea	Same
Beaufort Sea + Chukchi Sea	Same
White Sea + Kara Sea	Same
Mediterranean Sea - Eastern Basin + Mediterranean Sea - Western Basin	Add Adriatic Sea but excluded + Mediterranean Sea -Western Basin
Tyrrhenian Sea + Ionian Sea	Same
Irish Sea and St. George's Channel + Inner Seas off the West Coast of Scotland	Same
Baltic Sea + Skaggerak	Add Bay of Fundy but excluded Skaggerak
Alboran Sea + Balearic Sea	Same
Japan Sea + Inland Sea	Add Yellow Sea and Eastern China Sea but excluded Inland Sea
Singapore Strait + Gulf of Thailand	Same
Flores Sea + Savu Sea	Not grouped
Great Australian Bight + Bass Strait	Same
Gulf of Mexico + Caribbean Sea	Same
South Atlantic Ocean + Southern Ocean	Add Indian Ocean but excluded Southern Ocean
Red Sea + Gulf of Aden	Same
Laccadive Sea + Andaman or Burma Sea	See below
Arabian Sea + Bay of Bengal	Add Laccadive Sea
Arafura Sea + Timor Sea	Same
Indian Ocean + Mozambique Channel	Not grouped
South Pacific Ocean + Coral Sea	Add North Pacific Ocean but excluded Coral Sea
Philippine Sea + Sulu Sea	Add South China Sea and Celebes Sea
Bismarck Sea + Solomon Sea	Same
Not grouped	The Coastal Waters of Southeast Alaska and British Columbia + Gulf of Alaska
Not grouped	Bay of Biscay + Gulf of Guinea

Supplementary Table 2. The 100 species that occurred in most of the 98 seas and oceans, and their total number of distribution records, as used in the present analysis.

Classification	Common name	*Species*	Seas present	Records
Chromista	Phytoplankton	*Thalassionema nitzschioides*	46	32686
Chromista	Phytoplankton	*Nitzschia seratia*	46	17507
Chromista	Phytoplankton	*Pseudo-nitzschia delicatissima*	44	17400
Chromista	Phytoplankton	*Rhizosolenia styliformis*	43	20499
Protozoa	Phytoplankton	*Ceratium fusus*	41	62367
Protozoa	Phytoplankton	*Ceratium furca*	39	40357
Protozoa	Phytoplankton	*Ceratium tripos*	38	33911
Chromista	Phytoplankton	*Thalassiothrix longissima*	38	20691
Copepoda	Planktonic copepod	*Metridia lucens*	36	42235
Copepoda	Planktonic copepod	*Oithona similis*	35	39811
Copepoda	Planktonic copepod	*Calanus finmarchicus finmarchicus*	34	55755
Protozoa	Phytoplankton	*Ceratium macroceros*	33	23764
Pisces	Atlantic herring	*Clupea harengus*	32	37668
Mammalia	Fin whale	*Balaenoptera physalus*	32	18622
Protozoa	Phytoplankton	*Ceratium horridum*	29	20263
Aves	Kittiwake	*Rissa tridactyla*	27	141466
Pisces	Spiny dogfish	*Squalus acanthias*	27	52985
Gastropoda	Planktonic snail	*Limacina retroversa*	27	40403
Aves	Herring gull	*Larus argentatus*	26	75818
Copepoda	Planktonic copepod	*Pseudocalanus minutus*	26	21580
Copepoda	Planktonic copepod	*Acartia (Acartiura) longiremis*	26	16205
Pisces	Atlantic cod	*Gadus morhua*	25	192341
Copepoda	Planktonic copepod	*Calanus helgolandicus*	25	44695
Copepoda	Planktonic copepod	*Metridia longa*	25	23046
Aves	Northern fulmar	*Fulmarus glacialis*	24	314000
Aves	Common guillemot	*Uria aalge*	24	194302
Copepoda	Planktonic copepod	*Temora longicornis*	24	42656
Copepoda	Planktonic copepod	*Centropages typicus*	22	34936
Copepoda	Planktonic copepod	*Centropages hamatus*	22	18210
Aves	Lesser black-backed gull	*Larus fuscus*	22	17556
Aves	Great black-backed gull	*Larus marinus*	21	44626
Aves	Sooty shearwater	*Puffinus griseus*	21	21788
Reptilia	Loggerhead turtle	*Caretta caretta*	20	58726
Aves	Atlantic puffin	*Fratercula arctica*	20	41829
Pisces	Poor cod	*Pollachius virens*	20	19226
Pisces	Witch flounder	*Glyptocephalus cynoglossus*	19	34377
Pisces	Haddock	*Melanogrammus aeglefinus*	19	31622
Mammalia	Harbour porpoise	*Phocoena phocoena*	19	18112
Pisces	American plaice	*Hippoglossoides platessoides*	18	57179
Aves	Razorbill	*Alca torda*	18	31670
Cephalopoda	Short-fin squid	*Illex illecebrosus*	18	23813
Pisces	Thorny skate	*Amblyraja radiata*	16	42530
Pisces	Greenland halibut	*Reinhardtius hippoglossoides*	16	23553
Aves	Storm petrel	*Oceanites oceanicus*	16	15654
Aves	Manx shearwater	*Puffinus puffinus*	15	34218
Pisces	Tope, school shark	*Galeorhinus galeus*	15	23360
Pisces	Yellowtail flounder	*Limanda ferruginea*	14	27871
Pisces	Longhorn sculpin	*Myoxocephalus octodecemspinosus*	14	27004
Pisces	Red hake	*Urophycis chuss*	14	26410
Pisces	Winter skate	*Leucoraja ocellata*	14	16554
Pisces	Silver hake	*Merluccius bilinearis*	13	49331
Pisces	Tiger flathead	*Platycephalus richardsoni*	13	45850

Pisces	European flounder	*Platichthys flesus*	13	35368
Pisces	Anglerfish, monkfish	*Lophius americanus*	13	20213
Pisces	Sea raven	*Hemitripterus americanus*	13	19969
Pisces	Butterfish	*Peprilus triacanthus*	13	17776
Pisces	Common dab	*Limanda limanda*	12	311140
Pisces	Yellowtail amberjack	*Seriola lalandi*	12	71908
Pisces	White hake	*Urophycis tenuis*	12	28150
Pisces	American plaice	*Pseudopleuronectes americanus*	12	17607
Pisces	Barrcouta, snake mackerel, snoek	*Thyrsites atun*	11	314274
Pisces	whiting	*Merlangius merlangus*	11	100276
Pisces	Tarakihi, jackass morwong, red moki	*Nemadactylus macropterus*	11	26179
Pisces	Windowpane flounder	*Scophthalmus aquosus*	11	16259
Pisces	Alabcore tuna	*Thunnus alalunga*	10	62793
Decapoda	American lobster	*Homarus americanus*	9	18080
Mammalia	Southern elephant seal	*Mirounga leonina*	7	224700
Pisces	Alaska Pollock	*Theragra chalcogramma*	7	32077
Aves	Black-browed albatross	*Thalassarche melanophris*	6	33108
Pisces	Coho salmon	*Oncorhynchus kisutch*	6	25856
Aves	Grey-headed albatross	*Thalassarche chrysostoma*	6	23981
Pisces	Pacific cod	*Gadus macrocephalus*	6	19003
Copepoda	Planktonic copepod	*Calanoides carinatus*	6	17856
Aves	Wandering albatross	*Diomedea exulans*	6	17586
Aves	Bufflehead sea duck	*Bucephala albeola*	5	37640
Aves	Surf scooter sea duck	*Melanitta perspicillata*	5	16639
Aves	Snow petrel	*Pagodroma nivea*	5	16133
Pisces	Pacific halibut	*Hippoglossus stenolepis*	5	15948
Aves	Adelie penguin	*Pygoscelis adeliae*	4	88678
Euphausiacea	Antarctic krill	*Euphausia superba*	4	70146
Pisces	Santer seabream	*Cheimerius nufar*	4	51962
Aves	King penguin	*Aptenodytes patagonicus*	4	24773
Aves	Glaucous winged gull	*Larus glaucescens*	4	23747
Pisces	Flathead sole	*Hippoglossoides elassodon*	4	22993
Aves	Macaroni penguin	*Eudyptes chrysolophus*	4	22387
Pisces	Arrow-tooth flounder	*Atheresthes stomias*	4	19773
Aves	Cape gannet	*Sula capensis*	3	86674
Pisces	Geelbeck croaker	*Atractoscion aequidens*	3	62430
Pisces	Slinger seabream	*Chrysoblephus puniceus*	3	42563
Pisces	White stumpnose	*Rhabdosargus globiceps*	3	32399
Aves	Western gull	*Larus occidentalis*	3	17067
Pisces	Hottentot seabream	*Pachymetopon blochii*	2	117396
Pisces	Carpenter seabream	*Argyrozona argyrozona*	2	100249
Pisces	Roman seabream	*Chrysoblephus laticeps*	2	66376
Pisces	Panga seabream	*Pterogymnus laniarius*	2	56342
Aves	Emperor penguin	*Aptenodytes forsteri*	2	20438
Pisces	Red steenbras	*Petrus rupestris*	2	18229
Pisces	kingklip	*Genypterus capensis*	2	17480
Copepoda	Planktonic copepod	*Calanus agulhensis*	2	16248
Pisces	Englishman seabream	*Chrysoblephus anglicus*	2	16100
Pisces	Red stumpnose seabream	*Chrysoblephus gibbiceps*	2	15703

Supplementary Table 3. The 100 species that occurred in most of the 5° cells, and the total number of squares they were present in. See Supplementary **Table** 2 for common names.

Classification: *Species***: Number of 5° cells present**

Planktonic Foraminifera: *Globigerinita glutinata:* 589
Planktonic Foraminifera: *Globigerina bulloides:* 574
Planktonic Foraminifera: *Neogloboquadrina dutertrei:* 550
Planktonic Foraminifera: *Orbulina universa:* 491
Planktonic Foraminifera: *Globigerinella siphonifera:* 480
Planktonic Foraminifera: *Globorotalia inflata:* 455
Planktonic Foraminifera: *Globigerinoides ruber:* 435
Planktonic Foraminifera: *Beella digitata:* 434
Planktonic Foraminifera: *Globorotalia scitula:* 426
Planktonic Foraminifera: *Globigerina falconensis:* 419
Planktonic Foraminifera: *Globigerina calida:* 414
Planktonic Foraminifera: *Globigerinoides sacculifer:* 385
Planktonic Foraminifera: *Globorotalia crassaformis:* 385
Planktonic Foraminifera: *Pulleniatina obliquiloculata:* 385
Planktonic Foraminifera: *Globigerinoides conglobatus:* 383
Planktonic Foraminifera: *Globigerina quinqueloba:* 371
Planktonic Foraminifera: *Globigerinoides tenellus:* 331
Planktonic Foraminifera: *Globigerina rubescens:* 329
Planktonic Foraminifera: *Globorotalia menardii:* 306
Copepoda: *Oithona similis:* 299
Planktonic Foraminifera: *Sphaeroidinella dehiscens:* 291
Phytoplankton: *Nitzschia seratia:* 288
Planktonic Foraminifera: *Globorotalia hirsuta:* 278
Pelagic arrow worms: *Eukrohnia hamata:* 276
Planktonic Foraminifera: *Globorotalia truncatulinoides:* 276
Phytoplankton: *Pseudo-nitzschia delicatissima:* 274
Phytoplankton: *Cylindrotheca closterium:* 270
Phytoplankton: *Thalassionema nitzschioides:* 264
Copepoda: *Metridia lucens:* 259
Phytoplankton: *Rhizosolenia styliformis:* 255
Bird: *Oceanites oceanicus:* 254
Bird: *Diomedea exulans:* 253
Mammal: *Physeter macrocephalus:* 253
Phytoplankton: *Corethron criophilum:* 253
Planktonic Foraminifera: *Globigerinella calida:* 247
Bird: *Procellaria aequinoctialis:* 245
Planktonic Foraminifera: *Neogloboquadrina pachyderma:* 243
Phytoplankton: *Thalassiothrix longissima:* 242
Pelagic deep-sea fish: *Chauliodus sloani:* 235
Planktonic Foraminifera: *Globorotalia tumida:* 232
Phytoplankton: *Proboscia alata:* 230
Bird: *Thalassarche melanophris:* 227
Phytoplankton: *Ceratium fusus:* 227
Bird: *Macronectes giganteus:* 221
Mammal: *Balaenoptera physalus:* 221
Bird: *Puffinus griseus:* 217
Mammal: *Megaptera novaeangliae:* 216
Pelagic arrow worms: *Flaccisagitta enflata:* 215
Planktonic snail: *Limacina retroversa:* 214
Krill: *Euphausia superba:* 211
Pelagic arrow worms: *Pterosagitta draco:* 211
Pelagic arrow worms: *Flaccisagitta hexaptera:* 210
Krill: *Thysanoessa macrura:* 208
Mammal: *Mirounga leonina:* 207
Mammal: *Orcinus orca:* 207
Pelagic arrow worms: *Krohnitta subtilis:* 201
Phytoplankton: *Leptocylindrus danicus:* 200
Bird: *Daption capense:* 199
Mammal: *Balaenoptera acutorostrata:* 199
Phytoplankton: *Skeletonema costatum:* 199
Pelagic arrow worms: *Aidanosagitta regularis:* 198
Pelagic arrow worms: *Sagitta bipunctata:* 198
Pelagic arrow worms: *Serratosagitta pacifica:* 195
Pelagic arrow worms: *Ferosagitta ferox:* 194
Phytoplankton: *Leptocylindrus mediterraneus:* 192
Pelagic arrow worms: *Aidanosagitta neglecta:* 191
Pelagic arrow worms: *Ferosagitta robusta:* 191
Phytoplankton: *Ceratium tripos:* 191
Pelagic arrow worms: *Krohnitta pacifica:* 190
Phytoplankton: *Ceratium furca:* 190
Pelagic arrow worms: *Zonosagitta bedoti:* 189
Bird: *Phoebetria palpebrata:* 188
Phytoplankton: *Guinardia striata:* 188
Bird: *Sterna paradisaea:* 184
Planktonic snail: *Clione limacina:* 181
Turtle: *Caretta caretta:* 176
Bird: *Thalassarche chrysostoma:* 174
Copepoda: *Nannocalanus minor:* 170
Copepoda: *Calanus finmarchicus:* 168
Phytoplankton: *Dactyliosolen fragilissimus:* 167
Phytoplankton: *Nitzschia longissima:* 166
Bird: *Pterodroma lessonii:* 164
Bird: *Thalassoica antarctica:* 162
Bird: *Fulmarus glacialoides:* 160
Pelagic arrow worms: *Mesosagitta minima:* 160
Pelagic shrimp: *Systellaspis debilis:* 160
Copepoda: *Metridia longa:* 158
Phytoplankton: *Chaetoceros decipiens:* 157
Phytoplankton: *Ceratium macroceros:* 155
Phytoplankton: *Rhizosolenia setigera:* 155
Copepoda: *Pleuromamma robusta:* 154
Planktonic Foraminifera: *Candeina nitida:* 153
Mammal: *Tursiops truncatus:* 152
Squid: *Onychoteuthis banksii:* 151
Bird: *Fregetta tropica:* 148
Bird: *Pagodroma nivea:* 148
Bird: *Rissa tridactyla:* 148
Planktonic Foraminifera: *Turborotalita humilis:* 147
Deep-sea coral: *Madrepora oculata:* 146
Bird: *Fulmarus glacialis:* 145
Pelagic arrow worms: *Zonosagitta pulchra:* 145
Pelagic shrimp: *Sergestes sargassi:* 145
Foraminifera: *Pelagobia longicirrata:* 145
Mammal: *Globicephala melas:* 144
Phytoplankton: *Lauderia annulata:* 143
Phytoplankton: *Eucampia zodiacus:* 142
Phytoplankton: *Thalassiosira angulata:* 142
Copepoda: *Pleuromamma gracilis:* 141
Bird: *Pachyptila vittata:* 140
Phytoplankton: *Guinardia delicatula:* 140

Supplementary Table 4. The biogeographic realms proposed here (in bold) compared to the IHO Seas, largely coastal realms proposed by Ekman (1953) and Briggs and Bowen (2012), strictly coastal (Spalding et al. 2007), pelagic (Spalding et al. 2012), deep-sea abyssal and bathyal (Watling et al. 2013). Column 3 superscripts indicate their Jaccard's coefficient (e.g. 3 = 3 % similarity between 5° areas in that region) in the cluster analysis. Coastal superscripts indicate the realm that a province was a subdivision of: 2 = Temperate North Atlantic realm; 3 = Temperate North Pacific; 4 = Tropical Atlantic; 5 = Western Indo-Pacific realm; 9 = Temperate South America.

	1%	2%	3% - 6%	**Seas' group**	**Ekman**	**Briggs & Bowen**	**Coastal**	**Pelagic**	**Abyssal (*including bathyal)**
**1**	**1 Inner Baltic Sea**			Inner Baltic Sea[a]	Baltic Sea brackish region	Excluded	Baltic Sea Ecoregion[2]	Excluded	Excluded
**2**	**2 Black Sea**			Black Sea	Black Sea	Black Sea	Black Sea Province and ecoregion [2]	Black Sea	Excluded
**3**	**3 NE and NW Atlantic and Mediterranean, Arctic and North Pacific**	**3.1 NE Atlantic and Mediterranean**	**3.1.1 NE Atlantic[3]**	NE Atlantic[b]	European Boreal. Part of a North Atlantic Boreal.	Eastern Atlantic Boreal	North European Seas Province[2] with 7 ecoregions	Excluded	Excluded
**4**			**3.1.2 Arctic Europe[5]**	Norwegian Sea (in part)				Part of Arctic	Excluded
**5**			**3.1.3 Mediterranean[3]**	Mediterranean[c]	Mediterranean	Lusitanian	Mediterranean Province[2] with 7 ecoregions	Mediterranean	
**6**		**3.2 Arctic and North Pacific**	**3.2.1 Arctic [3]**	Arctic seas[d]	Arctic	Arctic	1. Arctic Realm. No provinces. 19 ecoregions. Includes East Siberian Sea and Bering Sea	Part of Arctic	Arctic *
**7**			**3.2.2 North Pacific[3]**	North Pacific[e]	North West American; extends to include California. Excludes Sea of Okhotsk but notes limited data.	Eastern and Western North Pacific regions	3. Temperate North Pacific realm. Includes 4 provinces with these seas plus East China Sea	Sub-Arctic Pacific	North Pacific
**8**		**3.3 North Atlantic boreal and sub-Arctic from Canada to Greenland Sea**		North American Boreal[f]	North American Boreal. With Europe, part of a North Atlantic Boreal.	Western Atlantic Boreal	Arctic realm includes Baffin Bay, Davis Strait, Hudson Bay & Strait, Labrador Sea. Temperate North Atlantic realm includes Bay of Fundy and Gulf of St Lawrence	North Atlantic Current and Sub-Arctic Atlantic	Excluded
**9**	**4 Mid-tropical North Pacific Ocean**				Subregion 4 of Indo-west Pacific	Hawaiian	7. Eastern Indo-Pacific realm. 6 provinces, 12 ecoregions	North Pacific Current and Part of North Central Pacific	North Central Pacific

**10**	**5 South-east Pacific**					Easter Island, Marquesas	Excluded as not coastal	Part of South Central Pacific	Chile, Peru, Guatemala
**11**	**6 Mid-Atlantic, Pacific and Indian Oceans including coastal tropics and warm-temperate areas**	**6.1 Tropical West Atlantic and Tropical East Pacific**	**6.1.1 Tropical West Atlantic** [3]	Caribbean & Gulf of Mexico	West Indian. Tropical Atlantic America from Cape Hatteras to Rio de la Plata.	Western Atlantic	Tropical Northwestern Atlantic Province[4]	Inter-American Seas	Part of North Atlantic
**12**			**6.1.2 Tropical Eastern Pacific** [3]	Gulf of California	Tropical Pacific America (coast of Mexico to Galapogos Islands)	Eastern Pacific	8. Tropical Eastern Pacific realm (2 provinces, 1 ecoregions) and Warm temperate northeast Pacific province [3]	Part of East Tropical Pacific	Excluded
**13**		**6.2 Coastal Indian Ocean and West Pacific,<br><br>from Arabian Gulf to New Caledonia, southern Pacific tropical islands, and northern, western and eastern Australia.**	**6.2.1 Tropical Indo-Pacific (East Indies) and coastal Indian Ocean**[3]	Indo-Pacific seas [g] and Indian Ocean[h]	Indo-West Pacific. Subdivides into 6 sub-regions. These seas fall into the sub-regions: (1) Indo-Malayan (South China and Philippines to Australia); and Sub-region (5): Indian Ocean including Persian Gulf and Red Sea.	Tropical Indo-west Pacific	5 & 6. Western and Central Indo-Pacific realms. 19 provinces, 47 ecoregions.	South China Sea, Somali Current and Indonesia Flow-through	parts in West Pacific
**14**			**6.2.2 Red Sea** [4]	Gulfs of Aqaba, Aden, and Suez; Red Sea		Red Sea province	Red Sea & Gulf of Aden province	Red Sea	Excluded
**15**			**6.2.3 Tasman Sea into SW Pacific** [3]	Tasman Sea	Subregion (6): Tropical and subtropical Australia,	No region distinguished	Excluded as Tasman Sea is not coastal.	South-West Pacific	Part in Indian
**16**			**6.2.4 Sub-tropical Australia and Coral Sea** [4]	Coral Sea			Ecoregion in Tropical Southwestern Pacific province[5].		Excluded
**17**		**6.3 Mid South Tropical Pacific**			Subregion (3) Central Pacific Islands	Part of the Indo-west Pacific	Excluded	Part of South Central Pacific	South Pacific
**18**		**6.4 Open**	**6.4.1 Offshore**			No region	Excluded	Gulf Stream and	North

		**Atlantic, Indian, and Pacific oceans**	**North Atlantic**[4]			distinguished		North Atlantic Current	Atlantic
19			**6.4.2 Offshore Indian Ocean**[5]			No region distinguished	Excluded	Southern and Northern Indian Ocean provinces	Indian
20			**6.4.3 Offshore west Pacific**[6]		Indo-West Pacific Sub-region (2) subtropical Japan.	Western Pacific	Excluded	North Central Pacific	West Pacific
21			**6.4.3 Offshore South Atlantic** [6]			Excluded	Excluded	Equatorial Atlantic and North and South Central Atlantic	Brazil, Argentine , Sierra Leone to Angola
22			**6.4.4 Offshore mid-eastern Pacific** [7]			Excluded	Excluded	Eastern Tropical Pacific	Equatoria l Pacific
23			**6.4.5 Tropical East Atlantic**[6]	Gulf of Guinea	Tropical West Africa	Tropical Eastern Atlantic	Gulf of Guinea province [4]	Guinea Current	Excluded
24		**6.5 Southern South America**	**6.5.1 Argentina** [3]	Rio de La Plata		Argentinian	Warm temperate southwestern Atlantic province [9] (4 provinces)	Malvinas Current	Excluded
25			**6.5.2 Chile**[3]		Humboldt Current (temperate Pacific South America)	Warm-temperate Peru-Chilean	Warm temperate southeastern and Magellanic Provinces [9] (4 ecoregions)	Humboldt Current	Excluded
26		**6.6 Southern Africa, south Australia, and New Zealand**	**6.6.1 South Australia**[6]	South Australia[i]	Southern Australia	SW and SE Australia and Tasmanian	11. Temperate Australasia realm: 4 provinces and 10 ecoregions	Southern Tropical front	Excluded
27			**6.6.2 Southern Africa**[5]		Southern Africa	Agulhas and Benguela	10. Temperate South Africa realm (3 provinces, 4 ecoregions)		Excluded
28			**6.6.3 New Zealand**[6]		New Zealand	Kermadec, New Zealand and Auckland Islands	11. Temperate Australasia realm: 2 provinces 7 ecoregions		New Zealand Kermadec *
29	**7 North West Pacific**			NW Pacific [j]	North East Asia	Western Pacific	Cold Temperate Northwest Pacific province [3] (see Arctic)	Sea of Japan and Kuroshio-Oyashio Current	Excluded
30	**8 Southern Ocean**			Southern Ocean	Antarctic	Antarctic	12. Southern Ocean realm (4 provinces, 21 ecoregions)	Antarctic, Antarctic Polar Front and Sub-Antarctic	Antarctic, Sub-Antarctic *

(a) Inner Baltic Sea = Gulf of Bothnia, Gulf of Finland, Gulf of Riga;
(b) North East Atlantic = Baltic Sea, Bay of Biscay, Bristol Channel, Celtic Sea, Irish Sea & St Georges Channel, Kattegat, North Sea, Seas of W Scotland, Skaggerak;
(c) Mediterranean =Adriatic Sea, Aegean Sea, Alboran Sea, Balaeric Sea, Ionian Sea, Ligurian Sea, Mediterranean West, Mediterranean East, Strait of Gibraltar, Tyrrhenian Sea;
(d) Arctic = Arctic Ocean, Barents Sea, Beaufort Sea, Chukchi Sea, Greenland Sea, Kara Sea, Laptev Sea, White Sea;
(e) North Pacific = Bering Sea, East Siberian Sea, Gulf of Alaska, SE Alaska & British Columbia, Sea of Okhotsk;
(f) North Pacific Boreal = Baffin Bay, Bay of Fundy, Davis Strait, Gulf of St-Lawrence, Hudson Bay, Hudson Strait, Labrador Sea, North-western Passages;
(g) Indo-Pacific Seas =Andaman or Burma Sea, Arafura Sea, Bali Sea, Banda Sea, Bismarck Sea, Ceram Sea, Celebes Sea, Flores Sea, Gulf of Boni, Gulf of Thailand, Halamahara Sea, Java Sea, Makassar Strait, Malacca Strait, Philippine Sea, Molukka Sea, Savu Sea, Singapore Strait, Solomon Sea, South China Sea, Sulu Sea, Timor Sea;
(h) Indian Ocean = Arabian Sea, Bay of Bengal, Indian Ocean, Laccadive Sea, Gulf of Oman, Mozambique Channel, Persian Gulf;
(i) South Australia = Bass Strait, Great Australian Bight;
(j) North West Pacific = Eastern China Sea, Inland Sea, Japan Sea, Yellow Sea.

Data used from the Ocean Biogeographic Information System (OBIS).

Species records dated from the early 19th century with most since the 1950's. The datasets with most (over 1 million) distribution records concerned fishery, plankton and seabird data, whereas the most species rich datasets were from museum collections, regional species inventories, and global species databases that compiled data from the literature (e.g. Fishbase, Hexacorallia) (Table S1, S2). Only about 10 % of datasets had a global geographic scope.

Supplementary Table 5. The names, number of distribution records and taxa, for the datasets in OBIS as reported in the metadata on the OBIS website. Datasets are ranked by number of taxa. Taxa includes all names applied, including 'species' reported in form Genus A, or Genus sp..

Data source name	Total distribution records	Total number taxa
NMNH Invertebrate Zoology Collections (Smithsonian Institute-Invertebrate)	533822	39547
IndOBIS, Indian Ocean Node of OBIS (IndOBIS)	48349	21532
Biodiversity of the Gulf of Mexico Database (BioGoMx) (USOBIS)	126292	13549
Fishbase occurrences hosted by GBIF-Sweden (FishBase)	505852	11950
NMNH Vertebrate Zoology Fishes Collections (Smithsonian Institute-Fishes)	114408	10340
Benthic species from the tropical Pacific surrounding New Caledonia	58156	8175
Taxonomic Information Sytem for the Belgian coastal area (EurOBIS)	24624	7399
Hexacorallians of the World	64518	7017
EPA'S EMAP Database	173109	5831
Australian Museum (OBIS Australia)	118181	5384
South African Institute for Aquatic Biodiversity - Fish Collection (AfrOBIS)	44343	4434
Marine and Coastal Research Institute - INVEMAR, Colombia, IABIN	34733	4131
NBI (NOAA NBI)	171033	4028
Natal Museum - Mollusc Collection (AfrOBIS)	26516	4006
SeamountsOnline (Seamount Biota)	18632	3720
Marine Nature Conservation Review (MNCR) and associated benthic marine data held and managed by JNCC (EurOBIS)	580008	3619
South Western Pacific Regional OBIS Data All Sea Bio Subset	42180	3611
Ifremer BIOCEAN database (Deep Sea Benthic Fauna)	24408	3163
NODC WOD01 Plankton Database	1275382	3128
SeSaM (EurOBIS)	21445	3126
CeDAMar database for benthic biological sampling on the abyssal plains: European data (EurOBIS)	12335	3054
iziko South African Museum - Crustacean Collection (AfrOBIS)	13123	3004
iziko South African Museum - Mollusc Collection (AfrOBIS)	6019	2915
Gwaii Haanas Invertebrates (OBIS Canada)	23315	2828
Bishop Museum Data (OBIS distribution) (USOBIS)	7998	2820
iziko South African Museum - Fish Collection (AfrOBIS)	15136	2796
Atlantic Reference Centre (OBIS Canada)	125272	2762
Natural Geography In Shore Areas (NaGISA) Dataset (NaGISA)	47732	2634
Pembrokeshire Marine Species Atlas (EurOBIS)	42591	2394
Bay of Fundy Species List (OBIS Canada)	2381	2371
Academy of Natural Sciences OBIS Mollusc Database	16201	2333
MedOBIS (EurOBIS)	33932	2242
Southampton Oceanography Center Discovery Collections Midwater Database	92851	2046
MV Marine Invertebrates (OBIS Australia)	19446	1965
Seasearch Marine Surveys (EurOBIS)	159873	1898
Marine Life Information Network (MarLIN) marine survey data (Professional) (EurOBIS)	129573	1895
Generic Taxonomic Database System on Mysida and Nematoda	3616	1867
Benthic biodiversity along the central coast in the Brazilian EEZ (OBIS South	6998	1605

America, BRAZIL) (WSAOBIS)		
A Biological Survey of the Waters of Woods Hole and Vicinity	46294	1553
Marine benthic dataset (version 1) commissioned by UKOOA (EurOBIS)	175360	1532
University of Costarica (UCR) Fish Database (costarica_fish)	12370	1421
BioMar - Ireland: benthic marine species survey (EurOBIS)	93003	1397
DASSH Data Archive Centre Academic surveys (EurOBIS)	62099	1359
UW Fish specimens	86025	1319
Canadian Museum of Nature - Fish Collection (OBIS Canada)	29877	1302
CSIRO Marine Data Warehouse (OBIS Australia)	106342	1285
Shorefishes of the Tropical Eastern Pacific Online Information System (SFTEP) (Smithsonian Tropical Research Institute)	63632	1227
MV Ichthyology (OBIS Australia)	9413	1189
Marine Nature Conservation Review (MNCR) and associated benthic marine data held and managed by English Nature (EurOBIS)	13769	1189
ChEssBase (CheSS)	3729	1149
MICROBIS database (ICoMM)	885968	1138
SINBIOTA - marine data (Tropical and Subtropical Western South Atlantic OBIS)	19780	1099
Biogeographic data from BODC - British Oceanographic Data Centre (EurOBIS)	124043	982
Galathea II, Danish Deep Sea Expedition 1950-52. (The Danish Biodiversity Information Facility)	1822	975
Offshore ref. stations, Norwegian/Barents Sea (EurOBIS)	46377	939
Marine Nature Conservation Review (MNCR) and associated benthic marine data held and managed by CCW (EurOBIS)	13897	886
North Sea Benthos Survey (EurOBIS)	16838	872
REVIZEE Score Sul / Bentos (WSAOBIS)	2810	782
Marine Life List of Ireland (EurOBIS)	6000	762
Northeast Fisheries Science Center Bottom Trawl Survey Data (USOBIS)	460938	750
Macrobenthos from the eastern English Channel in 1999 and 2001 (EurOBIS)	24357	736
NivN Bay species list, Sjlland, Denmark (The Danish Biodiversity Information Facility)	770	734
Seaweed data for Great Britain and Ireland (EurOBIS)	111682	733
Marine and Coastal Management - Demersal Surveys (AfrOBIS)	201741	684
Marine RAP 38 Bra (Tropical and Subtropical Western South Atlantic OBIS)	4059	681
MAR-ECO 2004 (EurOBIS)	9500	680
The Southeast Regional Taxonomic Center (USOBIS)	2780	674
Australian Institute of Marine Science - Baited Remote Underwater Video Stations (BRUVS). (Australian Institute of Marine Science)	18540	673
CRED Rapid Ecological Assessments of Fish Belt Transect Surveys and Fish Stationary Point Count Surveys in the Pacific Ocean (USOBIS)	332603	664
WA Museum Ningaloo Marine Invertebrate Zoology database (via OBIS Australia) (OBIS Australia)	1942	640
Weddell Sea macrozoobenthos EASIZ I (SCAR-MarBIN)	4647	638
Universidad Simon Bolivar Museum of Natural Sciences (USB-MCN)	10654	637
Marine Biodiversity in Ilha Grande Bay Rio de Janeiro State - Southwest Brazil (Tropical and Subtropical Western South Atlantic OBIS)	7012	633
Cold Water Corals	6553	612
MarBEF Publication Series data (EurOBIS)	1777	609
Marine Nature Conservation Review (MNCR) and associated benthic marine data held and managed by Scottish Natural Heritage (EurOBIS)	16531	607
South Western Pacific Regional OBIS Data Bryozoan Subset (South Western Pacific OBIS)	6348	599
Marine Life Survey Data (collected by volunteers) collated by MarLIN (EurOBIS)	10046	589
Australian Institute of Marine Science - Bioresources Library	1093	582
Historical hyperbenthos data (1987-2001) from the North Sea and some adjacent areas (EurOBIS)	35153	580
Marine Benthic Fauna List, L, Denmark (The Danish Biodiversity Information Facility)	577	576
Centro de Estudos do Mar - CEM, UFPR (Tropical and Subtropical Western South	5590	571

Atlantic OBIS)		
HMAP-History of Marine Animal Populations (CoML)	313587	569
Survey of North Wales and Pembrokeshire Tide Influenced Communities (EurOBIS)	6895	560
Macrobenthos from English waters between 2000-2002 (EurOBIS)	3999	545
Australian Institute of Marine Science - CReefs Ningaloo Reef Biodiversity Expedition (Australian Institute of Marine Science)	1582	522
SAM Ichthyology (OBIS Australia)	3286	512
Macrobenthos from the Norwegian waters (EurOBIS)	14891	512
Bolus Herbarium Algal Specimen Database (AfrOBIS)	9664	504
Australian Institute of Marine Science - Lizard Island Reef Biodiversity Expedition (Australian Institute of Marine Science)	1085	493
Macrobenthos samples collected in the Scottish waters in 2001 (EurOBIS)	4681	486
Mollusc (marine) data for Geat Britain and Ireland (EurOBIS)	37961	477
Historical benthosdata from the North Sea and Baltic Sea from 1902-1912 (EurOBIS)	6399	473
Video Annotation and Reference System (VARS) database (USOBIS)	176673	473
Southeast BR Mangrove (Tropical and Subtropical Western South Atlantic OBIS)	2468	468
Offshore reference stations (Finnmark) (EurOBIS)	9669	462
iziko South African Museum - Shark Collection (AfrOBIS)	14484	454
Historical benthic data from the southern Baltic Sea (1839-2001) (EurOBIS)	41422	451
The Deepwater Program: Northern Gulf of Mexico Continental Slope Habitat and Benthic Ecology - DgoMB: Polys	3380	444
South American Antarctic Marine Biodiversity Literature (SCAR-MarBIN)	905	443
BIS dataset of the south-western part of Netherlands (1985-2004) (EurOBIS)	136161	442
Phytoplankton from the White Sea, Barents Sea, Norwegian Sea and Arctic Basin 1993-2003 (ArcOD/AOOS)	37325	416
Gwaii Haanas Marine Plants (OBIS Canada)	6351	412
NCOS1959_Crustacea (OBIS China)	25793	410
Laboratory of the Ocean Bottom Fauna, P.P.Shirshov Institute of Oceanology of Russian Academy of Science. (Comarge_Shirshov)	4040	408
Continuous Plankton Recorder database (SAHFOS)	2533649	406
HamPelFish (EurOBIS)	7138	396
A comparison of benthic biodiversity in the North Sea, English Channel and Celtic Seas (EurOBIS)	2588	395
ICES Database of trawl surveys (EurOBIS)	4609303	390
Bureau of Rural Sciences National commercial fisheries half-degree data set 2000-2002 (OBIS Australia)	60445	389
CRED Rapid Ecological Assessment of Invertebrate in the Pacific Ocean (USOBIS)	64435	387
Catalogue of Squat Lobsters (SquatLobsters)	602	379
Australian Institute of Marine Science, Long-term monitoring Program: Nearshore corals of the Great Barrier Reef. (Australian Institute of Marine Science)	8906	377
Macrozoobenthos data from the southeastern North Sea in 2000 (EurOBIS)	10283	376
Macro- and megafauna from the North Aegean Sea from 1997-1998 (EurOBIS)	6402	370
NOAA HML Tidal Creek Database	6307	366
South Western Pacific Regional OBIS Data Bio Ross Subset (South Western Pacific OBIS)	1166	360
Free-living marine nematodes from the Southern Bight of the North Sea (EurOBIS)	7521	360
SPF Collection of Sao Paulo State (Tropical and Subtropical Western South Atlantic OBIS)	4632	358
Pacific Shrimp Trawl Survey (OBIS Canada)	128809	356
Southeast Area Monitoring and Assessment Program (SEAMAP) South Atlantic (USOBIS)	65488	356
Volunteer sightings data held by the DASSH Data Archive Centre (EurOBIS)	4734	354
Benthic fauna in the Pechora Sea (EurOBIS)	1324	352
Offshore ref. stations, North/Norwegian sea (EurOBIS)	7959	337
CephBase	3172	328

Coleccin Ictiolica Del Instituto Nacional de Investigacin y Desarrollo Pesquero (INIDEP), Argentina - Ichthyologic Collection of the National Research Institute and Fishery Development (INIDEP) of Argentina (Argentinean RON)	720	321
REVIZEE Central Coast Deep Ocean (Tropical and Subtropical Western South Atlantic OBIS)	426	320
Antarctic Amphipod Crustaceans: Ant'Phipoda Database (BIANZO) (SCAR-MarBIN)	6702	318
AAD Benthic Sampling Database (Australian Antarctic Data Centre)	1357	317
WA Museum Ningaloo Crustacea database (via OBIS Australia) (OBIS Australia)	918	315
Taxonomically comprehensive assessment of biodiversity of animal plankton throughout the world ocean (CMarZ)	130399	315
North BR Mangrove (Tropical and Subtropical Western South Atlantic OBIS)	1041	313
Benthic Fauna in the Barents Sea (EurOBIS)	1410	312
Macrobel: Long term trends in the macrobenthos of the Belgian Continental Shelf (EurOBIS)	21086	310
Polycystine Radiolarians from the water column and the surface sediments of the World Ocean (Argentinean RON)	11626	308
Antarctic Echinoids: an interactive database (SCAR-MarBIN)	1619	307
(Zoological Museum Amsterdam) Noordzee (EurOBIS)	35886	304
Deep-sea Meiobenthos (EurOBIS)	1583	303
Nematodes from the NSBS (EurOBIS)	1057	298
SO-Polylist (SCAR-MarBIN)	4583	290
Australian Institute of Marine Science - Great Barrier Reef nearshore coral diversity. (Australian Institute of Marine Science)	16489	285
Benthic fauna around Franz Josef Land (EurOBIS)	1714	285
North Pacific Groundfish Observer (North Pacific Research Board)	422150	280
DASSH Data Archive Centre expert sighting records (EurOBIS)	781	280
CRED Rapid Ecological Assessment of Benthic Habitat Cover in the Pacific Ocean (USOBIS)	37804	277
ECNASAP - East Coast North America Strategic Assessment (OBIS Canada)	466736	273
L4 Plankton Monitoring Programme (EurOBIS)	49597	266
Estuarine Demersal Fish of Brazil (Tropical and Subtropical Western South Atlantic OBIS)	2889	265
Brazilian Marine Invertebrate Data Sets from SpeciesLink (Tropical and Subtropical Western South Atlantic OBIS)	2203	263
SPEEK database: Meiobenthos of subtidal sandbanks on the Belgian Continental Shelf (EurOBIS)	8814	260
SMCC Gulf of Maine Invertebrate Data (USOBIS)	1688	258
CRED REA Algal Quadrate Images in the Pacific Ocean (USOBIS)	27174	254
macrobenthos in the Dutch Sector of the North Sea 1991-2001 (EurOBIS)	4663	253
Macrobenthos data from the Norwegian Skagerrak coast (EurOBIS)	1918	249
NCOS1959_Mollusca (OBIS China)	16007	248
Cefas01 - Structure of sublittoral nematode assemblages around the UK coast (EurOBIS)	2222	244
Structures and Nutrition Requirements of Macrozoobenthic Communities in the area of the Lomonossov Ridge, 1995-1998 (ArcOD/AOOS)	1677	243
Environmental Benchmark Studies in Casco Bay-Portland Harbor, Maine, April 1980 (CascoBay)	1845	238
Australian Institute of Marine Science, Long-term monitoring Program: Visual Census Fish Data (Great Barrier Reef). (Australian Institute of Marine Science)	41695	236
Marine Biota Along the West Coast of Ceara State - Northeast Brazil (Tropical and Subtropical Western South Atlantic OBIS)	770	236
USGS 2001 Buck Island National Monument Cryptic Fish Survey (USOBIS)	2609	224
Biogeography Scheldt Estuary (EurOBIS)	31747	223
DFO Maritimes Research Vessel Trawl Surveys Fish Observations (OBIS Canada)	140783	223
Benthic marine algae from Cabo Frio (Tropical and Subtropical Western South Atlantic OBIS)	2722	222
ICES Biological community (EurOBIS)	17557	222

National Institute of Marine Sciences and Technologies - Trawl Surveys (AfrOBIS)	7664	221
MNA - Sezione di Genova - (Marine Biological Samples) (SCAR-MarBIN)	638	218
Electron Micrograph Database (Australian Antarctic Data Centre)	1358	217
Northern Barrier Marine Life of the Great Barrier Reef. (Australian Institute of Marine Science)	869	216
ZooplanktonBeaufortSeaNOGAP2 (ArcOD/AOOS)	9366	215
Checklist of benthic marine algae and cyanobacteria of northern Portugal (EurOBIS)	1755	212
IOW Macrozoobenthos monitoring Baltic Sea (1980-2005) (EurOBIS)	3589	206
A Historical Record of Sponges, Bryozoa and Ascidians on the Coast of Maine:1843-1980 (Bigelow Laboratory for Ocean Sciences)	623	205
On the composition of the benthic fauna of the western Fram Strait (ArcOD/AOOS)	850	205
JNCC seabird distribution and abundance data (all trips) from ESAS database (OBIS-SEAMAP)	1122883	204
The Deepwater Program: Northern Gulf of Mexico Continental Slope Habitat and Benthic Ecology - DgoMB: Trawls	7062	204
Free-living nematodes of the Voordelta (EurOBIS)	2611	203
Marine and Coastal Management - Linefish Dataset (AfrOBIS)	2744958	202
The meiobenthos of the Southern Bight of the North Sea (EurOBIS)	1299	202
PIROP Northwest Atlantic 1965-1992 (OBIS-SEAMAP)	209039	194
Study of the meiobenthos from a dumping site in the Southern Bight of the North Sea (EurOBIS)	1495	194
The Macrobenthos of Penobscot Bay, Maine (Bigelow Laboratory for Ocean Sciences)	1640	193
REVIZEE South Score / Pelagic and Demersal Fish Database (WSAOBIS)	1888	191
REVIZEE South Score / Pelagic and Demersal Fish Database II (WSAOBIS)	4129	188
South BR Mangrove (Tropical and Subtropical Western South Atlantic OBIS)	1568	186
National Marine Monitoring Programme data set (EurOBIS)	1161	181
Phytoplankton Universidad Arturo Prat (ESPOBIS)	22499	180
ZooplanktonBeaufortSeaNOGAP1 (ArcOD/AOOS)	8058	179
Nematoda from Kenya and Zanzibar (EurOBIS)	6627	179
ZooplanktonNOGAP32b1986 (ArcOD/AOOS)	11090	178
Svalbard Tidal Zone data (EurOBIS)	1400	178
Antarctic pycnogonids (SCAR-MarBIN)		174
Pelagic Fish Observations 1968-1999 (Australian Antarctic Data Centre)	25940	170
Marine fauna survey of the Vestfold Hills and Rauer Island, 1981-82 (Australian Antarctic Data Centre)	359	170
IBSS historical data from different cruises (EurOBIS)	62381	170
Cefas05 - Structure of nematode communities in the southwestern North Sea (EurOBIS)	2769	168
Darwin Mounds (EurOBIS)	2858	168
CalCOFI and NMFS Seabird and Marine Mammal Observation Data, 1987-2006 (OBIS-SEAMAP)	70426	168
Corbisier 1991 1994 Benthic Macrofauna (Tropical and Subtropical Western South Atlantic OBIS)	2576	167
KOBIS database (KOBIS)	3184	166
Eastern Channel dataset (EurOBIS)	493	166
Benthos Gironde Estuary (EurOBIS)	3019	165
N3 data of Kiel bay (EurOBIS)	8944	164
Arctic soft-sediment macrobenthos (EurOBIS)	1004	164
Cross Sands broadscale survey 1998 (EurOBIS)	557	164
Australian Institute of Marine Science CReefs: Heron Island Biodiversity Expedition (Australian Institute of Marine Science)	265	163
COMARGIS: Information System on Continental Margin Ecosystems (comarge)	779	162
Diatom and foraminiferal samples from surficial sediments of Prydz Bay, Antarctica (Australian Antarctic Data Centre)	828	162
Registered benthic Invertebrata held at the Australian Museum	1584	159

(Ozcam_AustralianMuseum)		
Meiobenthos of subtidal sandbanks on the Belgian Continental Shelf (EurOBIS)	6458	158
South Western Pacific Regional OBIS Data Asteroid Subset (South Western Pacific OBIS)	2294	156
Fishes in the Argentine Sea from 1967 to the present time (Argentina-Ictio)	5426	156
Nematodes from the Weddell Sea (EurOBIS)	960	153
CRED Rapid Ecological Assessments of Coral Population in the Pacific Ocean (USOBIS)	56964	153
NCOS1959_Echinodermata (OBIS China)	7723	152
Fish larvae biodiversity along the central coast in the Brazilian EEZ (OBIS South America, BRAZIL) (WSAOBIS)	2562	150
Distribution of nematodes in Patagonia Argentina coast (Argentina-Nematodes)	1270	149
DinoTintinideos (Tropical and Subtropical Western South Atlantic OBIS)	947	148
Characteristic features of the benthic algal vegetation along the Snaefellsnes peninsula (EurOBIS)	1487	148
Macrobenthic species of the Eastern South Pacific (ESPOBIS)	573	146
Cefas04 - Impacts of chronic trawling disturbance on nematode communities (EurOBIS)	3383	145
Meiobenthos at the stations 115, 702, 790 on the Belgian Continental Shelf (EurOBIS)	4276	145
CRED Towed-Diver Fish Biomass Surveys in the Pacific Ocean (USOBIS)	20957	145
IPOE_Benthos_Steffens (ArcOD/AOOS)	481	144
Zooplankton biodiversity along the central coast in the Brazilian EEZ (OBIS South America, BRAZIL) (WSAOBIS)	3670	142
Survey of the benthic algal vegetation of the Berufjrdur, southeastern Iceland (EurOBIS)	1602	142
Kongsfjorden monitoring data - grid - 2006 (EurOBIS)	949	141
TROPHOS/PODO-I work-database I (23/01/2004): Meiobenthos from station 330 - structural and funtional biodiversity on the Belgian Continental Shelf (EurOBIS)	2848	140
Cefas03 - Impacts of experimental trawling disturbance on nematode communities (EurOBIS)	3041	139
Intertidal rocky shore assemblages in Portugal (EurOBIS)	7164	135
Australian Institute of Marine Science - Summer planktonic communities of North West Cape, Western Australia. (Australian Institute of Marine Science)	1360	134
The Deepwater Program: Northern Gulf of Mexico Continental Slope Habitat and Benthic Ecology - DgoMB: Fishs	222	133
CMarZ (Census of Marine Zooplankton)-Asia Database (CMarZ)	2851	132
Heraklion Harbour Meiobenthos (EurOBIS)	1012	130
Chukchi/Bering Sea Zooplankton (ISHTAR), 1985-1989 (ArcOD/AOOS)	37218	130
Davis Strait and Baffin Bay Zooplankton (OBIS Canada)	9767	129
Benthic algal vegetation of Borgafjrdur (EurOBIS)	1060	129
A study of the nematode fauna of three estuaries in the Netherlands (EurOBIS)	957	129
Seabirds of the Southern and South Indian Ocean (Australian Antarctic Data Centre)	149396	128
Size Indian Nematodes (EurOBIS)	493	128
Maine Department of Marine Resources Inshore Trawl Survey	22960	127
Benthic algal vegetation of Mjifjrdur (EurOBIS)	711	127
Macroalgae of the Tjrnes Peninsula in the North of Iceland (EurOBIS)	2540	126
Southern Ocean Continuous Zooplankton Recorder (SO-CPR) Survey (Australian Antarctic Data Centre)	95519	125
BenthosChukchiFN762_1976_Falk5 (ArcOD/AOOS)	1809	125
Centro Nacional Patagonico Ichthyological Collection (Argentinean RON)	1199	125
Plymouth Sound macrofauna (EurOBIS)	1343	124
Cefas06 - Effects of various types of disturbances on nematode communities (EurOBIS)	1146	124
Aegean Sea Bathyal Nematodes (EurOBIS)	1017	123
WA Museum Ningaloo Mollusca database (via OBIS Australia) (OBIS Australia)	766	121
MV Ornithology (OBIS Australia)	8918	120

Cefas02 - Structure of sublittoral nematode assemblages at four offshore stations around the UK (EurOBIS)	1331	120
Electronic Atlas of Ichthyoplankton on the Scotian Shelf of North America (OBIS Canada)	3437	119
Copepods (Tropical and Subtropical Western South Atlantic OBIS)	2311	119
Meiofauna from the Firth of Clyde (Scotland) (EurOBIS)	442	117
Malia Nematodes (EurOBIS)	488	116
Amrum Bank and inner German Bight Benthos (EurOBIS)	1026	115
Abundance and diversity of the Amphipoda (Crustacea) from the Greenlandic shelf (ArcOD/AOOS)	4872	115
FishWesternArctic (ArcOD/AOOS)	3057	114
Promachocrinus kerguelensis (SCAR-MarBIN)		114
Meiofauna of the Gulf of Trieste-Slovenia (EurOBIS)	4774	112
Copepods from the Southern Bight of the North Sea (EurOBIS)	993	111
Macrozoobenthos from the Belgian Continental Shelf, collected in 2000 (EurOBIS)	636	111
Macrobenthos from Copale - Authie (EurOBIS)	1073	110
Macroalgal communities of intertidal rock pools in Portugal (EurOBIS)	2382	109
Meiofauna from the Goban Spur (OMEX) - 1993 (EurOBIS)	1082	109
Macrobenthos data from the Doggerbank - 2000 (EurOBIS)	566	109
Nematode fauna from the bottom of the Southern North Sea (EurOBIS)	853	108
Fish catch from 1996/97 Voyage 2 WASTE (WOCE Antarctic Southern Transect Expedition) (Australian Antarctic Data Centre)	465	108
Grand Manan Basin Benthos (OBIS Canada)	244	107
Nematodes from Italy and Poland (EurOBIS)	612	107
Liverpool Bay Nematoda and Copepoda (UK) (EurOBIS)	2041	106
RMT Trawl catch from the 1980/81 V5 FIBEX voyage (Australian Antarctic Data Centre)	2293	105
Nematode data from the Firth of Clyde (Scotland) (EurOBIS)	1299	104
Polish Arctic Marine Programme (EurOBIS)	603	103
Aerial Oil Spill Response Survey 1994-1997 (OBIS-SEAMAP)	14895	103
Nematoda and Copepoda from the Fal estuary (EurOBIS)	1617	103
Nematodes of the central Arctic Ocean (EurOBIS)	496	103
Benguela Current Large Marine Ecosystem (BCLME) - Namibia (AfrOBIS)	488	103
MMS Ship survey, SCB 1975-1978 (OBIS-SEAMAP)	23518	102
MARMAP Chevron Trap Survey (USOBIS)	15106	101
MMS Low Altitude Survey 1980-1983 (OBIS-SEAMAP)	71453	99
NCOS (OBIS China)	7956	98
Nova Scotia Museum of Natural History - Marine Birds, Mammals, and Fishes (OBIS Canada)	579	97
The Deepwater Program: Northern Gulf of Mexico Continental Slope Habitat and Benthic Ecology - DgoMB: Inverts	158	97
TROPHOS/PODO-I work-database I (23/01/2004): Meiobenthos station 115bis - bentho-pelagic coupling (EurOBIS)	4016	95
Copepods of the Equatorial Eastern Pacific (Tropical and Subtropical Eastern South Pacific OBIS)	260	94
Animal Demography Unit - The Birds in Reserves Project (BIRP) (AfrOBIS)	23226	94
Meiobenthos and nematodes from the continental shelf of the Laptev Sea (EurOBIS)	448	94
Variability of benthic Foraminifera north and south of the Denmark Strait (ArcOD/AOOS)	262	94
North American Sessile Marine Invertebrate Survey	4808	93
Meiofauna and nematodes from the Atacama Slope and Trench (EurOBIS)	425	93
Phytoplankton in the Oosterschelde before, during and after the storm-surge barrier (1982-1990) (EurOBIS)	12782	92
CMAR Albatross Bay Zooplankton 1996-98 (via OBIS Australia) (OBIS Australia)	7417	91
Antarctic Marine Species Sequence Data (SCAR-MarBIN)	295	90
Programa de Observadores a Bordo (POBCh) de la Secretaria de Pesca de la	170021	89

Provincia del Chubut, Argentina . Observer On board Program -Fisheries Secretariat of the Province of Chubut-Argentina (OOBPPCh) (Argentinean RON)		
Nematodes from Kongsfjord, Svalbard (EurOBIS)	817	89
RMT Trawl catch from the 1995/96 V4 BROKE voyage (Australian Antarctic Data Centre)	2692	88
Length and width measurements of nematodes in the Ligurian Sea (EurOBIS)	2290	88
ICES contaminants and biological effects (EurOBIS)	726975	88
Paranaguí Bay - Plankton and Benthos Database (WSAOBIS)	4500	87
Cefas10 - Effects of physical disturbance on nematodes communities in sand and mud (EurOBIS)	1196	87
Meiofauna of the Ligurian Sea (EurOBIS)	447	87
CRED Rapid Ecological Assessments of Coral Health and Disease in the Pacific Ocean (USOBIS)	3887	87
Animal Demography Unit - South African Bird Ringing Unit (SAFRING) (AfrOBIS)	164366	86
Experimental effects of TBT on meiobenthic communities (EurOBIS)	1739	86
Nematode assemblages from European sandy beaches (EurOBIS)	641	85
Museo Argentino de Ciencias Naturales (MACN), Buenos Aires: sea stars collection (Asteroidea, Echinodermata) (SCAR-MarBIN)	362	84
Collections data on ecology of bottom animal of the Southern ocean (SCAR-MarBIN)	2500	83
Copepoda of the Dutch Continental Shelf, spring 1993 (EurOBIS)	1895	83
MMS Surveys, SCB 1995-1997 (OBIS-SEAMAP)	15130	82
Spring Phytoplankton Assemblages in the Southern Ocean Between Australia and Antarctica (CLIVAR-SR3) (Australian Antarctic Data Centre)	829	81
MAR-ECO 2003 - Arni Fridriksson (EurOBIS)	1066	81
Northeast BR Mangrove (Tropical and Subtropical Western South Atlantic OBIS)	339	80
SIO Marine Bird and Mammal Survey 2004 (OBIS-SEAMAP)	4121	79
Spatial heterogeneity of nematodes on an intertidal flat in the Westerschelde Estuary (EurOBIS)	1540	79
Macrozoobenthos composition, abundance and biomass in the Arctic Ocean along a transect between Svalbard and the Makarov Basin 1991. (ArcOD/AOOS)	266	79
WhiteSeaPlankton (ArcOD/AOOS)	12499	78
iziko South African Museum - Marine Mammal Collection (AfrOBIS)	1184	77
WADFW PSAMP W1996 (OBIS-SEAMAP)	36891	77
WADFW PSAMP W1995 (OBIS-SEAMAP)	46157	77
WADFW PSAMP W1994 (OBIS-SEAMAP)	32776	77
Copepods from a sublittoral sandy station in the North Sea (EurOBIS)	438	77
Nematodes from the South Sandwich Trench (EurOBIS)	333	76
Laptev Sea and Nansen Basin Zooplankton, 1993 (ArcOD/AOOS)	6639	76
Amazon Shelf Holocene Present Benthic Foraminifera (Tropical and Subtropical Western South Atlantic OBIS)	851	76
Atlantic and Gulf Rapid Reef Assessment - Fish (OBIS Australia)	50888	75
MMS Aerial Survey, PNW 1989-1990 (OBIS-SEAMAP)	15113	75
BIOMASS 1980-1985 (OBIS-SEAMAP)	16708	74
Nematodes of the Plymouth Sound (EurOBIS)	1433	73
Intertidal Biodiversity in the Gulf of Maine (Gulf of Maine Census of Marine Life Program)	255	72
WADFW PSAMP W1998 (OBIS-SEAMAP)	31040	72
Tidal migration of nematodes on an estuarine tidal flat (EurOBIS)	1102	72
WADFW PSAMP W2003 (OBIS-SEAMAP)	35684	71
WADFW PSAMP W2004 (OBIS-SEAMAP)	31999	71
Australian Institute of Marine Science, Surveys of Octocoral communities, benthic cover and environmental factors on coral reefs of the Great Barrier Reef. (Australian Institute of Marine Science)	15875	71
The Deepwater Program: Northern Gulf of Mexico Continental Slope Habitat and Benthic Ecology - DgoMB: Isopods	10009	71
NOAA Southeast Fishery Science Center (SEFSC) Commercial Pelagic Observer	231198	70

Program (POP) Data (SEFSC_POP)		
WADFW PSAMP W1993 (OBIS-SEAMAP)	33419	69
WADFW PSAMP W1997 (OBIS-SEAMAP)	33528	69
Animal Demography Unit - Coordinated Waterbird Counts (CWAC) (AfrOBIS)	15827	69
Nematodes from the Exe Estuary (microcosm experiments) (EurOBIS)	792	69
Atlantic and Gulf Rapid Reef Assessment - Benthic (Australian Institute of Marine Science)	66248	68
WADFW PSAMP W1999 (OBIS-SEAMAP)	29015	68
RMT Trawl catch from the 1992/93 V6 KROCK voyage (Australian Antarctic Data Centre)	1815	68
Baltic Seabirds Transect Surveys (OBIS-SEAMAP)	23289	67
National monitoring of macrobenthos in the Kavala Gulf (EurOBIS)	764	67
Nematodes of Solbergstrand, Norway (in presence and absence of Brissopsis) (EurOBIS)	319	67
Nematodes at two abyssal sites in the NE Atlantic (EurOBIS)	318	67
Amphipoda Hyperiidea of the Southern Ocean: catalogue and occurrences (SCAR-MarBIN)	1009	67
RMT Trawl catch from 1985/86 V1 ADBEX III voyage (Australian Antarctic Data Centre)	556	66
The Deepwater Program: Northern Gulf of Mexico Continental Slope Habitat and Benthic Ecology - DgoMB: Amphipods	427	66
Nematodes from Crete sandy beaches (EurOBIS)	793	65
Posidonia Oceanica Survey 2005 (EurOBIS)	1933	64
WADFW PSAMP W2000 (OBIS-SEAMAP)	29550	64
Polychaeta (Tropical and Subtropical Western South Atlantic OBIS)	103	64
WADFW PSAMP W2001 (OBIS-SEAMAP)	27506	63
WADFW PSAMP W2002 (OBIS-SEAMAP)	27341	63
Diatoms from SAZ Sediment traps (Australian Antarctic Data Centre)	306	63
SIO Marine Bird and Mammal Survey 2003 (OBIS-SEAMAP)	2924	62
IPOE_Schnack_Polychaeta (ArcOD/AOOS)	566	61
PANGAEA - Publishing Network for Geoscientific & Environmental Data (EurOBIS)	99341	60
Cefas08 - Effects of simulated deposition of dredged material on structure of nematode assemblages - the role of contamination (EurOBIS)	204	60
Australian Institute of Marine Science, Zooplankton community structure in Nearshore waters of the Great Barrier Reef. (Australian Institute of Marine Science)	397	60
Migotto 1996 Hydroids of Sao Sebastiao, SP (Tropical and Subtropical Western South Atlantic OBIS)	852	59
ANTXXIII-8 Birds and Mammals (SCAR-MarBIN)	489	59
WADFW PSAMP S1993 (OBIS-SEAMAP)	13994	58
WADFW PSAMP S1995 (OBIS-SEAMAP)	11100	58
Cefas07 - Effects of simulated deposition of dredged material on structure of nematode assemblages - the role of burial (EurOBIS)	170	58
Free-living nematodes in a brackish tidal flat of the Westerschelde (EurOBIS)	3050	58
Marine Geoscience Data System (MGDS)	979	57
SEFSC GoMex Oceanic 1994 (OBIS-SEAMAP)	2478	57
SEFSC GoMex Oceanic 1993 (S) (OBIS-SEAMAP)	3196	57
Offshore nematodes from Rame and in microcosm experiment (exposure to metals) (EurOBIS)	184	57
WADFW PSAMP S1996 (OBIS-SEAMAP)	12503	56
SEFSC GoMex Oceanic 1992 (199) (OBIS-SEAMAP)	1942	56
Cefas09 - Effects of paint-derived tributyltin (TBT) on structure of estuarine nematode assemblages in experimental microcosms (EurOBIS)	177	56
Biogeographic distribution of Antarctic and sub-Antarctic Mysida (SCAR-MarBIN)	738	56
MMS Low Altitude Survey, SCB 1975-1978 (OBIS-SEAMAP)	7950	55
SEFSC GoMex Oceanic 1996 (OBIS-SEAMAP)	7098	55
Kongsfjorden/Spitsbergen - soft bottom fauna (EurOBIS)	210	55

Historical data on invertebrates from the Baltic Sea and Gdansk Bay (EurOBIS)	270	54
Bentho-pelagic coupling in the North Sea: Copepoda (EurOBIS)	762	54
Antarctic and Subantarctic Asteroid zoogeography (SCAR-MarBIN)	439	53
WADFW PSAMP S1994 (OBIS-SEAMAP)	14580	53
Canada_BasinMegabenthos2002 (ArcOD/AOOS)	177	53
ZINRAS_Arctic_Benthos (ArcOD/AOOS)	7439	53
BfG - Estuary Monitoring Programme Macrozoobenhos (EurOBIS)	286	53
70 samples data of Kiel Bay (EurOBIS)	1144	52
Biological Collection, National Institute of Oceanography, Goa, India (IndOBIS)	308	52
SEFSC GoMex Oceanic 2000 (OBIS-SEAMAP)	1243	52
Benthos Chirnov Basin 1986 and 2002 (ArcOD/AOOS)	3435	52
Zooplankton of the Eastern South Pacific (ESPOBIS)	1664	51
Rocky shore algal data from North Adriatic Sea (Piran) in 2006 (EurOBIS)	1062	51
Nematode fauna of the North Sea near the Westerschelde Estuary (EurOBIS)	702	51
The pre-winter 2007 vertical distribution of zooplankton in the Cape Bathurst and North Water polynyas, and Lancaster Sound, Canadian Arctic (ArcOD/AOOS)	3345	51
WADFW PSAMP S1998 (OBIS-SEAMAP)	6370	50
WADFW PSAMP S1997 (OBIS-SEAMAP)	6114	50
MMS Seabird Ecology Study 1985 (OBIS-SEAMAP)	2997	50
EPOS3: SeaStars (Echinodermata, Asteroidea) (SCAR-MarBIN)	293	50
SEFSC Caribbean Survey 2000 (OBIS-SEAMAP)	835	49
Azooxanthellate Scleractinia Brazil 01 (Tropical and Subtropical Western South Atlantic OBIS)	358	49
Copepoda from the Middelkerke bank (North Sea) (EurOBIS)	248	49
Antarctic Jellyfish (SCAR-MarBIN)	3968	49
MARMAP Blackfish Trap Survey (USOBIS)	7452	48
MARMAP Florida Antillean Trap Survey (USOBIS)	4569	48
MMS Ship Survey, PNW 1989 (OBIS-SEAMAP)	5790	47
Benthic marine algae in the Northern Adriatic Sea (EurOBIS)	382	47
WADFW PSAMP S1999 (OBIS-SEAMAP)	3822	46
SEFSC GoMex Oceanic 1999 (OBIS-SEAMAP)	1276	46
Shebaplankton_Falk3 (ArcOD/AOOS)	7847	46
CASES2003_2004 (ArcOD/AOOS)	4207	46
Eelgrass Community across an eutrophication gradient in New Brunswick and Prince Edward Island, Canada. (FMAP_Eutroph)	231	46
SEFSC GoMex Oceanic 1997 (OBIS-SEAMAP)	2103	45
WADFW PSAMP S1992 (OBIS-SEAMAP)	12156	45
ZooGene A DNA Sequence Database for Calanoid Copepods and Euphausiids (CoML)	114	44
SEFSC Atlantic surveys, 1998 (3) (OBIS-SEAMAP)	1031	44
SEFSC GoMex Oceanic 2001 (OBIS-SEAMAP)	825	44
ESAS Trip 9408HE (Trial) (OBIS-SEAMAP)	16131	44
Australian Institute of Marine Science - Surveys of Octocoral communities, benthic cover and environmental factors on coral reefs of Milne Bay, Papua New Guinea. (Australian Institute of Marine Science)	357	43
Copepoda collected from Fletchers Ice Island (T-3) in the Canadian Basin of the Arctic Ocean (ArcOD/AOOS)	16635	42
UK Royal Navy Marine Mammal Observations (OBIS-SEAMAP)	1408	41
SEFSC Atlantic surveys 1999 (OBIS-SEAMAP)	1068	41
Walter Herwig 1978 (FFS): SeaStars (Echinodermata, Asteroidea) (SCAR-MarBIN)	795	41
Zooplankton Bering Strait Tiglax 1991 (ArcOD/AOOS)	1271	41
Asteroids from French subantarctic islands: records from the Marion Dufresne MD03 expeditions (SCAR-MarBIN)	883	41
Ezemvelo KwaZulu-Natal Wildlife - Benthic survey on Aliwal Shoal (May 2004 - May 2005)(BAS) (AfrOBIS)	586	40
Australian Institute of Marine Science - Surveys of Octocoral communities, benthic cover and environmental factors on coral reefs of Torres Strait. (Australian Institute of Marine Science)	295	40

TWorsfold CullercoatsBay 2003 (EurOBIS)	53	39
Australian Institute of Marine Science Zooplankton community composition of Darwin Harbour, Northern Territory. (Australian Institute of Marine Science)	550	39
SOVIET ANTARCTIC EXPEDITIONS for Zooplankton (SCAR-MarBIN)	149	39
SAM Mammals (OBIS Australia)	894	38
BLM CETAP AIR Sightings (OBIS-SEAMAP)	11080	38
Meiofauna from Lynher estuary in microcosms with contaminated sediment from the Fal estuary (EurOBIS)	111	38
Atlantic Canada Conservation Data Centre (OBIS Canada)	1365	37
MV Mammals (OBIS Australia)	493	37
SEFSC Gomex Shelf 1994 (OBIS-SEAMAP)	1768	37
Zooplankton Abundance Data Sampled During the Western Bering Sea Ecological Cruise (WEBSEC) (USOBIS)	830	37
SEFSC Atlantic surveys 1992 (OBIS-SEAMAP)	1209	36
BLM CETAP OPP Sightings (OBIS-SEAMAP)	9960	36
Marine Invertebrate Diversity Initiative (OBIS Canada)	295	35
SWFSC Cetacean Sightings in the Eastern Tropical Pacific (1509) (OBIS-SEAMAP)	1147	35
SWFSC Cetacean Sightings in the Eastern Tropical Pacific (1508) (OBIS-SEAMAP)	652	35
Australian Institute of Marine Science - Surveys of Octocorals communities, benthic cover and environmental factors on coral reefs of Palau. (Australian Institute of Marine Science)	376	35
Demersal and pelagic species from the Patagonian shelf (Argentinean RON)	1733	34
Copepoda collected from the Canada Basin Arctic Ocean; Fletchers Ice Island (T-3) 1970-1972 and AIDJEX, 1975. (ArcOD/AOOS)	6999	34
SEFSC Gomex Shelf 2001 (OBIS-SEAMAP)	870	33
BLM CETAP SHIP Sightings (OBIS-SEAMAP)	3699	33
Siphonophora (Tropical and Subtropical Western South Atlantic OBIS)	1803	33
Australian Institute of Marine Science - Surveys of Octocorals in the Rowley Shoals Marine Park and the Mermaid Reef National Marine Nature Reserve, Western Australia. (Australian Institute of Marine Science)	379	33
SWFSC Cetacean Sightings in the Eastern Tropical Pacific (1468) (OBIS-SEAMAP)	1239	32
SWFSC Marine Mammal Survey of the Eastern Tropical Pacific (1165) (OBIS-SEAMAP)	565	32
SWFSC Marine Mammal Survey in the Eastern Tropical Pacific (1467) (OBIS-SEAMAP)	887	32
Marine Birds MZUSP (Tropical and Subtropical Western South Atlantic OBIS)	263	32
Historical zooplankton records from the Black Sea (EurOBIS)	65418	32
SWFSC Marine Mammal Survey of the Eastern Tropical Pacific (989) (OBIS-SEAMAP)	876	31
National Whale and Dolphin Sightings and Strandings Database (Australian Antarctic Data Centre)	2065	31
An Analysis of the Zooplankton Community Structure of the Western Beaufort Sea. WEBSEC 1971 (ArcOD/AOOS)	1247	31
Admiralty Bay Benthos Diversity Data Base (ABBED). Polychaeta. 1979-80 (SCAR-MarBIN)	192	31
Antarctic Foraminiferans (SCAR-MarBIN)	185	31
Canada Maritimes Regional Cetacean Sightings (OBIS Canada)	29784	30
SWFSC Marine Mammal Survey of the Eastern Tropical Pacific (1267) (OBIS-SEAMAP)	853	30
SWFSC Marine Mammal Survey of the Eastern Tropical Pacific (1164) (OBIS-SEAMAP)	657	30
SEFSC Caribbean Survey 1995 (OBIS-SEAMAP)	1484	30
Oceanexploration2002_vers4 (ArcOD/AOOS)	1163	30
Nematodes from the Lynher Estuary (microcosm experiments) (EurOBIS)	171	30
Biogeographic distribution of Antarctic and sub-Antarctic Cumacea (SCAR-MarBIN)	1494	30

SWFSC Marine Mammal Survey of the Eastern Tropical Pacific (1268) (OBIS-SEAMAP)	565	29
SWFSC Marine Mammal Survey of the Eastern Tropical Pacific (1081) (OBIS-SEAMAP)	724	29
SWFSC Marine Mammal Survey of the California Coast (1426) (OBIS-SEAMAP)	1013	29
Cetacean distribution in the South Atlantic and South Pacific Ocean (AR-OBIS) (Argentinean RON)	1801	29
Meiofauna of the Southern Baltic (EurOBIS)	447	29
Virginia Aquarium Marine Mammal Strandings 1988-2008 (OBIS-SEAMAP)	1555	29
Seasonal dynamics of sub-ice fauna below pack ice in the Arctic (Fram Strait) (ArcOD/AOOS)	499	29
SWFSC Cetacean Acoustic Detection and Dive Interval Studies (1601) (OBIS-SEAMAP)	602	28
Mingan Island Cetacean Study 84-07 (OBIS-SEAMAP)	4893	28
Zooplankton Guarau River (Tropical and Subtropical Western South Atlantic OBIS)	745	28
Harpacticoida species and meiofauna major taxa from Hooksiel (EurOBIS)	1266	28
Arctic Meiofauna Succession (EurOBIS)	363	28
Meiofauna from Kongsfjord (Spitsbergen Arctic) (EurOBIS)	450	28
Bermuda Atlantic Time-series Study Zooplankton Census	635	27
ZOOLOGIA_FCNO_UDEC_CL Foraminiferos_01 (ESPOBIS)	3318	27
Aerial survey of upper trophic level predators on PLatts Bank, Gulf of Maine (Gulf of Maine Census of Marine Life Program)	961	27
SWFSC Marine Mammal Survey of the Eastern Tropical Pacific (1080) (OBIS-SEAMAP)	670	27
SWFSC Marine Mammal Survey of the Eastern Tropical Pacific (1370) (OBIS-SEAMAP)	570	27
SWFSC OR, CA, WA Line-Transect Experiment (Orcawale) (1604) (OBIS-SEAMAP)	986	27
RMT Trawl catch from the 2003/04 V3 BROKE-West voyage (Australian Antarctic Data Centre)	547	27
The Baltic Expedition 1901 of the German sea fisheries association (EurOBIS)	137	27
Arctic non-copepod Zooplankton T3 Ice Island 1966-1967 (ArcOD/AOOS)	2255	27
MAR-ECO 2004 - Mammals and birds (EurOBIS)	1164	27
Seabird 2000 (EurOBIS)	24193	26
SWFSC Marine Mammal Survey of the Eastern Tropical Pacific (1369) (OBIS-SEAMAP)	667	26
Cape Hatteras 04-05 (OBIS-SEAMAP)	1259	26
ANDEEP3: SeaStars (Echinodermata, Asteroidea) (SCAR-MarBIN)	50	26
Antarctic Isopods (SCAR-MarBIN)	43	26
SWFSC Marine Mammal Survey of the Eastern Tropical Pacific (990) (OBIS-SEAMAP)	442	25
Biogeographic distribution of the Antarctic and Sub-Antarctic brachiopods (living forms) (SCAR-MarBIN)	1430	25
Bay of Puck dataset (EurOBIS)	539	24
Bahamas Marine Mammal Research Organisation Opportunistic Sightings (OBIS-SEAMAP)	2194	24
SEFSC GoMex Oceanic 1993 (W) (OBIS-SEAMAP)	836	24
Chucki_Seaooplankton1953/4 (ArcOD/AOOS)	1910	23
Zooplankton of the Eastern South Pacific, Universidad de Guayaquil, Ecuador (ESPOBIS)	337	22
MMS Marine Mammal Survey, PNW 1989-1990 (OBIS-SEAMAP)	1905	22
MMS High Altitude Survey 1980-1983 (OBIS-SEAMAP)	2229	22
Holsatia-expedition 1887 - animals collected with a dredge during the expedition (EurOBIS)	64	22
SWFSC OR, CA, WA Line-Transect Experiment (Orcawale) (1605) (OBIS-SEAMAP)	406	21
ABBBS Bird Banding records from the Australian Antarctic Territory and Heard	13628	21

Island. (Australian Antarctic Data Centre)		
Australian Institute of Marine Science - Surveys of Octocoral communities, benthic cover and environmental factors on coral reefs of Hong Kong. (Australian Institute of Marine Science)	173	21
Ross Sea Pycnogonids (SCAR-MarBIN)	104	21
SEFSC Gomex Shelf 2000 (OBIS-SEAMAP)	250	20
MMS Low Altitude Survey, SCB 1975-1978 (OBIS-SEAMAP)	1319	20
SIO_FAMIZ (ArcOD/AOOS)	455	20
Ongoing UK MarLIN Shore Thing timed search results (EurOBIS)	441	20
Marine birds and mammals of the Southern Ocean (a census for the CAML) (SCAR-MarBIN)	770	20
Icota - Pelagant (SCAR-MarBIN)	4127	20
NEFSC Aerial Survey - Summer 1998 (OBIS-SEAMAP)	704	19
NEFSC Mid-Atlantic Marine Mammal Abundance Survey 2004 (OBIS-SEAMAP)	529	19
Squid specimens from the 1991/92 V6 AAMBER2 voyage (Australian Antarctic Data Centre)	105	19
Nemertina World Checklist (SCAR-MarBIN)	530	18
NEFSC Survey 1998 1 (OBIS-SEAMAP)	505	18
NEFSC Aerial Survey - Experimental 2002 (OBIS-SEAMAP)	555	18
Zooplankton Abundance Based on Taxa and Life Stages or Size (SCAR-MarBIN)	6782	18
Zooplankton abundance and population structures assessed during April-September of 2001-2, Southern Ocean, GLOBEC (SCAR-MarBIN)	3974	18
Cetacean distribution around Mayotte Island (OBIS-SEAMAP)	434	18
UNCW Aerial Survey 1998-1999 (OBIS-SEAMAP)	368	17
NEFSC Aerial Circle-Back Abundance Survey 2004 (OBIS-SEAMAP)	758	17
UNCW Marine Mammal Sightings 1998-1999 (OBIS-SEAMAP)	329	17
NEFSC Aerial Survey - Summer 1995 (OBIS-SEAMAP)	481	17
SEFSC Southeast Cetacean Aerial Survey 1995 (OBIS-SEAMAP)	624	17
FPN-EH-AVES (Argentinean RON)	551	17
Uruguay Nearshore Zooplankton (Tropical and Subtropical Western South Atlantic OBIS)	50	17
NMML Bering Sea Cetacean Survey 2000 (OBIS-SEAMAP)	428	16
SEFSC Gomex Shelf 1998 (OBIS-SEAMAP)	112	16
Bahamas Marine Mammal Research Organisation Strandings (OBIS-SEAMAP)	89	16
Mediterranean seabird surveys 99/00/02 (OBIS-SEAMAP)	1079	16
NEFSC Survey 1998 2 (OBIS-SEAMAP)	315	16
MMS High Altitude Survey, SCB 1975-1978 (OBIS-SEAMAP)	695	16
Seabird nearshore winter survey in South-West England 1994-95 (EurOBIS)	1480	16
NEFSC 1995 pe9502 (OBIS-SEAMAP)	154	15
UK NHM Whale Strandings 1970-79 (OBIS-SEAMAP)	378	15
NEFSC North Atlantic Right Whale Sighting Survey - Fall 2008 (OBIS-SEAMAP)	514	15
NOAAs Southeast Fishery Science Center (SEFSC) Fisheries Log Book System (FLS) Commercial Pelagic Logbook Data (SEFSC_LogBook)	451827	15
Pacific Ocean Shelf Tracking (OBIS Canada)	328623	14
DFO Maritimes Research Vessel Trawl Surveys Invertebrate Observations (OBIS Canada)	14996	14
NEFSC 1995 pe9501 (OBIS-SEAMAP)	440	14
SMRU Small Cetacean Abundance NS 1994 (OBIS-SEAMAP)	2376	14
NEFSC 1999 aj9902 (OBIS-SEAMAP)	1091	14
Hatteras Eddy Cruise 2004 (OBIS-SEAMAP)	230	14
SEFSC Mid-Atlantic Tursiops Survey, 1995 3 (OBIS-SEAMAP)	1000	13
UNCW Right Whale Aerial Survey 05-06 (OBIS-SEAMAP)	1575	13
SEFSC Southeast Cetacean Aerial Survey 1992 (OBIS-SEAMAP)	871	13
Bahamas Marine Mammal Research Organisation On-transect Sightings (OBIS-SEAMAP)	185	13
Alnitak Cetaceans and sea turtles surveys off Southern Spain (OBIS-SEAMAP)	4010	13
NEFSC Harbor Porpoise 1991 (OBIS-SEAMAP)	782	13

NEFSC 1992 aj9201 (OBIS-SEAMAP)	1260	13
NEFSC Deepwater Marine Mammal 2002 (OBIS-SEAMAP)	108	13
Biopearl expedition: SeaStars (Echinodermata, Asteroidea) (SCAR-MarBIN)	24	13
2008 UNCW Right Whale Aerial Surveys (OBIS-SEAMAP)	2011	13
Marine and Coastal Management - Copepod Surveys (AfrOBIS)	91705	12
APIS - Antarctic Pack Ice Seals 1994-1999, plus historical data from the 1980's (Australian Antarctic Data Centre)	9271	12
ARGOS Satellite Tracking of animals (Australian Antarctic Data Centre)	213488	12
Cetacean Sightings Survey and Southern Ocean cetacean program (Australian Antarctic Data Centre)	266	12
Inventory of Antarctic seabird breeding sites (Australian Antarctic Data Centre)	2787	12
Sargasso 2004 - Seabirds (OBIS-SEAMAP)	168	12
UNCW Marine Mammal Sightings 2001 (OBIS-SEAMAP)	514	12
NEFSC Survey 1997 (OBIS-SEAMAP)	60	12
UNCW Marine Mammal Aerial Surveys 2006-2007 (OBIS-SEAMAP)	2269	11
NMML Small Cetacean Aerial Survey 1997 (OBIS-SEAMAP)	602	11
NEFSC 1995 AJ9501 (Part II) (OBIS-SEAMAP)	1419	11
Ice Algae Barents Sea (ArcOD/AOOS)	36	11
Antarctic Pycnogonids II (SCAR-MarBIN)	115	11
NMML Small Cetacean Aerial Survey 1999 (OBIS-SEAMAP)	434	10
SEFSC Mid-Atlantic Tursiops Survey, 1995 2 (OBIS-SEAMAP)	827	10
Sargasso 2005 - cetacean sightings (OBIS-SEAMAP)	85	10
Cetacean survey in Balabac Strait, Philippines (OBIS-SEAMAP)	32	10
Whale log - observations from ANARE voyages (Australian Antarctic Data Centre)	113	10
Ross Coral Mapping Project - NBN South West Pilot Project Case Studies (EurOBIS)	32	10
Marine gastropod distribution from patagonian shallow waters (Argentinean RON)	63	10
Admiralty Bay Benthos Diversity Data Base (ABBED). Cumacea. (SCAR-MarBIN)	182	10
NMML Small Cetacean Aerial Survey 1998 (OBIS-SEAMAP)	305	9
NMML Bering Sea Cetacean Survey 1999 (OBIS-SEAMAP)	339	9
NEFSC 1995 AJ9501 (Part I) (OBIS-SEAMAP)	150	9
UNCW Marine Mammal Sightings 2002 (OBIS-SEAMAP)	835	9
Hydroids of the BANZARE Antarctic expeditions 1929 - 1931 (Australian Antarctic Data Centre)	29	9
The Sea Ice Fauna of Frobisher Bay, Arctic Canada 1981 and 1982 (ArcOD/AOOS)	289	9
QM Crust (OBIS Australia)	68	8
SEFSC Mid-Atlantic Tursiops Survey, 1995 1 (OBIS-SEAMAP)	785	8
Whale Observations from the British, Australian and New Zealand Antarctic Reaserch Expedition (BANZARE) voyages 1929-30 and 1930-31 (Australian Antarctic Data Centre)	144	8
Cetacean diversity, distribution, and abundance in northern Veracruz, Mexico (OBIS-SEAMAP)	96	8
Visual and genetic surveys for odontocete cetaceans in American Samoa 2003-06 (OBIS-SEAMAP)	59	8
Antarctic Marine Bacteria from Denmark University (SCAR-MarBIN)	51	8
Resolute Passage Copepod Distribution (OBIS Canada)	3428	7
SAM Herpetology (OBIS Australia)	31	7
Bahamas Marine Mammal Research Organisation Aerial Survey (OBIS-SEAMAP)	17	7
Nest census, Windmill Islands 2002/03 (Australian Antarctic Data Centre)	5056	7
Distribution data of Arctic species of genus Microporella and Pseudoflustra gathered from museum collections (ArcOD/AOOS)	113	7
Whale catches in the Southern Ocean (Australian Antarctic Data Centre)	7122	6
NMML Harbor Porpoise Vessel Survey, SE Alaska, Summer 1991 (OBIS-SEAMAP)	445	6

NMML Killer Whale Vessel Survey, Alaska Peninsula, 1992 (OBIS-SEAMAP)	36	6
NMML Harbor Porpoise Vessel Survey, SE Alaska, Spring 1991 (OBIS-SEAMAP)	382	6
NEFSC Survey 1991 (OBIS-SEAMAP)	80	6
NMML Harbor Porpoise Aerial Survey, Bristol Bay, Replicate 1, 1991 (OBIS-SEAMAP)	38	6
NMML Killer Whale Vessel, Kodiak Island, 1992 (OBIS-SEAMAP)	408	6
NMML Harbor Porpoise Aerial Survey, Kodiak Island, Replicate 3, 1992 (OBIS-SEAMAP)	90	6
NMML Harbor Porpoise Vessel, SE Alaska, Summer 1993 (OBIS-SEAMAP)	614	6
NMML Harbor porpoise Aerial Survey, Kodiak Island, Replicate 1, 1992 (OBIS-SEAMAP)	73	6
NMML Killer Whale Vessel, Alaska Peninsula 1993 (OBIS-SEAMAP)	88	6
NMML Harbor Porpoise Vessel, SE Alaska, Spring 1993 (OBIS-SEAMAP)	669	6
NMML Killer Whale Vessel, Kodiak Island, 1993 (OBIS-SEAMAP)	516	6
Antarctic Euphausiacea occurence data from "German Antarctic Marine Living Resources" (GAMLR) Expeditions (SCAR-MarBIN)	3678	6
Antarctic and sub-Antarctic Lophogastrida occurrences (SCAR-MarBIN)	43	6
Admiralty Bay Benthos Diversity Data Base (ABBED). Tanaidacea. (SCAR-MarBIN)	187	6
Southern Ocean oligochaete occurrence data - a literature-based compilation (SCAR-MarBIN)	30	6
Marine Turtles (EurOBIS)	2287	5
Brachiopoda from sampling campaigns in the French part of the Mediterranean during the 1970-1990s (EurOBIS)	468	5
NMML Harbor Porpoise Vessel, SE Alaska, Fall 1993 (OBIS-SEAMAP)	289	5
NMML Harbor Porpoise Aerial Survey, SE Alaska, Replicate 2, 1993 (OBIS-SEAMAP)	126	5
NMML Harbor Porpoise Vessel, SE Alaska, Summer 1992 (OBIS-SEAMAP)	485	5
NMML Killer Whale Vessel, Bering Sea, 1992 (OBIS-SEAMAP)	184	5
NMML Harbor Porpoise Vessel, SE Alaska, Spring 1992 (OBIS-SEAMAP)	530	5
NMML Harbor Porpoise Vessel, SE Alaska, Fall 1992 (OBIS-SEAMAP)	308	5
UNCW Aerial Surveys for monitoring of proposed Oslow Bay USWTR site - Left side - (OBIS-SEAMAP)	66	5
NMML Harbor Porpoise Aerial Survey, SE Alaska, Replicate 3, 1993 (OBIS-SEAMAP)	165	5
globec/soglobec/process/krill (SCAR-MarBIN)	6012	5
Cetaceans in the Southern Indian Ocean 2004 (OBIS-SEAMAP)	13	5
Antarctic Euphausiacea occurence data from Polish FIBEX expeditions (SCAR-MarBIN)	86	5
Ice Zooplankton Beaufort Sea (ArcOD/AOOS)	32	5
Taxonomy and zoogeography boundaries of pelagic ostracods in Svalbard waters, 2001-2006 (ArcOD/AOOS)	3619	5
NMML Harbor Porpoise Aerial Survey, Kodiak Island, Replicate 2, 1992 (OBIS-SEAMAP)	77	4
NMML Harbor Porpoise Vessel Survey, SE Alaska, Fall 1991 (OBIS-SEAMAP)	112	4
Bahamas Marine Mammal Research Organisation Turtles (OBIS-SEAMAP)	101	4
DUML Vessel-Based Surveys for monitoring of proposed Oslow Bay USWTR site (OBIS-SEAMAP)	35	4
UNCW Aerial Surveys for monitoring of proposed Oslow Bay USWTR site - Right side - (OBIS-SEAMAP)	75	4
NMML Harbor Porpoise Aerial Survey, SE Alaska, Replicate 1, 1993 (OBIS-SEAMAP)	143	4
NMML Harbor Porpoise Aerial Survey, Alaska Peninsula, Replicate 1, 1992 (OBIS-SEAMAP)	20	4
NMML Harbor Porpoise Aerial Survey, Bristol Bay, Replicate 3, 1991 (OBIS-SEAMAP)	13	4
NMML Harbor Porpoise Aerial Survey, Cook Inlet, 1991 (OBIS-SEAMAP)	11	4
Ice Amphipods Svalbard, 2000 (ArcOD/AOOS)	7	4

Biology and Ecology of Cryopelagic Amphipods from Arctic Sea Ice Collected near Franz Josef Land in the summer of 1994 (ArcOD/AOOS)	11	4
Antarctic Marine Amoebae (SCAR-MarBIN)	7	4
NT Ichthyology (OBIS Australia)	6	3
NMML Harbor Porpoise Aerial Survey, Alaska Peninsula, Replicate 2, 1992 (OBIS-SEAMAP)	41	3
Harbour porpoises, white-beaked dolphins and minke whales in North Sea - Land surveys - (OBIS-SEAMAP)	103	3
Harbour porpoises, white-beaked dolphins and minke whales in North Sea - Vessel surveys - (OBIS-SEAMAP)	71	3
NMML Harbor Porpoise Aerial Survey, Bristol Bay, Replicate 2, 1991 (OBIS-SEAMAP)	10	3
Pacific Turtle Tracks: Grupo Tortuguero (OBIS-SEAMAP)	2250	3
Locations of seals in Patagonian Large Marine Ecosystem (OBIS South America, AR-OBIS, Sub-node) (Argentinean RON)	4060	3
Antarctic Euphausiacea occurence data from Norwegian Antarctic Research Expedition 1976-77 (SCAR-MarBIN)	22	3
Abundance and distribution of cetaceans in the state of Aragua, Venezuela (OBIS-SEAMAP)	29	3
Current status of cetaceans in Aragua, Venezuela (OBIS-SEAMAP)	32	3
Under-ice Amphipods in the Greenland Sea and Fram Strait (Arctic): Environmental Controls and Seasonal Patterns Below the Pack Ice (ArcOD/AOOS)	66	3
Penguins of Antarctica (SCAR-MarBIN)	8	3
Virgin Islands National Park Coral Transplant Study (USOBIS)	908	3
SAM Marine Invertebrates (OBIS Australia)	54	2
Kelp rafts in the Southern Ocean (Australian Antarctic Data Centre)	122	2
Cape Cod Sea Turtle Release 2007 (OBIS-SEAMAP)	245	2
NMML Killer Whale Vessel, Bering Sea, 1993 (OBIS-SEAMAP)	57	2
USAKA Turtle Release Program (OBIS-SEAMAP)	30	2
Virginia Aquarium Stranding Response Program (OBIS-SEAMAP)	1747	2
Cayman Islands 2003: Loggerhead & Green Turtles (OBIS-SEAMAP)	1584	2
Cayman Islands 2004: Loggerhead & Green Turtles (OBIS-SEAMAP)	561	2
NMFS Turtle Tracking (OBIS-SEAMAP)	8012	2
Tern Island Albatrosses - 1998 (OBIS-SEAMAP)	3505	2
ME harbor and gray seals time series (OBIS-SEAMAP)	6973	2
Casey Key Loggerheads-2007 (OBIS-SEAMAP)	3522	2
Duke North Atlantic Turtle Tracking (OBIS-SEAMAP)	10136	2
Harbour seals in Republic of Ireland in Aug 2003 (OBIS-SEAMAP)	435	2
Tern Island Albatrosses - 1999 (OBIS-SEAMAP)	4635	2
Mote Marine Laboratory - Sea Turtle Rehabilitation Hospital (OBIS-SEAMAP)	1247	2
Antarctic Euphausiacea occurrence data from Norwegian Antarctic Research Expedition 1979 (SCAR-MarBIN)	47	2
Antarctic deep-sea meiofauna (EurOBIS)	1476	2
Snow Petrel nest census, Mawson region 2004/05 (Australian Antarctic Data Centre)	5588	2
Ice Amphipods Canada Basin (ArcOD/AOOS)	10	2
Sea Ice Nematodes (ArcOD/AOOS)	32	2
National Marine Life Center: Sea Turtle Releases (OBIS-SEAMAP)	192	2
Angola Sea Turtle Tracking Project (OBIS-SEAMAP)	192	2
Turks and Caicos Islands Turtle Project 2009 (OBIS-SEAMAP)	110	2
Bali turtles (OBIS-SEAMAP)	180	2
Population status of small cetaceans off Aragua, Central Coast of Venezuela 2009 (OBIS-SEAMAP)	39	2
TOPP Albatrosses 2002-06 (OBIS-SEAMAP)	33789	2
NEFSC Marine Mammal Abundance Cruise 2004 Passive Acoustic Monitoring - Rainbow Click Detections (OBIS-SEAMAP)	1210	2
Turks and Caicos Islands Turtle Project 2009: Green & Hawksbill Turtles (OBIS-SEAMAP)	568	2

Casey Key Loggerheads - 2009 (OBIS-SEAMAP)	6246	2
Rough-toothed dolphins and false killer whales in Hawai'i (OBIS-SEAMAP)	93	2
Elephant Seal Sightings, Heard Island (Australian Antarctic Data Centre)	1794	1
Weddell Seal census, Vestfold Hills, Antarctica (Australian Antarctic Data Centre)	4553	1
Weddell Seal Sightings, Vestfold Hills, Antarctica (Australian Antarctic Data Centre)	20992	1
MV Entomology (OBIS Australia)	9	1
NT Mollusca (OBIS Australia)	1	1
Marine and Coastal Management - Seal Surveys (AfrOBIS)	2440	1
Elephant Seal Sightings, Macquarie Island (Australian Antarctic Data Centre)	221619	1
Orca observations from the shores of Macquarie Island (Australian Antarctic Data Centre)	248	1
Macquarie Island Fur Seal Database (Australian Antarctic Data Centre)	5771	1
Antarctic Fur Seal Populations on Heard Island Summer 1987-1988 (Australian Antarctic Data Centre)	462	1
CCAMLR historic KRILL (SCAR-MarBIN)	63364	1
Interannual variability of Alexandrium fundyense abundance in the Gulf of Maine	3409	1
IPHC Opportunistic Albatross Obs 1998-2002 (OBIS-SEAMAP)	141	1
Pacific Sea Turtle Tracking - Aquarium of the Pacific (OBIS-SEAMAP)	27	1
Russian Barnacle Geese (OBIS-SEAMAP)	202	1
Sperm whales off Peru during IMARPE surveys (1995-2002) (OBIS-SEAMAP)	38	1
Killer whales off Peru during IMARPE surveys (1995-2003) (OBIS-SEAMAP)	14	1
Allied Finback Whale Catalogue (OBIS-SEAMAP)	648	1
The Dolphin Project (OBIS-SEAMAP)	5665	1
Sargasso sperm whales 2004 (OBIS-SEAMAP)	11	1
Casey Key Loggerheads - 2005-2006 (OBIS-SEAMAP)	1632	1
Sangalaki Green Turtles Tracking (OBIS-SEAMAP)	169	1
Vietnam Sea Turtle Tracking Project (OBIS-SEAMAP)	400	1
YoNAH Encounter (OBIS-SEAMAP)	4215	1
Waved Albatross Tracking (OBIS-SEAMAP)	457	1
Cabo Verde (Proyecto Aegina): male and Hortensia loggerheads (OBIS-SEAMAP)	2448	1
Cape Verde (Cabo Verde) 2005: Loggerhead Turtles (OBIS-SEAMAP)	404	1
Bald Head Island 2004: Loggerhead Turtles (OBIS-SEAMAP)	3214	1
East Pacific Sea Turtle Tracking 1996-1997 (OBIS-SEAMAP)	394	1
Duke Albatross 1997-1999 (OBIS-SEAMAP)	543	1
Cascadia Research Blue Whale Photo IDs for US West Coast, 1972-2004 (OBIS-SEAMAP)	6535	1
Islas Canarias (Proyecto Aegina): juvenile loggerheads (OBIS-SEAMAP)	3927	1
Migratory patterns of Yucatan Peninsula hawksbills (OBIS-SEAMAP)	1906	1
SMRU Grey Seal UK 1991-1993 (OBIS-SEAMAP)	9454	1
Wood Stork Tracking (OBIS-SEAMAP)	12798	1
Bald Head Island 2003: Loggerhead Turtles (OBIS-SEAMAP)	1944	1
Cape Verde (Cabo Verde) 2004: Loggerhead Turtles (OBIS-SEAMAP)	3139	1
Allied Humpback Whale Catalogue, 1976 - 2003 (OBIS-SEAMAP)	3928	1
Duke Harbor Porpoise Tracking (OBIS-SEAMAP)	5938	1
SEFSC Dolphin Photo ID (OBIS-SEAMAP)	2443	1
Piai Island Green Sea Turtle Tracking (OBIS-SEAMAP)	224	1
MMS O2 1982-1984 (OBIS-SEAMAP)	1734	1
Newport Aquarium 2004: Loggerhead Turtle (OBIS-SEAMAP)	741	1
Marion Wanderers (OBIS-SEAMAP)	1486	1
SMRU Elephant Seal Pup Tracking 1995-1996 (OBIS-SEAMAP)	7245	1
NMML Harbor Porpoise Aerial Survey, SE Alaska, 1991 (OBIS-SEAMAP)	3	1
NPPSD Short-tailed Albatross Sightings (OBIS-SEAMAP)	1321	1
Baltic Porpoise Sightings 01-02 (OBIS-SEAMAP)	55	1
Palau Marine Turtle Conservation and Monitoring Program (OBIS-SEAMAP)	29	1
Bald Head Island 2005: Loggerhead Turtles (OBIS-SEAMAP)	4428	1
Population viabilty analysis of the Perth metropolitan population of Little Penguins (OBIS-SEAMAP)	1500	1

Kittlitzs_murrelet (ArcOD/AOOS)	21	1
BenthosBarentsSeaPolarstern1991 (ArcOD/AOOS)	11	1
Distribution ashore and breeding places of southern elephant seals (Argentinean RON)	588	1
Southern right whales distribution in Baha Nueva, Puerto Madryn, Argentina (Argentinean RON)	725	1
Antarctic Krill occurrence data from BAS expeditions (SCAR-MarBIN)	346	1
Antarctic Krill occurrence data from Discovery expeditions (SCAR-MarBIN)	667	1
Antarctic Krill occurrence data from Spanish RV "Fruela" (SCAR-MarBIN)	25	1
Tagged Seal Location Data from SOGLOBEC (SCAR-MarBIN)	41676	1
Antarctic Krill occurrence data from Japanese expeditions (SCAR-MarBIN)	19	1
Antarctic Krill occurrence data from US AMLR "US Antarctic Marine Living Resources" Program (SCAR-MarBIN)	1268	1
Antarctic Krill occurrence data from Ukraine YUGNIRO Institute (SCAR-MarBIN)	592	1
Whale Watch Azores Bryde's whale 2004 (OBIS-SEAMAP)	19	1
Leatherback Tracking in South Africa (OBIS-SEAMAP)	3635	1
Juvenile Green Sea Turtles from Argentina (OBIS-SEAMAP)	162	1
Satellite Tracking of Olive Ridley Turtles at Jamursba-medi, West Papua - Indonesia (OBIS-SEAMAP)	483	1
Study of young rehabilitated harbour seal in the north of France (OBIS-SEAMAP)	1237	1
Netherlands Antilles Turtle Tracking 2007 (OBIS-SEAMAP)	442	1
Casey Key Loggerheads-2008 (OBIS-SEAMAP)	3403	1
Satellite tracking of nesting loggerhead tutles at Ningaloo Marine Park, Western Australia (OBIS-SEAMAP)	989	1
Satellite Tracking of Hawksbill Turtle in West Sumbawa, Indonesia (OBIS-SEAMAP)	165	1
South Australia Sea Lions as Ocean Observers (OBIS-SEAMAP)	210	1
Green Sea Turtles Tracking in Sukamade, Meru Betiri National Park-East Java (OBIS-SEAMAP)	789	1
Understanding the effects of climate change on Caribbean hawksbill turtles: satellite tracking hawksbill migrations (OBIS-SEAMAP)	36	1
New England Aquarium Harbor Porpoise Tracking (OBIS-SEAMAP)	179	1
Antarctic Krill occurrence data from Australian expeditions (SCAR-MarBIN)	234	1
Antarctic Euphausiacea occurrence data from ITALICA 2000 Expedition (SCAR-MarBIN)	44	1
Leopard Seal census, Heard Island 1987/88 (Australian Antarctic Data Centre)	534	1
Heard Island Shag *Phalacrocorax nivalis* census, Heard Island 1992 (Australian Antarctic Data Centre)	91	1
Subantarctic Fur seals at Heard Island, 1987/88 (Australian Antarctic Data Centre)	11	1
Historical quantitative benthos grab samples from the Southern Baltic Sea - Polish data (EurOBIS)	8039	1
Bunger Hills, 1999/2000 survey - nest sites of snow petrels *Pagodroma nivea* (Australian Antarctic Data Centre)	140	1
ECOCEAN Whale Shark Photo-identification Library (ECOCEAN_WhaleSharks)	8417	1
TOPP northern elephant seal (*Mirounga angustirostris*) ARGOS satellite tracking (TOPP)	1445	1
Royal penguin *Eudyptes schlegeli* census and observations at North Head, Macquarie Island 1952/53 (Australian Antarctic Data Centre)	156	1
Southern Giant Petrel census data within the Australian Antarctic Territory. (Australian Antarctic Data Centre)	121	1
King penguin census data, Gadget Gully, Macquarie Island (1993-2008) (Australian Antarctic Data Centre)	607	1
Ivory Gulls from Northern Greenland (OBIS-SEAMAP)	5215	1
Bottlenose dolphin abundance in coastal Moreton Bay 2000 (OBIS-SEAMAP)	48	1
Gabon Olive Ridley Project (OBIS-SEAMAP)	1709	1
Tracking on Magnifying Olive ridley Journey in Kaironi beach, Papua-Indonesia (OBIS-SEAMAP)	697	1
Canary Islands - OAG (OBIS-SEAMAP)	1956	1

North Carolina Aquarium at Pine Knoll Shores Sea Turtle Awareness (OBIS-SEAMAP)	1285	1
Crossing the tide (OBIS-SEAMAP)	585	1
Green turtle tracking in the northern Great Barrier Reef (OBIS-SEAMAP)	506	1
Florida Manatee Mortality 1974-2008 (OBIS-SEAMAP)	6736	1
Nesting loggerheads on the Calabrian coast (Italy) (OBIS-SEAMAP)	31	1
Satellite telemetry of King Eiders from northern Alaska 2002-2009 (OBIS-SEAMAP)	11671	1
Snow Petrel census, Reeve Hill, Windmill Island, East Antarctica (1984-2003) (Australian Antarctic Data Centre)	6697	1
Royal penguin *Eudyptes schlegeli* census, Macquarie Island, 1984 (Australian Antarctic Data Centre)	56	1
Killer whales occurrences in Venezuelan waters 1982-2008 (OBIS-SEAMAP)	18	1
Tracking on Green sea turtle in South Misol, Raja Ampat-Papua, Indonesia (OBIS-SEAMAP)	369	1
First satellite tracking of sea turtles in Albania (OBIS-SEAMAP)	67	1
LMMSTRP-Kemp's Ridley 1 (OBIS-SEAMAP)	49	1
Bowen Turtle Collaboration: Girringun, Giru Dala, Gudjuda, GHD, DERM , NQBP, DEWHA (OBIS-SEAMAP)	27	1
Gabon Olive Ridley Project (OBIS-SEAMAP)	5983	1
Migration and foraging ecology of Greater Shearwater (OBIS-SEAMAP)	782	1
TARTACare Calabria: monitoring and conservation of the loggerhead turtle nesting activity along the Ionian coast of Calabria (Southern Italy) (OBIS-SEAMAP)	287	1
Baltic Porpoise Acoustic Surveys 01-02 (OBIS-SEAMAP)	462	1
Sea ice meiofauna abundance in coastal fast ice off Barrow, Alaska, with a focus on *Scolelepis squamata* (Polychaeta), 2005-2006 (ArcOD/AOOS)	695	1
Antarctic fish *Gobionotothen gibberifrons* (SCAR-MarBIN)	162	1
Antarctic Krill *Euphausia superba* (SCAR-MarBIN)	422	1
Antarctic *Parborlasia corrugatus* (SCAR-MarBIN)	102	1
Mitochondrial lineages in the Antarctic sea slug *Doris kerguelenensis* (SCAR-MarBIN)	276	1
In situ observations of *Stygiomedusa gigantea* (SCAR-MarBIN)	109	1
Eastern Canada Benthic Macro Fauna (OBIS Canada)	5650	0
SeaIceMeiofaunaCanadaBasin2002 (ArcOD/AOOS)	40	0
Bacteria and chlorophyll-a water column observations (surface to ~150m), April-August 2001, Continental Margin Western Antarctic Peninsula, GLOBEC (SCAR-MarBIN)	290	0
Macrozooplankton concentrations estimated from MOCNESS tow samples, Continental Margin Western Antarctic Peninsula, GLOBEC (SCAR-MarBIN)	1150	0
Historical quantitative benthos grab samples from the Southern Baltic Sea - German data (EurOBIS)	7547	0
Meiobenthic data Manuela (EurOBIS)	742	0
Meiofauna of the North Adriatic Sea (EurOBIS)	325	0
Size of Atlantic nematodes (EurOBIS)	950	0
NEFSC Marine Mammal Abundance Cruise 2004 Passive Acoustic Monitoring - Porpoise Detections (OBIS-SEAMAP)	912	0
The Deepwater Program: Northern Gulf of Mexico Continental Slope Habitat and Benthic Ecology - DgoMB: Macros	10336	0
The Deepwater Program: Northern Gulf of Mexico Continental Slope Habitat and Benthic Ecology - DgoMB: Meios	13416	0
Nematodes from the Goban Spur (OMEX) - 1994 (EurOBIS)	3720	0
Length/Width and biomass of nematodes from sandbanks on the Belgian Continental Shelf (EurOBIS)	3529	0
Admiralty Bay Benthos Diversity Data Base (ABBED). Pycnogonida. (SCAR-MarBIN)	67	0
Bacteria from Penguin Guano, Antarctica (SCAR-MarBIN)	70	0

Supplementary Table 6. References to the datasets used in the present study where available from the OBIS metadata and as recorded therein. In some cases the data was associated with a publication in a science journal or other report. Many datasets did not provide a citation in this format. Only reasonably complete citations are listed here.

A. Atkinson, V. Siegel , E. Pakhomov, P. Rothery (2004): Long-term decline in krill stock and increase in salps within the Southern Ocean. Nature 432: 100-103

AIMS - Baited Remote Underwater Video Stations (BRUVS): M Cappo, P Speare.

Alfred Wegener Institut fr Polar- und Meeresforschung; Senckenbergische Naturforschende Gesellschaft. - Germany. Macrozoobenthos data from the southeastern North Sea in 2000. http://www.vliz.be/vmdcdata/nsbp.

Allen D., Beckett B., Brophy J., Costello M.J., Emblow C., Maciejewska B., McCrea M., Nash R., Penk M. & Tierney A. Marine species recorded in Ireland during field suveys by EcoServe, Ecological Consultancy Services Ltd. Available online at http://www.marbef.org/data/eurobis.php.

Allison L. Schmidt. (2007). Eelgrass Community across an eutrophication gradient in New Brunswick and Prince Edward Island, Canada. Dalhousie University.

Anderson, D.J., Huyvaert, K.P., Wood, D.R., Gillikin, C.L., Frost, B.J., Mouritsen, H. 2003. At-sea distribution of waved albatrosses and the Galapagos Marine Reserve. Biological Conservation: 110 (3): 367-373

Antoniadou C. (1998). Macro- and megafauna from the North Aegean Sea from 1997-1998. Aristotle University of Thessaloniki, Department of Biology, Laboratory of Zoology, Greece.

Arturo Serrano. 2009. Aquatic Vertebrates of Veracruz 2009. Cetacean Diversity, Distribution, and Abundance in Northern Veracruz, Mexico. Aquatic Mammals. 35(1), 12-18

Arujo R., Brbara I, Tibaldo M., Berecibar E., Daz Tapia P., Pereira R., Santos R. & Sousa-Pinto I. Checklist of benthic marine algae and cyanobacteria of northern Portugal. Centre of Marine and Environmental Research; Interdisciplinary Centre for Marine and Environmental Research (Porto) (Ciimar).

Atlas de la distribucin reproductiva de aves marinas en el Litoral Patagnico Argentino. 1998. Yorio P., Frere E., Gandini P. y Harris G. Editors. Fundacin Patagonia Natural - Wildlife Conservation Society. Plan de manejo integrado de la zona costera Patagonica. 221 pp. El Hornero (2005). mero Especial Ecologa y conservacin de aves marinas.

Austen, M. & McEvoy, A. (1993). Experimental effects of TBT on meiobenthic communities. Plymouth Marine Laboratory (PML), UK.

Austen, M. & McEvoy, A. (1993). Nematodes from the Exe estuary (UK): microcosm experiments. Plymouth Marine Laboratory (PML), UK.

Austen, M. & McEvoy, A. (1993). Nematodes from the Lynher estuary (UK): microcosm experiments. Plymouth Marine Laboratory (PML), UK.

Austen, M. (1995). Nematodes of Solbergstrand, Norway (in presence and absence of *Brissopsis*). Plymouth Marine Laboratory (PML), UK.

Baird, R.W., A.M. Gorgone, D.J. McSweeney, D.L. Webster, D.R. Salden, M.H. Deakos, A.D. Ligon, G.S. Schorr, J. Barlow and S.D. Mahaffy. 2008. False killer whales (*Pseudorca crassidens*) around the main Hawaiian Islands: long-term site fidelity, inter-island movements, and association patterns. Marine Mammal Science. 24:591-612</a

Baird, R.W., D.L. Webster, S.D. Mahaffy, D.J. McSweeney, G.S. Schorr and A.D. Ligon. 2008. Site fidelity and association patterns in a deep-water dolphin: rough-toothed dolphins (Steno bredanensis) in the Hawaiian Archipelago. Marine Mammal Science. 24:535-553</a

Baker,A.J., Pereira,S.L., Haddrath,O.P. and Edge,K.A. Multiple gene evidence for expansion of extant penguins out of Antarctica due to global cooling. Proc. Biol. Sci. 273 (1582), 11-17 (2006)

Barlow, Jay, Karin A. Forney, A.V. Saunder, J. Urban-Ramirez. 1997. A Report of Cetacean Acoustic Detection and Dive Interval Studies (Caddis) Conducted in the Southern Gulf of California, 1995 NOAA-TM-NMFS-SWFSC-250:

Bernardes, R. A.; Figueiredo, J. L.; Rodrigues, A. R.; Fischer, L. G.; Vooren, C. M.; Haimovic, M.; Rossi-Wongtschowski, C. L. B. 2005. Peixes da Zona Econmica Exclusiva da Regiúo Sudeste-Sul do Brasil: levantamento com aramadilhas, pargueiras e rede de arrasto de fundo. Súo Paulo: Editora da Universidade de Súo Paulo. 295p.

Bernardes, R. A.; Rossi-Wongtschowski, C. L. D. B.; Wahrlich, R.; Vieira, R. C.; Santos, A. P.; Rodrigues, A. R. 2005. Prospecúo pesqueira de recursos demersais com aramadilhas e pargueiras na Zona Econmica Exclusiva da Regio Sudeste-Sul do Brasil. Súo Paulo: Instituto Oceanogífico da USP (Srie Documentos Revizee: Score Sul). 112 p.

Bertrand RICHER DE FORGES (IRD) & Philippe BOUCHET (MNHN). 1998. Benthic species from the tropical Pacific. IRD-Noumea

Bisschop, G. & Vincx, M. (1976). Nematode fauna of the North Sea near the Westerschelde Estuary. Ghent University, Department of Biology, Marine Biology Section (MARBIOL), Belgium.

Bluhm, B.A., MacDonald IR, Debenham C, Iken K. (2004) Macro and Megabenthic Communities in the High Arctic Canada Basin: Initial Findings. Polar Biology, 28: 218-231

Bob , R.H., K.J. Kuletz and D.A. Nigro. (1999) UAF - Kittlitz's Murrelet Known Nest Locations in Alaska (GIS layer)

Bodil Bluhm. (1998) Distribution, standing stock, growth, mortality and production of *Strongylocentrotus pallidus* (sea urchin) in the northern Barents Sea. Polar Biology, 20:325-334

Bolaâos, J. Jaime, Marcos A. Campo Z. and M. Gonzâílez-Fernâíndez. 1998. Determinaciân del estado actual de los cetâíceos de las costas del estado Aragua: Resultados de la etapa I. IT/MARNR/386, Serie Informes Tâ®cnicos, Ministerio del Ambiente, Caracas, Venezuela. 41</a

Bolaâos-Jimâ®nez, Jaime and Villarroel-Marâ¡n, Auristela. 2009. Population status of dolphin populations off the State of Aragua, Central Coast of Venezuela, Fall 2009. Final report, project RSG 08.07.07 funded by the Rufford Foundation, unpublished

Bolanos-J, Jaime and M. Campo. 1998. Aspects of the ecology and behavior of cetacean populations in the State of Aragua, Central coast of Venezuela. Abstracts Book, the World Marine Mammal Science Conference, Montecarlo, Monaco, January 1998. 17

Boltovskoy, D. 1987. Sedimentary record of radiolarian biogeography in the equatorial to Antarctic western Pacific Ocean. Micropaleontology, 33(3):267-281.

Bonecker, A. C. T.; Castro, M. S. (eds.) 2006. Atlas de larvas de peixes da regiúo central da Zona Econmica Exclusiva brasileira. Rio de Janeiro: Museu Nacional, 2006. (Srie Livros; 19) 216 p.

Bonecker, S.L.C. (ed.) 2006. Atlas de zooplóncton da regiúo central da Zona Econmica Exclusiva brasileira. Rio de Janeiro: Museu Nacional, 2006. (Srie Livros; 21) 234 p.

Bonne, W. (1997). Copepods from the Middelkerkebank (North Sea). Ghent University, Department of Biology, Marine Biology Section (MARBIOL), Belgium.

Bonne, W. (1999). Bentho-pelagic coupling in the North Sea Copepoda. Ghent University, Department of Biology, Marine Biology Section (MARBIOL), Belgium.

Bonnell, M. L, C. E. Bowlby, and G. A. Green. 1991. Pinniped Distribution and Abundance Off Oregon and Washington, 1989-1990. Final Report prepared by Ebasco Environmental, Bellevue, WA, and Ecological Consulting, Inc. , Portland, OR, for the Minerals Management Service, Pacific OCS Region. OCS Study MMS: 91-0093. 60 pp

Bonnell, M. L., B. J. Le Boeuf, M. O. Pierson, D. H. Dettman, and G. D. Farrens. 1981. Pinnipeds of the Southern California Bight. Part I of Investigator's Reports, Summary of Marine Mammal and Seabird Surveys of the Southern California Bight Area, 1975-1978. Final Report prepared by the University of California, Santa Cruz, for the Bureau of Land Management, Contract No. AA550-CT7-36. National Technical Information Service, Springfield, Virginia: NTIS # PB-81-248-171. 535 pp

Boutillier, JA, Pacific Shrimp Trawl Survey, OBIS Canada Digital Collections,2007, Bedford Institute of Oceanography, Dartmouth, Nova Scotia, Canada, OBIS Canada, 1, Digital

Briggs, K. T., D. G. Ainley, D. R. Carlson, D. B. Lewis, W. B. Tyler, L. B. Spear, and L. A. Ferris. 1987. Final Report: California Seabird Ecology Study. Prepared by the Institute of Marine Sciences, University of California, Santa Cruz, for the Pacific OCS Region, Minerals Management Service, Contract No. 14-12-001-30183vii and 153 pp

Briggs, K. T., D. H. Varoujean, W. W. Williams, R. Glenn Ford, M. L. Bonnell, and J. L. Casey. 1991. Seabirds of the Oregon and Washington OCS, 1989-1990. Final Report prepared by Ebasco Environmental, Bellevue, WA, and Ecological Consulting, Inc. , Portland, OR, for the Minerals Management Service, Pacific OCS Region. OCS Study MMS: 91-0093. 164 pp

Briggs, K. T., E. W. Chu, D. B. Lewis, W. B. Tyler, R. L. Pitman, and G. L. Hunt. 1981. istribution, Numbers, and Seasonal Status of Seabirds of the Southern California Bight. Part III of Investigator's Reports, Summary of Marine Mammal and Seabird Surveys of the Southern California Bight Area, 1975-1978. Final Report prepared by the University of California, Santa Cruz, for the Bureau of Land Management, Contract No. AA550-CT7-36. National Technical Information Service, Springfield, Virginia: NTIS # PB-81-248-197. 470 pp

British Antarctic Survey, BIOPEARL I expedition JR 144 on RRS James Clark Ross

Burns, Jennifer M., Daniel P. Costa. The movements of 35 seals were monitored via satellite, Antarctic, 2001 and 2002, GLOBEC

Caadas, A. and Hammond, P. 2006. Model-based abundance estimate of bottlenose dolphins off Southern Spain: implications for conservation and management. Journal of Cetacean Research and Management: 8(1):13-27

Caadas, A. and Hammond, P.S. In press. Abundance and habitat preferences of the short-beaked common dolphin (*Delphinus delphis*) in the South-western Mediterranean: implications for conservationEndangered Species Research:

Caadas, A., R. Sagarminaga, R. de Stephanis, E. Urquiola and P.S. Hammond. 2005. Habitat selection models as a conservation tool: proposal of marine protected areas for cetaceans in Southern SpainAquatic Conservation: Marine and Freshwater Ecosystems: 15:495-521

Canadian Geospatial Data Infrastructure. Biological Investigations of Marine Antarctic Systems and Stocks (BIOMASS) Data Set Marine Ornithology:

20: 51-59 Biomass Working Party on Bird Ecology. 1992. Recording distribution and abundance of seabirds at sea in the Southern Ocean: Methods used in the BIOMASS programme.BIOMASS Report Series: 10 BIOMASS. 1979. Antarctic krill biology. First Report of the Working Party on Krill Biology.BIOMASS Handbook: 16

Carbotte, S.M., R. Arko, D.N. Chayes, W. Haxby, K. Lehnert, S. OHara, W.B.F. Ryan, R.A. Weissel, T. Shipley, L. Gahagan, K. Johnson, T. Shank (2004), New Integrated Data Management System for Ridge2000 and MARGINS Research, Eos Trans. AGU, 85(51), 553, doi:10.1029/2004EO510002. Consulted on http://www.iobis.org on [date].

Carin J. Ashjian, Robert G. Campbell, Harold E. Welch, Mari Butler,Donna Van Keuren. (2003) Annual cycle in abundance, distribution, and size in relation to hydrography of important copepod species in the western Arctic Ocean (SHEBA). Deep Sea Research, 50: 1235-1261 <http://science.nature.nps.gov/nrdata

Carol Roden, NOAA Southeast Fisheries Science Center. 1994. Summer Eastern Gulf of Mexico Marine Mammal Survey; Oregon II Cruise 212 (94-04). :

Carol Roden, Perry Thompson, NOAA Southeast Fisheries Science Center. 1994. Cruise Results; Marine Mammal Survey and Spring Southeast Are Monitoring and Assessment Program (SEAMAP) Ichthyoplankton Survey; NOAA Ship Oregon II Cruise OT-94-01 (209). :

Carol Roden. 1999. Cruise Results; Summer Atlantic Ocean Marine Mammal Survey; NOAA Ship Oregon II Cruise OT 99-05 (236).

Carrie W. Hubard, Keith D. Mullin, NOAA Southeast Fisheries Science Center. 2000. Report of a Cetacean Survey of Oceanic Waters of the Northern Gulf of Mexico aboard NOAA Ship Gordon Gunter (Cruise 007). :

Carrie W. Hubard, Keith D. Mullin, NOAA Southeast Fisheries Science Center. 2001. Report of a Cetacean Survey of Oceanic Waters of the Northern Gulf of Mexico during NOAA Ship Gordon Gunter Cruise GU-01-02 (012). :

Carrie W. Hubard. Keith D. Mullin, NOAA Southeast Fisheries Science Center. 1999. Report of a Cetacean Survey of Oceanic Waters of the Northern Gulf of Mexico aboard NOAA Ship Oregon II (Cruise 234). :

Carter, I. C., Williams, J. M., Webb, A., & Tasker, M. L. . 1993. Seabird concentrations in the north Sea: an atlas of vulnerability to surface pollutants. Joint Nature Conservation Committee report, Peterborough

CEFAS. - UK. Macrobenthos from English waters between 2000-2002. http://www.vliz.be/vmdcdata/nsbp.

Cetacean and Turtle Assessment Program, University of Rhode Island. 1982. A Characterization of Marine Mammals and Turtles in the Mid- and North-Atlantic Areas of the U.S. Outer Continental Shelf, Final Report.

Cetacean and Turtle Assessment Program, University of Rhode Island. 1981. A Characterization of Marine Mammals and Turtles in the Mid- and North-Atlantic Areas of the U.S. Outer Continental Shelf, Annual Report for 1979.

Cetacean and Turtle Assessment Program, University of Rhode Island. 1982. A Characterization of Marine Mammals and Turtles in the Mid- and North-Atlantic Areas of the U.S. Outer Continental Shelf, Annual Report for 1980.

Cetacean and Turtle Assessment Program, University of Rhode Island. 1982. A Characterization of Marine Mammals and Turtles in the Mid- and North-Atlantic Areas of the U.S. Outer Continental Shelf, Final Report.

Chavan, VIshwas and C. T. Achuthankutty (editors), IndOBIS Catalogue of Life, Available at http://www.indobis.org/,

Christin Khan, Timothy V.N. Cole, Peter Duley, Allison Glass, Misty Niemeyer, and Cynthia Christman. 2008. North Atlantic Right Whale Sighting Survey (NARWSS) and Right Whale Sighting Advisory System (RWSAS): 2008 Results Summary. Northeast Fisheries Science Center Reference Document 09-05</a

Clark, D. and Branton, B., DFO Maritimes Research Vessel Trawl Surveys, OBIS Canada Digital Collections,2007, Bedford Institute of Oceanography, Dartmouth, Nova Scotia, Canada, OBIS Canada, 1, Digital

Continuous Plankton Recorder (CPR) data from the Sir Alister Hardy Foundation for Ocean Science (SAHFOS). Avaiable from http://iobis.org/

Cooper, K.M., Boyd, S.E. and Rees, H.L, Cross Sands broadscale survey 1998. EUROBIS, Centre for Environment, Fisheries and Aquaculture, Burnham laboratory, 05 Apr 2006, Essex, UK, Version: 2006-03-31, MS EXCEL, http://www.marbef.org/

Copson, G.R., Rounsevell, D.E. (1986) The abundance of royal penguins (*Eudyptes schlegeli* , Finsch) breeding at Macquarie Island. ANARE Research Notes 41. 11 pp

Coral Reef Ecosystem Division (CRED), NOAA Pacific Island Fisheries Science Center, 2008-05-08, CRED Rapid Ecological Assessment of Invertebrate in the Pacific Ocean, from 2002 to 2008

Coral Reef Ecosystem Division (CRED), NOAA Pacific Island Fisheries Science Center, 2008-05-08, CRED Rapid Ecological Assessment of Benthic Habitat Cover in the Pacific Ocean, from 2005 to 2009

Coral Reef Ecosystem Division (CRED), NOAA Pacific Island Fisheries Sciences Center, 2008-05-08, CRED REA Algal Quadrate Images in the Pacific Ocean, from 2002 to 2008

Coral Reef Ecosystem Division (CRED), Pacific Island Fisheries Sciences Center, National Marine Fisheries

Service, 2008-05-08, CRED Rapid Ecological Assessments of Fish Belt Transect Surveys and Fish Stationary Point Count Surveys in the Pacific Ocean, from 2000 to 2009
Coral Reef Ecosystem Division (CRED), Pacific Island Fisheries Sciences Center, NOAA National Marine Fisheries Service, 2008-05-08, CRED Rapid Ecological Assessments of Coral Population in the Pacific Ocean, from 2007 to 2009
Coral Reef Ecosystem Division (CRED), Pacific Island Fisheries Sciences Center, National Marine Fisheries Service, 2008-05-08, CRED Rapid Ecological Assessments of Coral Health and Disease in the Pacific Ocean, from 2005 to 2008
Coral Reef Ecosystem Division (CRED), Pacific Islands Fisheries Sciences Center, NOAA National Marine Fisheries Service, 2008-05-08, CRED Towed-Diver Fish Biomass Surveys in the Pacific Ocean, from 2000 to 2009
Countryside Council for Wales, Marine Nature Conservation Review (MNCR) and associated benthic marine data held and managed by CCW. EUROBIS, Countryside Council for Wales, 19 Dec 2005, Gwynedd, UK, Version: tst, DiGIR Provider, http://www.ccw.gov.uk
Cousseau, M.B. and Perrotta, R.G. 2004. Peces marinos de Argentina. Biologa, distribucin, pesca. 3ra Ed., Publicaciones Especiales INIDEP, Mar del Plata, 167 pp.
Coyne, M. S., and B. J. Godley. 2005. Satellite Tracking and Analysis Tool (STAT): an integrated system for archiving, analyzing and mapping animal tracking data. Marine Ecology Progress Series. Vol. 301:1-7.
D. Gillespie, P. Berggren, S. Brown, I. Kuklik, C. Lacey, T. Lewis, J. Matthews, R. Mclanaghan, A. Moscrop And N. Tregenza. 2005. Relative abundance of harbour porpoises (*Phocoena phocoena*)Journal of Cetacean Research and Managment: 7(1):51-57
Dahle S., R. Palerud, N. Anisimova, 1992: Benthic fauna around Franz Josef Land. Akvaplan-niva, Norway.
Dahle S., S. Cochrane, 1992: Northern Barents Sea 1992. Akvaplan-niva, Norway
Dale Rostron, Pembrokeshire Marine Species Atlas. EUROBIS, Countryside Council for Wales, 11 Aug 2004, Gwynedd, UK, Version: 1, DiGIR Provider, http://www.marbef.org/
Danis B, Jangoux M, 2008. Sea stars from the Marion Dufresne MD03 expedition in Kerguelen islands. Contribution to the SCAR-Marine Biodiversity Information Network.
Danis B, Wilmes J, Jangoux M, 2008. Sea stars from the ANDEEP 3 expedition. Contribution to the SCAR-Marine Biodiversity Information Network.
Danis B, Wilmes J, Jangoux M, 2008. Sea stars from the EPOS 3 expedition. Contribution to the SCAR-Marine Biodiversity Information Network.
Danis B, Wilmes J, Jangoux M, 2008. Sea stars from the Walter Herwig FFS expedition. Contribution to the SCAR-Marine Biodiversity Information Network.
Danis B, Wilmes J, Tablado A, 2008. Sea stars from the Museo Argentino de Ciencias Naturales (MACN) collection. Contribution to the SCAR-Marine Biodiversity Information Network.
Danovaro, R. (1997). Meiofauna and nematodes from the Atacama slope and trench. Polytechnic University of Marche; Faculty of Sciences; Department of Marine Sciences, Italy.
David Potter, National Marine Fisheries Service. 1995. CRUISE RESULTS; Cruise No. PE 95-02; Marine Mammal Survey.
David Potter, National Marine Fisheries Service. 1995. CRUISE RESULTS; Cruise No. PE 95-01; Marine Mammal Abundance Survey - Leg1.
David Potter, National Marine Fisheries Service. 1995. CRUISE RESULTS; R/V ABEL-J; Cruise No. AJ-95-01 (Part II); Summer Marine Mammal Survey.
David Potter, National Marine Fisheries Service. 2002. CRUISE REPORT; Cruise No. DE 02-06; Joint Deepwater Systematics and Marine Mammal Survey.
David W., Johnston, Jooke Robbins, Marie E. Chapla, David K. Mattila, and Kimberly R. Andrews. 2008. Diversity, habitat associations, and stock structure of odontocete cetaceans in the waters of American SamoaJournal of Cetacean Research and Management:
De Broyer C., Lowry J.K., Jazdzewski K. & Robert H., 2007. Catalogue of the Gammaridean and Corophiidean Amphipoda (Crustacea) of the Southern Ocean with distribution and ecological data. In: De Broyer, C. (ed.) Census of Antarctic Marine Life. Synopsis of the Amphipoda of the Southern Ocean. /Bulletin de lInstitut Royal des Sciences Naturelles de Belgique, Biologie / 77(Suppl.1, part 1): 1-325.
De Olivera Santos, M. C.; Siciliano, S.; Pacheco de Souza, S. and Altamayer Pizzorno, J. L. 2001. Occurrence of southern right whales (*Eubalaena australis*) along southeastern Brazil. J. Cetacean Res. Manage. (Special ISSUE) 2, 153-156.
Debra Palka, David Potter, National Marine Fisheries Service. 1991. CRUISE REPORT OF THE HARBOR PORPOISE SURVEY - 1991 AJ91-02.
Debra Palka, David Potter, National Marine Fisheries Service. 1992. CRUISE REPORT OF THE HARBOR PORPOISE SURVEY - 1992; AJ92-01. :
Deprez, T. (2000). NeMys, A Generic webbased Taxonomic Information System. http://intramar.ugent.be/nemys.
Dewarumez J.-M. & Desroy N. - 2000. Eastern Channel dataset. Station Marine de Wimereux, France.
Dewarumez, J.-M., Dauvin, J.-C. & Desroy, N - 2000. Macrobenthos from Copale - Authie. Station Biologie de Wimereux, France.
Dohl, T. P., K. S. Norris, R. C. Guess, J. D. Bryant and M. W. Honig. 1981. Cetacea of the Southern

California Bight. Part II of Investigator's Reports, Summary of Marine Mammal and Seabird Surveys of the Southern California Bight Area, 1975-1978. Final Report prepared by the University of California, Santa Cruz, for the Bureau of Land Management, Contract No. AA550-CT7-36. National Technical Information Service, Springfield, Virginia: NTIS # PB81248189. 414 pp

Done T (1994) Australian Institute of Marine Science - Great Barrier Reef nearshore coral diversity.

Dr. M. J. Caley (2009). Australian Institute of Marine Science CReefs: Ningaloo Reef Biodiversity Expedition. OBIS Australia

Dr. M. J. Caley (2009). Australian Institute of Marine Science CReefs: Lizard Island Biodiversity Expedition. OBIS Australia

Dr. M. J. Caley (2009). Australian Institute of Marine Science CReefs: Heron Island Biodiversity Expedition. OBIS Australia

Duggan S and McKinnon AD (2002). Australian Institute of Marine Science Zooplankton community composition of Darwin Harbour, Northern Territory.

EDER, E., LEWIS, M. (2005) Proximate composition and energy value of demersal and pelagic prey species from Southwest Atlantic. Marine Ecology Progress Series (MEPS) 291:43-52

Elena Arashkevich and Anna Pasternak. Arctic Ocean: Amundsen and Nansen basins ´┐¢ July-August 2001. Vertical and stratified zooplankton sampling onboard the Swedish icebreaker Oden, program FAMIZ

Emig C, Geographic distribution of Brachiopod species in the Antarctic - Subantarctic region, [year accessed]. available from the SCAR-MarBIN website.

English Nature, Marine Nature Conservation Review (MNCR) and associated benthic marine data held and managed by English Nature. EUROBIS, English Nature, 02 Jan 2006, Peterborough, UK, Version: 1, DiGIR Provider, http://www.marbef.org

Escribano, R., Hidalgo, P., Manrquez, K., 2005. Humboldt Current species.

Ezemvelo KwaZulu-Natal Wildlife - Benthic survey on Aliwal Shoal (May 2004 - May 2005)

Fabricius, K (1996). Australian Institute of Marine Science - Surveys of Octocoral communities, benthic cover and environmental factors on coral reefs of the Great Barrier Reef.

Fabricius, K (1999). Australian Institute of Marine Science - Surveys of Octocoral communities, benthic cover and environmental factors on coral reefs of Torres Strait.

Fabricius, K (1999). Australian Institute of Marine Science - Surveys of Octocoral communities, benthic cover and environmental factors on coral reefs of Hong Kong.

Fabricius, K (2002). Australian Institute of Marine Science - Surveys of Octocoral communities, benthic cover and environmental factors on coral reefs of Milne Bay, Papua New Guinea.

Fabricius, K (2005). Australian Institute of Marine Science - Surveys of Octocorals communities, benthic cover and environmental factors on coral reefs of Palau.

Fabricius, K (2007). Australian Institute of Marine Science - Surveys of Octocorals in the Rowley Shoals Marine Park and the Mermaid Reef National Marine Nature Reserve, Western Australia.

Feder HM, Jewett SC, Blanchard A. (2005) Southeastern Chukchi Sea (Alaska) epibenthos 1976. Polar Biology, 28: 402-421

Fernandez P., Anderson D.J., Sievert P.R., and Huyvaert K. 2001. Foraging destinations of three low-latitude albatross (Phoebastria) species. Journal of Zoology: 254: 391-404

Fevolden S (1979) Investigations on krill (Euphausiacea) sampled during the Norwegian Antarctic Research Expedition 1976-77 . Sarsia 64: 189-198

Fevolden, SE (1980) Krill off Bouvetya and in the southern Weddell Sea with a description of larval stages of Euphausia crystallorophias. Sarsia 65: 149-162

Fockedey N.; Beyst, B.; Cattrijsse, A.; Dewicke A.; Deneudt, K.; Mees J.; Vincx, M.(2004). Historical hyperbenthos data (1987-2001) from the North Sea and some adjacent areas. Collaboration between Ghent University (UGent), Biology Department, Marine Biology Section and Flanders Marine Institute (VLIZ).

Fortier, L. Darnis, G. (2006) Vertical distribution of arctic mesozooplankton in Franklin Bay, southern Beaufort Sea, during winter 2003-04

Fritsen, C. Bacteria and chlorophyll-a water column observations (surface to ~150m), April-August 2001, Continental Margin Western Antarctic Peninsula, GLOBEC

Froese, R. and D. Pauly. Editors. 200x. FishBase. World Wide Web electronic publication. www.fishbase.org,

Gambi, C. & Danovaro, R. (1992). Meiofauna of the Ligurian Sea. Polytechnic University of Marche; Faculty of Sciences, Department of Marine Sciences, Italy.

Gambi, C. & Danovaro, R. (1997). Meiofauna of the North Adriatic Sea. Polytechnic University of Marche, Faculty of Sciences, Department of Marine Sciences, Italy.

Garcâ¡a-Godos, I. 2006. A note on the occurrence of sperm whales (*Physeter macrocephallus*) off Peru, 1995-2002.Journal of Cetacean Research and Management (JCRM): 8(1):113-119

Garrison, V.H. 2010. Virgin Islands National Park Coral Transplant Study. U.S. Geological Survey Southeast Ecological Science Center, 600 Fourth Street South, St. Petersburg, Florida 33701.

Geernaert, T.G., Gilroy, H. L., Kaimmer, S. M., Williams, G. H., and Trumble, R. J. 2001. Feasibility study that investigates options for monitoring bycatch

of the short-tailed albatross in the Pacific halibut fishery off Alaska
Gerald Scott, Thomas Leming, Keith D. Mullin, Nelson May, NOAA Southeast Fisheries Science Center. 1992. Distribution and Abundance of Marine Mammals in the Northern-Central and Western Gulf of Mexico.: Alonzo. N. Hamilton, Jr., NOAA Southeast Fisheries Science Center. 1992. Cruise Results; SEAMAP Spring Ichthyoplankton Survey; NOAA Ship Oregon II Cruise 92-02 (199). :
Gheskiere, T. (2000). Nematode assemblages from a Belgian sandy beach. Ghent University, Department of Biology, Marine Biology Section (MARBIOL), Belgium.
Gheskiere, T. (2000). Nematodes from Italy and Poland. Ghent University, Department of Biology, Marine Biology Section (MARBIOL), Belgium.
Gilbert, J.R.,G.T. Waring, K.M.Wynne, and N. Guldager. 2005. Changes in abundance of harbor seals in Maine, 1981-2001Marine Mammal Science: 21(3):519-535 Dow, W. E. 2005. Digital Atlas of Seal Haul-out Sites in Maine: 1981-2001. Masters of Environmental Management Masters Project:
Girard, C., A. D. Tucker, and B. Calmettes. 2009. Post-nesting migrations of loggerhead sea turtles in the Gulf of Mexico: dispersal in highly dynamic conditions. Marine Biology. 156:1827-1839.
Goodall, R. N. P. and Macnie, S. V. 1998. Sightings of Pilot Whales off South America South of 30 S: A review of data to 1988. Rep. Int. Whal. Cmmn 48.
Goodall, R. N. P.; de Haro, J. C.; Fraga, F; Iiguez, M. A. and Norris, K. S.. 1997. Shighting and behaviour of Peal`s Dolphins, *Lagenorhynchus australis*, with notes on Dusky Dolphins, *L. obscurus*, off southenmost south America. REP. INT. WHAL. CONMMN 47, 757-775.
Goodall, R. N. P.; Galeazzi, A. R. and Lichter, A. A. 1988. Exploitation of small cetaceans off Argentina 1979-1986. Rep. Int. Whal. Commn 38. Cristín de Haro, J. and Iiguez, A. M. 1997. Rep. Int. Whal. Commn 47.
Goodall, R. N. P.; Galeazzi, A. R.; Leatherwood, S.; Miller, K. W.; Cameron, I. S.; Kastelein, R. K. and Sobral, A. P.. 1998. Studies of Commerson's Dolphins, *Cephalorhynchus commersonii*, off Tierra del Fuego, 1976-1984, with a Review of Information on the Species in the South Atlantic. Rep. Int. Whal. Commn. (special isue 9)
Goodall, R. N. P.; Norris, K. S.; Schevill, W. E.; Fraga, F.; Praderi, R.; Iiguez, M. A. and de Haro, C.. 1997. Review and update on the biology of Peale`s dolphin, *Lagenorhynchus australis*. REP. INT. WHAL. COMMN 47, 777-796.
Gradinger et al. (2005) Meiofauna in sea ice of the Beaufort Gyre 2002 and 2003. Polar Biology, 171-184
Green, G. A., J. J. Breuggeman, C. E. Bowlby, R. A. Grotefendt, M. L. Bonnell, and K. T. Balcomb, III. 1991. Cetacean Distribution and Abundance Off Oregon and Washington, 1989-1990. Final Report prepared by Ebasco Environmental, Bellevue, WA, and Ecological Consulting, Inc. , Portland, OR, for the Minerals Management Service, Pacific OCS Region. OCS Study MMS: 91-0093. 100 pp
Greig, A. B.; Secchi, E. R.; Zerbini, A. N.; and Dalla Rosa, L. 2001, Stranding events of southern right whale, *Eubalaena australis*, in southern Brazil. J. Cetacean Res. Manage. (Special ISSUE) 2, 157-179
Gross, A., Kiszka, J., Van Canneyt, O., Richard, P. & Ridoux, V.. Submitted. Habitat and resource partitioning among a community of tropical dolphins around Mayotte (Mozambique Channel): a case study on tropical dolphinsEstuarine, Coastal and Shelf Sciences: Kiszka, J., Ersts, P. & Ridoux, V.. In prep.. Distribution, encounter rates and group characteristics of cetaceans around the Mozambique Channel island of Mayotte.Marine Biology:
Hafsteinn G. Gudfinnson, Hgni Debes, Tone Falkenhaug, Eilif Gaard, stthor Gislason, Hildur Petursdottir, Thorsteinn Sigurdsson, and Hedinn Valdimarsson. 2008. Abundance and productivity of the pelagic ecosystem along a transect across the northern Mid- Atlantic Ridge in June 2003. ICES CM 2008/C:12
Haimovici, M.; üvila-da-Silva, A. O.; Rossi-Wongtschowski, C. L. D. B. 2004. Prospecºúo pesqueira de espcies demersais com espinhel-de-fundo na Zona Econmica Exclusiva da regiúo Sudeste-Sul do Brasil. Súo Paulo : Instituto Oceanogrífico da USP, 2004. (Srie Documentos Revizee: Score Sul). 112 p. ISBN 85-98729-01-9.
Hammond, P.S., Berggren, P., Benke, H., Borchers, D.L., Collet, A., Heide-Jâ©rgensen, M.P., Heimlich, S., Hiby, A.R., Leopold, M.F. & âÿien, N. 2002. Abundance of harbour porpoises and other cetaceans in the North Sea and adjacent waters. Journal of Applied Ecology. 39: 361-376
Harris R. & Widdicombe C. (2007). L4 Plankton Monitoring Programme. Plymouth Marine Laboratory (PML), United Kingdom.
Heip, C. (1986). Nematodes from the North Sea Benthos Survey. Netherlands Institute of Ecology, Centre for Estuarine and Marine Ecology (NIOO-CEME), The Netherlands.
Hellenic Centre For Marine Research, MedOBIS. EUROBIS, Hellenic Centre for Marine Research; Institute of Marine Biology and Genetics; Biodiversity and Ecosystem Management Department, 17 Dec 2004, Heraklion, Greece, Version: 1, MS Access, http://www.medobis.org/
Herman, R. (1984). Copepods from the Southern Bight of the North Sea. Ghent University, Department of Biology, Marine Biology Section (MARBIOL), Belgium.
Herrmann, M., J. Laudien, 2004: Soft bottom community structure and diversity in Arctic Kongsfjorden. Alfred-Wegener-Institute for Polar and Marine Research, Bremerhaven, Germany

Hill, P. Scott, Alan Jackson, and Tim Gerrodette. 1990. Report of a Marine Mammal Survey of the Eastern Tropical Pacific aboard the R/V David Starr Jordan, July 29-December 7, 1989NOAA-TM-NMFS-SWFC-142:

Hill, P. Scott, Alan Jackson, and Tim Gerrodette. 1990. Report of a Marine Mammal Survey of the Eastern Tropical Pacific aboard the R/V Mcarthur, July 29-December 7, 1989 NOAA-TM-NMFS-SWFC-143:

Hill, P. Scott, Alan Jackson, and Tim Gerrodette. 1991. Report of a Marine Mammal Survey of the Eastern Tropical Pacific aboard the R/V Mcarthur, July 28-December 6, 1990 NOAA-TM-NMFS-SWFSC-159:

Hill, P. Scott, and Jay Barlow. 1992. Report of a Marine Mammal Survey of the California Coast aboard the Research Vessel Mcarthur July 28-November 5, 1991 NOAA-TM-NMFS-SWFSC-169:

Hill, P. Scott, Randall C. Rasmussen, and Tim Gerrodette. 1991. Report of a Marine Mammal Survey of the Eastern Tropical Pacific aboard the R/V David Starr Jordan, July 28-December 6, 1990NOAA-TM-NMFS-SWFSC-158:

Hoggard, W., Rohers, C., Pickett, M., Blaylock, B., Roden, C., O'Sullivan, S, Garrison, L. 1995. Mid Atlantic Tursiops Surveys 1995. Southeast Fisheries Science Center:

Holt, Rennie S., and Alan R. Jackson. 1987. Report of a Marine Mammal Survey of the Eastern Tropical Pacific aboard the Research Vessel Mcarthur, July 29-December 6, 1986NOAA-TM-NMFS-SWFC-77:

Holt, Rennie S., and Alan R. Jackson. 1988. Report of a Marine Mammal Survey of the Eastern Tropical Pacific aboard the Research Vessel Mcarthur, July 30-December 10, 1987NOAA-TM-NMFS-SWFC-116:

Holt, Rennie S., and Stephaine N. Sexton. 1987. Report of a Marine Mammal Survey of the Eastern Tropical Pacific aboard the Research Vessel David Starr Jordan, July 29-Dec.5,1986NOAA-TM-NMFS-SWFC-76:

Holt, Rennie S., and Stephaine N. Sexton. 1988. Report of a Marine Mammal Survey of the Eastern Tropical Pacific aboard the R/V David Starr Jordan, August 8-December 10, 1987NOAA-TM-NMFS-SWFC-117:

Holt, Rennie S., and Stephaine N. Sexton. 1989. Report of a Marine Mammal Survey of the Eastern Tropical Pacific aboard the Research Vessel David Star Jordan July 28-Dec. 6 1988NOAA-TM-NMFS-SWFC-129:

Hopky, G.E. Lawrence, M.J. and Chiperzak, D.B.; Central and Arctic Region, Department of Fisheries and Oceans, Winnipeg, Manitoba R3T 2N6. (2004) NOGAP B2; Zooplankton Data from the Canadian Beaufort Sea Shelf, 1986. Canadian Data Report of Fisheries and Aquatic Sciences: 923

Hopky, G.E. Lawrence, M.J. and Chiperzak, D.B.; Central and Arctic Region, Department of Fisheries and Oceans, Winnipeg, Manitoba R3T 2N6. (1994) NOGAP B2; Zooplankton Data from the Canadian Beaufort Sea Shelf, 1984 and 1985. Canadian Data Report of Fisheries and Aquatic Sciences: 922.

Hopky, G.E. Lawrence, M.J. and Chiperzak, D.B.; Central and Arctic Region, Department of Fisheries and Oceans, Winnipeg, Manitoba R3T 2N6. (2004) NOGAP B2; Zooplankton Data from the Canadian Beaufort Sea Shelf, 1986. Canadian Data Report of Fisheries and Aquatic Sciences: 923

http://globec.whoi.edu/globec-dir/data-acknowledgement-policy.html</a

Hummel, H., W.C.H Sistermans & V. Escaravage, 2004: BIS dataset of the south-western part of Netherlands (1985-2004). Benthos information System, Monitor Taskforce - NIOO-CEME, Netherlands

Huotong, C. & Vincx, M. (1986). The meiobenthos of the Southern Bight of the North Sea. Ghent University, Department of Biology, Marine Biology Section (MARBIOL), Belgium.

Huys, R. & De Smet, G. (1993). Copepoda from the Dutch Continental Shelf, spring 1993. Ghent University, Department of Biology, Marine Biology Section (MARBIOL), Belgium.

Huys, R. (1984). Copepods from a sublittoral sandy station in the North Sea. Ghent University, Department of Biology, Marine Biology Section (MARBIOL), Belgium.

Hyrenbach, K.D., and Dotson, R.C. 2001. Post-breeding movements of a male Black-footed Albatross Phoebastria nigripesMarine Ornithology: 29:23-26

Hyrenbach, K.D., and Dotson, R.C. 2003. Assessing the susceptibility of female Black-footed Albatross (Phoebastria nigripes) to longline fisheries during their post-breeding dispersal: an integrated approachBiological Conservation: 112: 391-404

Ignacio Garcâ¡a-Godos. 2004. Killer whale (*orcinus orca*) occurrence off peru, 1995 – 2003. Latin American Journal of Aquatic Mammals (LAJAM): 3(2):177-180

INVEMAR. SIBM en lnea: Sistema de Informacin sobre Biodiversidad Marina. Santa Marta: Instituto de investigaciones Marinas y Costeras Jos Benito Vives de Andris,. http://www.invemar.org.co/siam/sibm/index.htm

Jaime Bolas-Jimnez, Dagmar Fertl, and Miguel Iguez. In press. A note on killer whale (*Orcinus orca*) occurrence in Venezuelan waters, 1982-2008.

Jensen, P. & Vincx, M. (1973). Nematode fauna from the bottom of the Southern North Sea. Ghent University, Department of Biology, Marine Biology Section (MARBIOL), Belgium.

Jian, L. (1992). Free-living nematodes in a brackish tidal flat of the Westerschelde. Ghent University, Department of Biology, Marine Biology Section (MARBIOL), Belgium.

John van den Hoff, Clive R. McMahon and Iain Field Tipping back the balance: recolonization of the Macquarie Island isthmus by king penguins

(Aptenodytes patagonicus) following extermination for human gain Antarctic Science , First View article doi:10.1017/S0954102009001898, Published Online by Cambridge University Press 10 Mar 2009
Johnston TH. (2000) Whale and Seal Observations 1929-31 and 1930-31 Pp 30-65. in Shaughnessy, P.D. (ed.) 2000 Antarctic seals, whales and dolphins of the early twentieth century: Marine mammals of the Australasian Antarctic Expedition 1911-14 (AAE) and the British, Australian and New Zealand Antarctic Research Expedition 1929-31 (BANZARE), ANARE Reports 142, 172pp.
K. Smedbol, J. Gale, L. Bajona, 2005, Canada Maritimes Regional Cetacean Sightings
Kappes, M.A., Shaffer, S.A., Tremblay, Y., Foley, D.G., Palacios, D.M., Robinson, P.W., Bograd, S.J., and Costa, D.P. 2009. Hawaiian albatrosses track interannual variability of marine habitats in the North Pacific. Progress in Oceanography. In press
KathrinKrabbe, FlorianLeese, ChristophMayer, RalphTollrian and ChristophHeld. Polar Biology Volume 33, Number 3 / March, 2010. p281-292.
Kedra M. (2006). Kongsfjorden monitoring data grid 2006. Polish Academy of Sciences, Institute of Oceanology (IOPAS), Poland.
Keith D. Mullin, Carol L. Roden, NOAA Southeast Fisheries Science Center. 1996. Report of a Cetacean Survey of Oceanic and Selected Continental Shelf Waters of the Northern Gulf of Mexico aboard NOAA Ship Oregon II (Cruise 220). :
Keith D. Mullin, Carol L. Roden, NOAA Southeast Fisheries Science Center. 1997. Report of a Cetacean Survey of Oceanic and Selected Continental Shelf Waters of the Northern Gulf of Mexico aboard NOAA Ship Oregon II (Cruise 225). :
Keith D. Mullin, Denice M. Drass, NOAA Southeast Fisheries Science Center. 2000. Report of a Cetacean Survey of Continental Shelf Waters of the Northern Gulf of Mexico aboard NOAA Ship OREGON II (Cruise 242). :
Keith D. Mullin, Robert Ford. 1992. OREGON II Cruise 92-01 (198).
Kendall, M., S. Widdicombe, 1999: Plymouth sound dataset. Soft sediment macrobenthos from the Plymouth Sound from 1995. Plymouth Marine Laboratory, UK
Koninklijk Nederlands Instituut voor Zeeonderzoek (Texel), National Institute for Coastal and Marine Management (Ministery of Transport and Public Works) - 2001. Macrobenthos in the Dutch Sector of the North Sea 1991-2001.
Kopczyska, E.E, Savoye, N., Dehairs, F., Cardinal, D. and Elskens, M. (2007) Spring phytoplankton assemblages in the Southern Ocean between Australia and Antarctica. Polar Biology 31: 77-88
Kotwicki, L. (1998). Meiobenthic data Manuela. Polish Academy of Sciences, Institute of Oceanology (IOPAS), Poland.
Koubbi P., Goffart A., Hecq J.H., Swadling K., Beans C., Wright S., 2007. Plankton and ichthyoplankton database of the Icota-Pelagant Programmes from Terre Adlie to the Mertz Glacier Tongue, East-Antarctica, in January 2004.
LAJAM 2008. A note on killer whale (*Orcinus orca*) occurrence in Venezuelan waters, 2001-2008. IWC SC60/SM8</a
Lambshead, J. (1978). Nematode data from the Firth of Clyde (Scotland). Natural History Museum (NHM), UK.
Lampadariou, N. (1992). Malia nematodes. Hellenic Centre for Marine Research (HCMR), Greece.
Lampadariou, N. (1992). Nematodes from Crete sandy beaches. Hellenic Centre for Marine Research (HCMR), Greece.
Lampadariou, N. (1993). Heraklion harbour meiobenthos. Hellenic Centre for Marine Research (HCMR), Greece.
Lampadariou, N. (1998). Aegean Sea bathyal nematodes. Hellenic Centre for Marine Research (HCMR), Greece.
Lanna, Campos, Bassoi. 2008. South American Antarctic MarineBiodiversity Literature.
Larsen, P.F. 2009. A Historical Record of Sponges, Bryozoa and Ascidians on the Coast of Maine:1843-1980. Bigelow Laboratory for Ocean Sciences Tech. Rept. 200905. West Boothbay Harbor, Maine.
Larsen, P.F., A.C. Johnson and A. Pugh. 2010. The Macrobenthos of Penobscot Bay, Maine. Bigelow Laboratory for Ocean Sciences.
Larsen, P.F., A.C. Johnson. L.F. Dogget and A. Pugh. 2009. Environmental Benchmark Studies in Casco Bay - Portland Harbor, Maine, April 1980. NOAA Technical Memorandum NMFS-F/NEC-19. 173 pp.
Lavrado, H.P. & Ignacio, B.L. (eds.) 2006. Biodiversidade bennica da costa central da Zona Econmica Exclusiva brasileira. Rio de Janeiro : Museu Nacional, 2006.(Srie Livros; 18) 389 p.
Leibniz-Institut fr Meereswissenschaften an der Universitt Kiel - Germany. Amrum Bank and inner German Bight Benthos. http://www.vliz.be/vmdcdata/nsbp.
Levin LA, Gage JD, Martin C, Lamont PA (2000) Macrobenthic community structure within and beneath the oxygen minimum zone, NW Arabian Sea. Deep-Sea Res II 47:189-226
Lewis, M.K. and D. Sameoto. 1987. The Vertical Distribution of Zooplankton and Ichthyoplabnkton in Davis Strait and Baffin Bay. August 1983. Can Data Report of Fish and Aquat Sci No 677;
Lindsay D. Antarctic Jellyfish, a literature-based compilation. Accessed online through the SCAR-MarBIN portal
Lrz and Held 2004 Molecular Phylogenetics and Evolution 31 (2004) 415
Luzuriaga M, D.Ortega, E.Elas 1998 - Aspectos bioecolgicos del ictioplancton en la estacin fija "La

Libertad" durante 1997. Acta oceanografica del Pacfico. INOCAR, Ecuador, 9(1), 145-151
Luzuriaga Ma. 1992.- Notas hidrolgicas de aguas superficiales del mar ecuatoriano seg║n indicadores biolgicos- foraminiferos planctnicos Acta Ocean. Pacifico INOCAR 7(1) Ecuador (Tesis Doctoral. Universidad de Guayaquil-Fac CCNN, 1980)
M. Cronin, C. Duck, O. O, Cadhla, R. Nairn, D. Strong and C. O'Keeffe. 2007. An assessment of population size and distribution of harbour seals in the Republic of Ireland during the moult season in August 2003Journal of Zoology: Vol. 273 pp. 131-139
M. Dahleim and J. Waite. . Abundance and Distribution of killer whales.
M. Dahlheim et al. 2000. Abundance and distribution of harbor porpoise (*Phocoena phocoena*) in Southeast Alaska and the offshore waters of Dixon Entrance to Prince William Sound, 1993. Marine Mammal Science: 16(1):28-45
M. Dalheim. Abundance and distribution of killer whales (*Orcinus orcs*) in Alaska, 1993.
M. Louella Dolar. 2006. Marine Mammals of the Marine Biodiversity Corridors in the Philippines: Verde Island Passage, Balabac Strait and Cagayan RidgeTechnical report submitted to Conservation International-Philippines:
M.E.Conkright, J.I. Antonov, O. Baranova, T.P. Boyer, H.E. Garcia, R. Gelfeld, D. Johnson, R.A. Locarnini, P.P., T.D. O'Brien, I. Smolyar, C. Stephens, 2002: World Pcean Database 2001, Voulme 1: Introduction. Ed: Sydney Levitus, NOAA Atlas NESDIS 42, U.S. Government Printing Office, Washinton, D.C., 167 pp.
M.N. De Boer. 2000b. Observations on occurence and distribution of cetaceans in the Southern Ocean Sanctuary and southern part of the Indian Ocean SanctuaryPaper SC/52/O9 presented to the IWC Scientific Committee, June 2000, in Adelaide, Australia: 10pp.
Macrozooplankton concentrations estimated from MOCNESS tow samples, Continental Margin Western Antarctic Peninsula, GLOBEC
Mahon,A.R., Arango,C.P. and Halanych,K.M. Mar. Biol. 155, 315-323 (2008)
Mangels, Karl F., and Tim Gerrodette. 1994. Report on Cetacean Sightings during a Marine Mammal Survey in the Eastern Tropical Pacific Ocean aboard the Noaa Ships Mcarthur and David Starr Jordan, July 28-November 6, 1993NOAA-TM-NMFS-SWFSC-211:
Mangels, Karl F., and Tim Gerrodette. 1994. Report on Cetacean Sightings during a Marine Mammal Survey in the Eastern Tropical Pacific Ocean aboard the Noaa Ships Mcarthur and David Starr Jordan, July 28-November 2, 1992NOAA-TM-NMFS-SWFSC-200:
Marchant M. 2006. Humboldt Current Planktic Foraminifera
Marine Conservation Society, Seasearch Marine Surveys. EUROBIS, Marine Conservation Society, 19 Jan 2005, Ross-on-Wye, UK, Version: 1, DiGIR Provided, http://www.marbef.org/
Marine Ecological Surveys Ltd. - UK. Macrobenthos from the eastern English Channel in 1999 and 2001. http://www.vliz.be/vmdcdata/nsbp.
Marine Invertebrate Diversity Initiative (MIDI) Database
Marine Resources Research Institute, South Carolina DNR
Mark C. Benfield and William M. Graham [date accessed]. In situ observations of S*tygiomedusa gigantea* in the Gulf of Mexico with a review of its global distribution and habitat.
Marks, K.W. 2007. ÔÇ£AGRRA Database, version (05/2007). Available online <http:www.agrra.org/Release_2007-05/
Martin, P [date accessed]. Southern Ocean oligochaete occurrence data - a literature-based compilation. accessed through the SCAR-MarBIN webportal.
Martnez Arbizu, P. & Veit-Khler, G. (2002). ANDEEP-1: Antarctic deep-sea meiofauna. Forschungsinstitut Senckenberg; Deutsches Zentrum fur Marine Biodiversittsforschung (DZMB), Germany.
Matschiner,M., Hanel,R. and Salzburger,W. Gene flow by larval dispersal in the Antarctic notothenioid fish Gobionotothen gibberifrons Mol. Ecol. 18 (12), 2574-2587 (2009)
Matthias Steffens. (2006) Distribution and structure of macrobenthic fauna in the eastern Laptev Sea in relation to environmental factors. Polar Biology, 29(10):837-848
Maughan, Ben. 2003. UK Royal Navy Cetacean Sightings
McEvoy, A. & Austen, M. (1996). Offshore nematodes from Rame (UK) and in microcosm experiment (exposure to metals). Plymouth Marine Laboratory (PML), UK.
McKinnon AD, Talbot S (1997) Australian Institute of Marine Science - Summer planktonic communities of North West Cape, Western Australia.
McKinnon, AD and Talbot, S (2000). Australian Institute of Marine Science Zooplankton community structure in Nearshore waters of the Great Barrier Reef.
McLellan, William. 2001. University of North Carolina at Wilmington Marine Mammal Sightings for Southeastern US
McLellan, William. 2002. University of North Carolina at Wilmington Marine Mammal Sightings for Southeastern US, 2002
Mecklenburg, C. W., T. A. Mecklenburg, B. A. Sheiko, and N. V. Chernova. 4 July 2006. Arctic marine fish museum specimens. Database submitted to ArcOD, Institute of Marine Science, University of Alaska Fairbanks by Point Stephens Research, P.O. Box 210307, Auke Bay, Alaska 99821 USA.

Michael Olesen (2010). Marine Benthic Fauna List, Island of L, Denmark. The Danish Biodiversity Information Facility, DanBIF. Copenhagen.

Mitchell, P.I., Newton, S.F., Ratcliffe, N. and Dunn, T.E., Seabird 2000. EUROBIS, Joint Nature Conservation Committee, 25 Jan 2006, Peterborough, UK, Version: 1, DiGIR Provider, http://www.marbef.org/

Mouritsen, H., Huyvaert, K.P., Frost, B.J., Anderson, D.J. 2003. Waved albatrosses can navigate with strong magnets attached to their head. Journal of Experimental Biology: 206 (22): 4155-4166

Mullin, K. D., Maze, S., Hubard, C. W.. 1998. Report of a Cetacean Survey of Continental Shelf Waters of the Northern Gulf of Mexico aboard NOAA Ship GORDON GUNTER (Cruise 001). :

Munda I. (1965). Benthic algal vegetation of Mjifjrdur. Scientific Research Centre of the Slovenian Academy of Sciences and Arts, Ljubliana, Slovenia.

Munda I. (1967). Characteristic features of the benthic algal vegetation along the Snaefellsnes peninsula. Scientific Research Centre of the Slovenian Academy of Sciences and Arts, Ljubljana, Slovenia.

Munda I. (1968). Survey of the benthic algal vegetation of the Berufjrdur, southeastern Iceland. Scientific Research Centre of the Slovenian Academy of Sciences and Arts, Ljubljana, Slovenia.

Munda I. (1969). Benthic algal vegetation of Borgafjrdur. Scientific Research Centre of the Slovenian Academy of Sciences and Arts, Ljubljana, Slovenia.

Munda I. (1969). Benthic marine algae in the Northern Adriatic Sea. Scientific Research Centre of the Slovenian Academy of Sciences and Arts, Ljubljana, Slovenia.

Munda, I. (1980). Macoralgae of the Tjornes Penisnula in the North of Iceland. Scientific Research Centre of the Slovenian Academy of Sciences and Arts, Ljubljana, Slovenia.

Myrna Lpez and William Bussing (1964): Museum of Zoology, Biology Department, University of Costa Rica

N.M. Voronina, Y.A. Rudyakov, B. Vilenkin, SOVIET ANTARCTIC EXPEDITIONS for Zooplankton. Contribution to the SCAR Marine Biodiversity Information Network (SCAR-MarBIN). Available online at http://www.scarmarbin.be

Nancy Jacobsen Stout, Linda Kuhnz, Lonny Lundsten, Kyra Schlining, Susan von Thun, (2002) Video Annotation and Reference System (VARS) database, Year 2000, Monterey Bay Aquarium Research Institute, Moss Landing, California USA, Database, www.mbari.org/vars

National Biodiversity Network Trust, Marine Turtles. EUROBIS, National Biodiversity Network Trust, 24 Jan 2006, Newark, UK, Version: 1 , DiGIR Provider, http://www.marbef.org/

Neilsen, Lavery & Lorz (2009) Synopsis of a new collection of sea spiders from the Ross Sea Antarctica , In press Polar Biology

Nichols,W.J., A. Resendiz, J.A.Seminoff, and B. Resendiz. 2000. Transpacific migration of a loggerhead turtle monitored by satellite telemetryBulletin of Marine Science: 67:937-47

NMFS-COPEPOD: the global plankton database. ONLINE. 2009. Available: http://www.st.nmfs.noaa.gov/plankton/index.html.

NOAA Northeast Fisheries Science Center. 2004. AERIAL SURVEY RESULTS; NOAA TWIN OTTER AIRCRAFT; Circle-Back Abundance Survey

NOAA Northeast Fisheries Science Center. 2004. Cruise Results; R/V ENDEAVOR; Cruise No. EN 04-395/396; Mid-Atlantic Marine Mammal Shipboard Abundance Survey

NOAA Southeast Fisheries Science Center. 1995. Oregon II Cruise 215 (95-01).

NOAAs National Marine Fisheries Service (NMFS) Northeast Fisheries Science Center (2005). Northeast Fisheries Science Center Bottom Trawl Survey Data. NOAAs National Marine Fisheries Service (NMFS) Northeast Fisheries Science Center. Woods Hole, Massachusetts, United States of America.

Norman B & Holmberg J (2008) ECOCEAN Whale Shark Photo-identification Library. Consulted on http://www.iobis.org on January 6, 2009. www.whaleshark.org

NPPSD. 2005. North Pacific Pelagic Seabird Database, Short-tailed Albatross. USGS Alaska Science Center & U.S. Fish and Wildlife Service, Anchorage, Alaska, USA: Version 2005.06.07

Olivier, F., Lee, A.V., Woehler, E.J. (2004) Distribution and abundance of snow petrels *Pagodroma nivea* in the Windmill Islands, East Antarctica. Polar Biology 27. 257-265 See Metadata record for details http://data.aad.gov.au/aadc/metadata/metadata_redirect.cfm?md=AMD/AU/SNPE84-03

Oppel, S., A. N. Powell, and D. L. Dickson. 2008. Timing and distance of King Eider migration and winter movements. Condor. 110: 296-305</a

Orfanidis S.; Tsiaga E. (2004). National monitoring of macrobenthos in the Kavala Gulf. National Agricultural Research Foundation; Fisheries Research Institute (FRI), Greece.

Orlando-Bonaca M. (2006). Rocky shore algal data from North Adriatic Sea (Piran) in 2006. Marine Biology Station Piran (MBSS), Slovenia.

Ostler, R., Marine Nature Conservation Review (MNCR) and associated benthic marine data held and managed by JNCC. EUROBIS, Joint Nature Conservation Committee, Centre for Ecology nd hydrology, 22 Nov 2005, Aberdeenshire, UK, Version: 1, DiGIR Provider, http://www.marbef.org

Oug, E. & B. Rygg (2000). Macrobenthos data from the Norwegian Skagerrak coast. Norwegian Institute for

Water Research (NIVA) and Norwegian Pollution Control Authority.
P. Jayasankar, Anoop A. Krishnan, M. Rajagopalan And P.K. Krishnakumar. 2007. A note on observations on cetaceans in the western Indian sector of the Southern Ocean (20-56 S and 45-57 30'E), January to March 2004. Journal of Cetacean Research and Management: 9(3):263-267
P. Lambardi, J.R.E. Lutjeharms, R. Mencacci, C.G. Hays, P. Luschi, . 2008. Influence of ocean currents on long-distance movement of leatherback sea turtles in the Southwest Indian OceanMar. Ecol. Prog. Ser.: 353: 289-301
P. Luschi, A. Sale, R. Mencacci, G.R. Hughes, J.R.E. Lutjeharms, F. Papi. 2003. Current transport of leatherback sea turtles (*Dermochelys coriacea*) in the oceanProc. R. Soc. Lond. B: 270 suppl. 2: 129-132
P. Luschi, J.R.E. Lutjeharms, P. Lambardi, R. Mencacci, G.R. Hughes, C.G. Hays. 2006. A review of migratory behaviour of sea turtles off southeastern AfricaS. Afr. J. Science: 102: 51-58
P.C. Pandey, N. Khare, and M. Sudhaker. 2006. Oceanographic research: Indian efforts and preliminary results from the Southern OceanCurrent Science: 90(7): 978-84.
Parr, J., Marine Life Information Network (MarLIN) marine survey data (Professional). Marlin, Collated Marine Life Survey Datasets, Marine Biological Association of the UK, 03 Dec 2005, Plymouth, UK, Version: 1.0, DiGIR Provider, http://www.marbef.org/
Parr, J., Marine Life Survey Data (collected by volunteers) collated by MarLIN. MarLIN, collated Marine Life Survey Datasets, Marine Biological Association of the UK, 04 Dec 2005, Plymouth, UK, Version: 1, DiGIR Provider, http://www.marbef.org/
Petryashov V. Geographic distribution of Lophogastrida species in the Antarctic and sub-Antarctic regions.
Petryashov. V.V. 2007. Biogeographical division of Antarctic and Subantarctic by Mysid (Crustacea: Mysidacea) fauna. Russian Journal of Marine Biology 33(1): 1-16
Phillips, L. M., A. N. Powell, and E. A. Rexstad. 2006. Large-scale movements and habitat characteristics of King Eiders throughout the nonbreeding period. Condor. 108: 887-900</a
Picton, B.E., C.S. Emblow, C.C. Morrow, E.M. Sides, P. Tierney, D. McGrath, G. McGeough, M. McCrea,P. Dinneen, J. Falvey, S. Dempsey, J. Dowse, and M. J. Costello, 1999: Marine sites, habitats and species data collected during the BioMar survey of Ireland. Environmental Sciences Unit, Trinity College, Dublin, Ireland
Pinchuk A Arctic Ocean Chuckchi Sea August 1953-1954 Zooplankton vertical stratified collections on board of the Russian R/V Lomonosov, program ANII A-65
Pohle, G., L. Van Guelpen, A. Martin, D. Welshman, and A. McGuire. 2004. Bay of Fundy Species
Polovina, J, G. H. Balazs, E. A. Howell, D. M. Parker, M. P. Seki and P. H. Dutton. 2004. Forage and migration habitat of loggerhead (*Caretta caretta*) and olive ridley (*Lepidochelys olivacea*) sea turtles in theFisheries Oceanography: 13:1, pp.36-51
Post, A. 1987. Pelagic transects of FRVs "Walther Herwig" and "Anton Dohrn" in the Atlantic Ocean 1966 to 1986. Mitt. Inst. f. Seefischerei d. BfaFi Hamburg, 42: 1-68.
Prez, D. and Guzmín, J. R.. 2006. Patrones de uso de la Ballena Franca Austral (*Eubalaena australis*) en la Baha Nueva (Puerto Madryn). Fundacin Ecocentro, Julio Verne 3784, (9120) Puerto Madryn, Chubut, Argentina. www.ecocentro.org.ar
Programa de Observadores a Bordo (POBCh) de la Secretaria de Pesca de la Provincia del Chubut, Argentina. Observer On board Program - Fisheries Secretariat of the Province of Chubut-Argentina (OOBPPCh).
R.R. Hopcroft, C. Clarke, R.J. Nelson, K.A. Raskoff. Zooplankton Communities of the Arctic´┐¢s Canada Basin: the contribution by smaller taxa. Polar Biology, 28: 198-206 <http://science.nature.nps.gov/nrdata
Raes, M. (2004). Nematoda from Kenya and Zanzibar. Ghent University, Department of Biology, Marine Biology Section (MARBIOL), Belgium.
Ramirez-Llodra, E., Blanco, 2005. ChEssBase: an online information system on biodiversity and biogeography of deep-sea fauna from chemosynthetic ecosystems. Version 2. World Wide Web electronic publications, http://www.noc.soton.ac.uk/chess/database/db_home.php
Read, A.J. & A.J. Westgate. 1997. Monitoring the movements of harbour porpoises (Phocoena phocoena) with satellite telemetryMarine Biology: 130: 315-322
Rees, H.L. et al., A comparison of benthic biodiversity in the North Sea, English Channel and Celtic Seas. EUROBIS, Centre for Environment, Fisheries and Aquaculture Science; Burnham Laboratory, 12 Apr 2005, Essex, UK, Version: 1, MS Excel, http://www.marbef.org/
Reid, J.B., Evans, P.G.H., & Northridge, S.P.. 2003. Atlas of Cetacean distribution in north-west European waters. Joint Nature Conservation Committee report, Peterborough,
Resendiz, A., B. Resendiz, W.J. Nichols, J.A. Seminoff, and N. Kamezaki. 1998. First confirmed east-west trans-Pacific movement of a loggerhead sea turtle, *Caretta caretta*, released in Baja California, MexicoPacific Science: 52(2): 151-153
REVIZEE South Score / Benthos - Amaral, A.C.Z. & Rossi-Wongtschowski, C.L.D.B. (eds.) 2004. Biodiversidade bentnica da regiúo sudeste-sul do Brasil, plataforma externa e talude superior. Súo

Paulo : Instituto Oceanogrífico da USP, 2004 (Srie Documentos Revizee - Score Sul). 216 p. ISBN 85-98729-08-6.
REVIZEE South Score / Pelagic and Demersal Fish Database Figueiredo, J. L.; Santos A. P.; Yamaguti, N.; Bernardes, R. A., Rossi-Wongtschowski, C. L. B. 2002. Peixes da Zona Econmica Exclusiva da regiúo Sudeste-Sul do Brasil. Súo Paulo : Editora da Universidade de Súo Paulo: Imprensa Oficial do Estado, 2002. 244 p.
Rigby,P.R., B.Konar, T.Kato, K.Iken, H.Chenelot and Y.Shirayama (2005). NaGISA OBIS Dataset ver.1
Rintoul, C, B. Schlagenhauf-Langabeer, K.D. Hyrenbach, K.H. Morgan, and W.J. Sydeman. 2006. Atlas of California Current Marine Birds and Mammals: Version 1. Unpublished report, PRBO Conservation Science, Petaluma, California
Robinson, P.W., Tremblay, Y., Crocker, D.E., Kappes, M.A., Kuhn, C.E., Shaffer, S.A., Simmons, S.E., Costa, D.P. 2007. A comparison of indirect measures of feeding behaviour based on ARGOS tracking data. Deep Sea Research II. 54: 356óÇô368
Roderick Hobbs and Janice Waite.. In press. Harbor Porpoise Abundance 1997-1999. Alaska Fisheries Science Center-National Marine Mammal Laboratory
Rogers A, Hall-Spencer J; Arnar Steingrmsson S; Wisshak M, Freiwald A, 2005. Cold-water corals: Version 2.0. UNEP World Conservation Monitoring Centre (UNEP-WCMC).
Rose, A. (2004). Major meiofauna taxa and Harpacticoida species from Hooksiel. Forschungsinstitut Senckenberg; Deutsches Zentrum fur Marine Biodiversittsforschung (DZMB), Germany.
Rosier, G., Posidonia Oceanica Survey 2005. EUROBIS, KennaEcodiving, 14 Jun 2006, Girona, Spain, Version: 1, MS Excel, http://www.marbef.org/
Rowntree, V. J.; Payne, R. S. and Schell, D. M. 2001. Changing patterns of habitat use by southern right whales (*Eubalaena australis*) on their nursery ground at Pennsula Valds, Argentina, and in their long-range movements. J. Cetacean Res. Manage. (Special ISSUE) 2, 133-143.
Ruiz GM, Fofonoff PW, Steves B, Huber T, Larson K, McCann L, Hitchcock NG, Hines AH, & Carlton JT. 2005. North American Sessile Marine Invertebrate Survey. http://invasions.si.edu/nemesis/
Rumohr, H. Holsatia-expedition 1887 - animals collected with a dredge during the expedition. Christian-Albrechts-University Kiel; Leibniz Institute of Marine Sciences; Marine Ecology Division; Benthos Ecology section, 11 Jun 2006, Kiel, Germany, http://www.marbef.org/
Rumohr, H., 1995: Kiel Bay intercalibration data set. Leibniz Institute of Marine Sciences, Marine Ecology Division, Germany
S. Oppel and A. N. Powell. 2009. Satellite telemetry of King Eiders in the western Arctic. online dataset</a
Sala, A.; Azzali, M.; Russo, A, 2000. Krill of the Ross Sea: distribution, abundance and demography of *Euphausia superba* and *Euphausia crystallorophias* during the Italian Antarctic Expedition (January-February 2000) Scientia Marina 66: 123-133
Salvatore Stampanato, Etude taxonomique et zoogographique des astrides des rgions antarctiques et subantarctiques, Brussels
Sameoto, D.D. 1984. Vertical Distribution of Zooplankton Biomass and species in Northeastern Baffin Bay Related to Temperature and Salinity. Polar Biol 2:213-224;
Sameoto, D.D. 1987. Vertical Distribution and Ecological Significance of Chaetognaths in the Arctic Environment of Bafin Bay. Polar Biol 7:317-328
Schller, M. 2010. SO-Polylist - a summary of Southern Ocean polychaete records.
Schnack, Klaus. (1998) Macrofaunal community patterns at the continental margin off East Greenland. Berichte zur Polarforschung: 294 <http://science.nature.nps.gov/nrdata
Schratzberger, M. (1996). Effects of physical disturbance on nematode communities in sand and mud. Centre for Environment, Fisheries and Aquaculture Science (CEFAS), UK.
Schratzberger, M. (1997). Effects of various types of disturbances on nematode communities. Centre for Environment, Fisheries and Aquaculture Science (CEFAS), UK.
Schratzberger, M. (1998). Effects of simulated deposition of dredged material on the structure of nematode assemblages: the role of burial. Centre for Environment, Fisheries and Aquaculture Science (CEFAS), UK.
Schratzberger, M. (1998). Structure of sublittoral meiofauna assemblages around the UK coast. Centre for Environment, Fisheries and Aquaculture Science (CEFAS), UK.
Schratzberger, M. (1999). Effects of simulated deposition of dredged material on the structure of nematode assemblages: the role of contamination. Centre for Environment, Fisheries and Aquaculture Science (CEFAS), UK.
Schratzberger, M. (1999). Structure of sublittoral nematode assemblages at four offshore stations around the UK. Centre for Environment, Fisheries and Aquaculture Science (CEFAS), UK.
Schratzberger, M. (2000). Impacts of chronic trawling disturbance on nematode communities. Centre for Environment, Fisheries and Aquaculture Science (CEFAS), UK.
Schratzberger, M. (2000). Impacts of experimental trawling disturbance on nematode communities. Centre for Environment, Fisheries and Aquaculture Science (CEFAS), UK.
Schratzberger, M. (2000). Structure of nematode communities in the south western North Sea. Centre for Environment, Fisheries and Aquaculture Science (CEFAS), UK.

Schratzberger, M. (2001). Effects of paint-derived tributyltin (TBT) on the structure of estuarine nematode assemblages in experimental microcosms. Centre for Environment, Fisheries and Aquaculture Science (CEFAS), UK.

Sea Mammal Research Unit. . Southern Elephant Seal pup telemetry from Macquarie Island, 1995-1996.

Sea Mammal Research Unit. Grey Seal Tracking in the North Sea, 1991-1993

Sexton, Stephanie N., Rennie S. Holt, and Alan R. Jackson. 1989. Report of a Marine Mammal Survey of the Eastern Tropical Pacific aboard the Research Vessel Mcarthur July 28-December 6, 1988NOAA-TM-NMFS-SWFC-128:

Shaffer, S.A., Tremblay, Y., Awkerman, J.A., Henry, W.R., Teo, S.L.H., Anderson, D.J., Croll, D.A., Block, B.A., and Costa, D.P. 2005. Comparison of light- and SST-based geolocation with satellite telemetry in free-ranging albatrosses. Marine Biology. 147: 833-843

Sharma, J. Vincx, M. (1980). A study of the nematode fauna of three estuaries in the Netherlands. Ghent University, Department of Biology, Marine Biology Section (MARBIOL), Belgium.

Sicinski J. 2008. Admiralty Bay Benthos Diversity Data Base (ABBED). Polychaeta. (http://www.scarmarbin.be)

Sicinski J., Bamber R. 2008. Admiralty Bay Benthos Diversity Data Base (ABBED). Pycnogonida. (http://www.scarmarbin.be)

Sicinski J., Blazewicz-Paszkowycz M. 2008. Admiralty Bay Benthos Diversity Data Base (ABBED). Cumacea. (http://www.scarmarbin.be)

Sicinski J., Blazewicz-Paszkowycz M. 2008. Admiralty Bay Benthos Diversity Data Base (ABBED). Tanaidacea. (http://www.scarmarbin.be)

Siegel, Robert E. 2010. SMCC Gulf of Maine Invertebrate Data. Southern Maine Community College, 2 Fort Road, South Portland, Maine 04106-1698, U.S.A. Retrieve from http://obisusa.nbii.gov.

Siegel, V. Krill occurence data from GAMLR (German Antarctic Marine Living Resources) Expedition

Silva-Hernandez, M.G.; Bolaâos-Jimenez, J.; Herrera-Trujillo, O. and Ferreira, C.. 2007. Abundancia y distribuciân de los cetâíceos presentes en la costa del estado Aragua. Thesis: 100

Skov,H, T. Gunnlaugsson, W.P. Budgell, J. Horne, L. Nttestad, E. Olsen, H. Siland, G. Vkingsson and G. Waring (2008) Small-scale spatial variability of sperm and sei whales in relation to oceanographic and topographic features along the Mid-Atlantic Ridge. Deep-sea Research II. 55: 254-268.

Smirnov I, Vasiljeva A, Konina T. Collections data on ecology of bottom animal of the Southern ocean

Smith-Vaniz, W.F., H.L. Jelks, and L.A. Rocha. 2010. USGS 2001 Buck Island National Monument Cryptic Fish Survey. U.S. Geological Survey, 7920 NW 71st Street, Gainesville, Florida 32653. Retrieve from http://obisusa.nbii.gov.

So. Calif Bight/MMS Surveys, Minerals Management Service (MMS), Pacific OCS Region

Soetaert, K. (1985). Length and width measurements of nematodes in the Ligurian Sea. Netherlands Institute of Ecology, Centre for Estuarine and Marine Ecology (NIOO-CEME), the Netherlands.

Soetaert, K. (1992). Length and width measurements of nematodes in the Indian Ocean. Netherlands Institute of Ecology, Centre for Estuarine and Marine Ecology (NIOO-CEME), the Netherlands.

Soetaert, K. (1993). Deep-sea meiobenthos. Netherlands Institute of Ecology, Centre for Estuarine and Marine Ecology (NIOO-CEME), the Netherlands.

Soetaert, K. (1993). Size of Atlantic nematodes. Netherlands Institute of Ecology; Centre for Estuarine and Marine Ecology (NIOO-CEME), the Netherlands.

Somerfield, P.J (1997). Nematodes from Kongsfjord, Svalbard. Plymouth Marine Laboratory (PML), UK.

Somerfield, P.J. & Austen, M. (1993). Meiofauna from Lynher estuary in microcosms with contaminated sediment from the Fal estuary. Plymouth Marine Laboratory (PML), UK.

Somerfield, P.J. (1981). Meiofauna from the Firth of Clyde (Scotland). Plymouth Marine Laboratory (PML), UK.

Somerfield, P.J. (1991). Liverpool Bay Nematoda and Copepoda (UK). Plymouth Marine Laboratory (PML), UK.

Somerfield, P.J. (1992). Nematoda and Copepoda from the Fal estuary (UK). Plymouth Marine Laboratory (PML), UK.

Somerfield, P.J. (1994). Nematodes of the Plymouth Sound. Plymouth Marine Laboratory (PML), UK.

Sousa-Pinto I. & Arajo, R. (2003). Intertidal rocky shore assemblages in Portugal. Centro Interdisciplinar de Investigaao Marinha e Ambiental (CIMAR), Portugal.

Sousa-Pinto I. & Arajo, R. (2003). Macroalgal communities of intertidal rock pools in Portugal. Centro Interdisciplinar de Investigaao Marinha e Ambiental (CIMAR), Portugal.

Southeast Fisheries Science Center, National Oceanic and Atmospheric Administration (year). NOAA Southeast Fishery Science Center (SEFSC) Commercial Pelagic Observer Program (POP) Data. Consulted on http://www.iobis.org on [date].

Southeast Fisheries Science Center, National Oceanic and Atmospheric Administration (year). NOAA Southeast Fishery Science Center (SEFSC) Fisheries Log Book System (FLS) Commercial Pelagic Logbook Data. Consulted on http://www.iobis.org on [date].

Southeast Fisheries Science Center. 1992. Marine Mammal Research Program. 1992. Southeast Cetacean Aerial Survey; January-March 1992. :

Southern Ocean Cumacea records by U. Muellenhardt-Siegel

Steinberg, D.K. and L.P. Madin (2003) Zooplankton Census. Bermuda Atlantic Time-series Study
Steiner, L., Silva, M.A., Zereba, J. & Leal, M.J. . 2007. Bryde's whales, *Balaenoptera edeni*, observed in the Azores: a new species record for the region. JMBA2 Biodiversity Records: 5728
Steyaert, M. (1994). Meiobenthos at the stations 115, 702, 790 on the Belgian Continental Shelf. Ghent University, Department of Biology, Marine Biology Section (MARBIOL), Belgium
Steyaert, M. (1996). Spatial heterogeneity of nematodes on an intertidal flat in the Westerschelde Estuary. Ghent University, Department of Biology, Marine Biology Section (MARBIOL), Belgium.
Steyaert, M. (1997). Tidal migration of nematodes on an estuarine tidal flat. Ghent University, Department of Biology, Marine Biology Section (MARBIOL), Belgium.
Steyaert, M. (1999). Meiobenthos at station 115bis - bentho-pelagic coupling. Ghent University, Department of Biology, Marine Biology Section (MARBIOL), Belgium.
Stocks, K. 2003. SeamountsOnline: an online information system for seamount biology. Version 3.1. seamounts.sdsc.edu
Stone, C. J., Webb, A., Barton, C., Ratcliffe, N., Reed, T. C., Tasker, M. L., Camphuysen, C. J., & Pienkowski, M. W. 1995. An atlas of seabird distribution in north-west European waters. Joint Nature Conservation Committee report, Peterborough,
Stone, C. J., Webb, A., Barton, C., Ratcliffe, N., Reed, T. C., Tasker, M. L., Camphuysen, C. J., & Pienkowski, M. W. . 1995 . An atlas of seabird distribution in north-west European waters. Joint Nature Conservation Committee report, Peterborough,
Sumner, F. B., R. C. Osborn, L. J. Cole, and B. M. Davis. A biological survey of the waters of Woods Hole and vicinity. Bulletin of the U.S. Bureau of Fisheries. 1911. 31: 1-860
Sweatman, H (1992) Australian Institute of Marine Science Long-term monitoring Program: Visual Census Fish Data (Great Barrier Reef).
Sweatman, H (2004) Australian Institute of Marine Science Long-term monitoring Program: Nearshore corals of the Great Barrier Reef.
The Norwegian Oil Industry Association, 2000: Offshore reference stations, Finnmark. The Norwegian Oil Industry Association (OLF), Akvaplan-niva and Det Norske Veritas, Norway
The Norwegian Oil Industry Association, 2001: Offshore reference stations, North/Norwegian sea. The Norwegian Oil Industry Association (OLF), Akvaplan-niva and Det Norske Veritas, Norway
The Norwegian Oil Industry Association, 2002: Offshore reference stations, Norwegian/Barents Sea. The Norwegian Oil Industry Association (OLF), Akvaplan-niva and Det Norske Veritas, Norway
Thornhill,D.J., Mahon,A.R., Norenburg,J.L. and Halanych,K.M. Molecular Ecology (2008) 17, 5104-5218
Tidal Creek Database, NOAA Oceans and Human Health Initiative, NOAA Hollings Marine Laboratory
Timothy V.N. Cole, Patricia Gerrior, and Richard L. Merrick. 2007. Methodologies and Preliminary Results of the NOAA National Marine Fisheries Service Aerial Survey Program for Right Whales (*Eubalaena glacialis*) in the Northeast U.S., 1998-2006. Northeast Fisheries Science Center Reference Document 07-02
Tom Fenchel, Bland J. Finlay (2004). Bay species list, Zealand, Denmark. The Danish Biodiversity Information Facility, DanBIF. Copenhagen.
Torben Wollf (2009). Galathea II, Danish Deep Sea Expedition 1950-52. Galathea Report. The Danish Biodiversity Information Facility, DanBIF. Copenhagen. Consulted on http://www.iobis.org on [date].
Tremblay, J. M. and Branton, B., DFO Maritimes Research Vessel Trawl Surveys Invertebrates, OBIS Canada Digital Collections,2007, Bedford Institute of Oceanography, Dartmouth, Nova Scotia, Canada, OBIS Canada, 1, Digital
Trkay, M., Senckenbergisches Sammlungsverwaltungssystem, SeSam. Senckenbergische Naturforschende Gesellschaft , 27 Oct 2006, Frankfurt, Germany, Version: 1.20, DiGIR Provider, http://sesam.senckenberg.de/
Tucker, A. D. 2009. Eight nests recorded for a loggerhead turtle within one season. Marine Turtle Newsletter. 124:16-17
Tutasi, P. 2005 Copepods Equatorial Eastern Pacific
United Kingdom Natural History Museum. Stranded Whale Recording Scheme, UK & Eire 1970-1979.
Urban-Malinga, B. (2001). Meiofauna from Kongsfjorden (Spitbergen, Arctic). Polish Academy of Sciences, Institute of Oceanology (IOPAS), Poland.
Urban-Malinga, B. (2004). Meiofauna of the Southern Baltic. Polish Academy of Sciences, Institute of Oceanology (IOPAS), Poland.
Vaitkus G. 2001. Spatial dynamics of regional wintering populations of seabirds in the gradient of wintering climatic conditions. Acta Zoologica Lituanica: 11 (3): 273-279
Van Gaever, S. (2000). Meiobenthos of the Darwin mounds (North-East Atlantic). Ghent University, Department of Biology, Marine Biology Section (MARBIOL), Belgium.
Vanaverbeke, J. (1993). Meiobenthos and nematodes from the continental shelf of the Laptev Sea. Ghent University, Department of Biology, Marine Biology Section (MARBIOL), Belgium.
Vanaverbeke, J. (1993). Meiofauna from the Goban Spur (OMEX) - 1993. Ghent University, Department of Biology, Marine Biology Section (MARBIOL), Belgium.

Vanaverbeke, J. (1994). Nematodes from the Goban Spur (OMEX) - 1994. Ghent University, Department of Biology, Marine Biology Section (MARBIOL), Belgium.
Vanaverbeke, J. (1998). Length, width and biomass measurements of nematodes from sandbanks on the Belgian Continental Shelf. Ghent University, Department of Biology, Marine Biology Section (MARBIOL), Belgium.
Vanaverbeke, J. (1999). Nematodes from station 330: structural and functional biodiversity on the Belgian Continental Shelf. Ghent University, Department of Biology, Marine Biology Section (MARBIOL), Belgium.
Vanaverbeke, J. et al., Meiobenthos of subtidal sandbanks on the Belgian Continental Shelf. Manuela Database, Universiteit Gent; Faculteit Wetenschappen; Vakgroep Biologie; Afdeling Mariene Biologie, 22 Mar 2005, Ghent, Belgium, Version: 1, MS Excel, http://www.marbef.org/
Vanaverbeke, J., Deprez, T. & Vincx, M. (2006). Meiobenthos of subtidal sandbanks on the Belgian Continental Shelf. Ghent University, Department of Biology, Marine Biology Section (MARBIOL), Belgium.
Vandenberghe, R. & Coomans, A. (1985). Study of the meiobenthos from a dumping site in the Southern Bight of the North Sea. Ghent University, Department of Biology, Marine Biology Section (MARBIOL), Belgium.
Vanhove, S. (1989). Nematodes from the Weddell Sea. Ghent University, Department of Biology, Marine Biology Section (MARBIOL), Belgium.
Vanhove, S. (2002). Nematodes from the South Sandwich Trench. Ghent University, Department of Biology, Marine Biology Section (MARBIOL), Belgium.
Vanreusel, A. (1985). Free-living nematodes of the Voordelta. Ghent University, Department of Biology, Marine Biology Section (MARBIOL), Belgium.
Vanreusel, A. (1993). Nematodes at two abyssal sites in the Northeast Atlantic. Ghent University, Department of Biology, Marine Biology Section (MARBIOL), Belgium.
Vanreusel, A. (1994). Nematodes of the central Arctic Ocean. Ghent University, Department of Biology, Marine Biology Section (MARBIOL), Belgium.
Veit-Khler, G. & Laudien, J. (2005). Arctic meiofauna succession. Forschungsinstitut Senckenberg; Deutsches Zentrum fur Marine Biodiversittsforschung (DZMB), Germany.
Vimoksalehi Lukoschek and B. Louise Chilvers. 2008. A robust baseline for bottlenose dolphin abundance in coastal Moreton Bay: a large carnivore living in a region of escalating anthropogenic impacts. Wildlife Research. 35(7):593-605
Vincx, M. (1984). Free-living marine nematodes from the Southern Bight of the North Sea. Ghent University, Department of Biology, Marine Biology Section (MARBIOL), Belgium.
VLIZ (2000). Tisbe, Taxonomic Information System for the Belgian coastal area. http://www.vliz.be/vmdcdata/tisbe.
Von Saunder, Alexandra, and Jay Barlow. 1999. A Report of the Oregon, California and Washington Line-Transect Experiment (Orcawale) Conducted in West Coast Waters during Summer/Fall 1996NOAA-TM-NMFS-SWFSC-264:
Von Saunder, Alexandra, and Jay Barlow. 1999. A Report of the Oregon, California and Washington Line-Transect Experiment (Orcawale) Conducted in West Coast Waters during Summer/Fall 1996NOAA-TM-NMFS-SWFSC-264:
Vriser, B. & Grego, M. (2005). Meiofauna of the Gulf of Trieste (NIB-MBS database on meiofauna version 1.2). National Institute of Biology, Marine Biological Station Piran (MBS), Slovenia
W P Goodall-Copestake1, S Prez-Espona2, M S Clark1, E J Murphy1, P J Seear1 and G A Tarling1(2010) Swarms of diversity at the gene cox1 in Antarctic krill. In press 2010
Walsh, F (1996) Northern Barrier Marine Life of the Great Barrier Reef.
Watson JE (2008) Hydroids of the BANZARE expeditions, 1929-1931: the family Haleciidae (Hydrozoa, Leptothecata) from the Australian Antarctic Territory, Memoirs of Museum Victoria 65: 165-178
Weed rinsings from CullercoatsBay, 2003 (Tim Worsfold, Unicomarine)
Weir, C.R., Stockin, K.A. and Pierce, G.J.. 2007. Spatial and temporal trends in the distribution of harbour porpoises, white-beaked dolphins and minke whales off Aberdeenshire (UK), north-western North SeaJournal of the Marine Biological Association of the UK: 87: 327-338
Welch, D. W., G. Kristianson, P. Tsang, and R. Branton An OBIS formatted summary of Pacific Ocean Shelf Tracking (POST) data. , , 2005. Retrieved from http://www.iobis.org
Wenneck, T. de Lange, Falkenhaug, T. and O.A. Bergstad. 2008. Strategies, methods, and technologies adopted on the RV G.O. Sars MAR-ECO expedition to the mid-Atlantic Ridge in 2004. Deep-sea Research II. 55: 6-28.
Weslawski J. M. (1997). Svalbard tidal zone data. Polish Academy of Sciences; Institute of Oceanology (IOPAS), Poland.
Wilkinson, S., Marine benthic dataset (version 1) commissioned by UKOOA. EUROBIS, Joint nature Conservation Committee, 07 Dec 2005, Peterborough, UK, Version: 1, DiGIR Provider, http://www.marbef.org
Wilson, NG; Schrodl, M; Halanych, KM. MOLECULAR ECOLOGY Volume: 18 Issue: 5 Pages: 965-984 Published: 2009

Wilson,N.G., Hunter,R.L., Lockhart,S.J. and Halanych,K.M. Mar. Biol. 152 (4), 895-904 (2007)
Wood J.B., Day C.L., del Pino D. Forsythe J.W., DiMarco P. Lee P.G. and ODor R.K. CephBase. http://www.cephbase.utmb.edu
Yen, P. P. W., W. J. Sydeman, K. D. Hyrenbach. 2004. Marine bird and cetacean associations with bathymetric habitats and shallow-water topographies: implications for trophic transfer and conservation. Journal of Marine Systems. 50 (2004) 79
Yen, P.P.W., W.J. Sydeman, S.J. Bograd, K.D. Hyrenbach. 2006. Spring-time distributions of migratory marine birds in the southern California Current: Oceanic eddy associations and coastal habitat hotspots over 17 years. Deep-Sea Research II. 53 (2006) 399
Yorio P., Quintana F., Lopez de Casenave J. Editors. Published by Aves Argentinas / Asociacin Ornitolgica del Plata, Buenos Aires, Argentina. 20-1: 130 pp.
Zdanowski,M.K., Weglenski,P., Golik,P., Sasin,J.M., Borsuk,P.,Zmuda,M.J. and Stankovic,A. , FEMS Microbiol. Ecol. 50 (3), 163-173 (2004)
Zeidler W. & De Broyer C., 2009. Catalogue of the Hyperiidean Amphipoda (Crustacea) of the Southern Ocean with distribution and ecological data. Bulletin de l'Institut Royal des Sciences Naturelles de Belgique 79 (Suppl. 1): 1-104.

Supplementary **Table** 7. The number of species that were used in the present analyses, and their frequency of occurrence in the 101 seas and oceans, and 2,065 marine 5° c-squares of 2,592 globally.

**Taxa**			**Occurrence in**		**Number of species in 5° squares**				
			**Seas**	**5° c-squares**	**1**	**2-10**	**11-50**	**>50**	**Total**
Annelida	Polychaeta		82	871	927	1928	982	377	4214
	Other		35	133	20	104	26	8	158
Arthropoda	Pycnogonida		51	355	212	326	44	4	586
	Crustacea	Branchiopoda	30	69	11	16	6	1	34
	Malacostraca	Amphipoda	69	754	303	1211	574	118	2206
		Cumacea	60	324	147	290	153	29	619
		Isopoda	81	745	940	1532	231	29	2732
		Mysida	59	385	104	181	58	13	356
		Euphausiacea	41	508	4	21	34	23	82
		Tanaidacea	44	365	109	280	82	11	482
		Stomatopoda	49	230	70	177	42	5	294
		Other	36	202	16	27	14	8	65
		Decapoda	85	900	1151	2690	968	176	4985
	Maxillopoda	Harpacticoida	60	308	353	533	91	18	995
		Calanoida	76	960	275	522	259	138	1194
		Siphonostomatoida	40	327	184	296	42	2	524
		Poecilostomatoida	60	403	214	632	47	11	904
		Other	69	662	199	316	65	13	593
		Ostracoda	45	376	228	451	84	41	804
	Other		32	100	59	46	7	1	113
Brachiopoda			39	262	165	89	45	6	305
Bryozoa			44	218	11	407	190	33	641
Chaetognatha			54	543	553	17	11	24	605
Chordata	Tunicata		61	553	329	551	192	29	1101
	Pisces	Actinopterygii	79	651	83	255	183	40	561
		Anguilliformes							
		Gadiformes	78	715	54	213	149	52	468
		Perciformes	88	1216	796	2654	1947	471	5868
		Pleuronectiformes	79	760	63	247	166	56	532
		Scropaeniformes	83	836	123	439	306	84	952
		Other	90	1081	413	1490	1099	267	3269
		Elasmobranchii	73	664	82	338	267	91	778
		Other	32	124	17	23	8	3	51
	Mammalia		57	798	4	17	32	50	103
	Aves		51	893	70	152	86	151	459
	Other		61	511	34	44	18	16	112
Chromista			61	551	348	468	292	218	1326
Cnidaria	Hydrozoa		76	718	293	741	276	72	1382
	Anthozoa	Scleractinia	81	725	299	793	322	52	1466
		Zoanthidea	56	266	26	61	31	6	124
		Actiniaria	83	646	182	448	230	24	884
		Alcyonacea	81	701	304	715	278	48	1345
		Antipatharia	61	380	40	92	37	9	178
		Other	63	392	53	142	60	12	267
	Scyphozoa		39	319	17	37	13	7	74
	Other		22	60	11	16	4	1	32
Ctenophora			27	96	2	8	5	2	17
Cyanobacteria			19	44	78	77	6	0	161
Echinodermata	Asteroidea		69	570	269	568	239	44	1120
	Ophiuroidea		75	626	277	597	267	51	1192
	Crinoidea		53	267	53	136	54	3	246
	Echinoidea		69	528	19	131	148	31	329
	Holothuroidea		67	529	144	246	136	22	548
Echiura			28	94	36	37	4	1	78
Fungi			13	22	25	40	11	1	77
Magnoliophyta			23	45	13	35	7	1	56
Mollusca	Bivalvia		83	666	344	829	519	148	1840
	Gastropoda		86	1036	1224	2911	1150	198	5483
	Cephalopoda		69	970	117	279	179	50	625
	Polyplacophora		45	196	81	123	35	7	246
	Other		48	203	91	151	38	9	289
Myzozoa			46	369	98	174	149	117	538
Nematoda			41	207	858	1179	276	57	2370
Nemertina			26	161	93	120	27	6	246
Other Animalia									

	Acanthocephala	2	9	23	1	0	0	24
	Acoelomorpha	3	6	10	14	0	0	24
	Cephalorhyncha	22	71	14	44	5	2	65
	Cycliophora	1	1	1	0	0	0	1
	Entoprocta	9	13	9	7	2	0	18
	Gastrotricha	7	20	29	18	0	0	47
	Gnathostomulida	1	3	2	1	0	0	3
	Hemichordata	13	45	9	13	5	0	27
	Mesozoa	4	5	4	1	0	0	5
	Phoronida	15	35	1	4	2	1	85
	Tardigrada	10	24	29	17	0	0	46
Plantae		50	186	125	281	101	29	536
Platyhelminthes		21	40	114	38	5	0	157
Porifera	Calcarea	25	52	25	36	15	3	79
	Hexactinellidea	31	214	138	124	10	1	273
	Demosphongiae	57	327	539	617	135	20	1311
Protozoa		62	799	378	475	101	52	1006
Rhodophyta		45	140	339	725	327	97	1488
Rotifera		11	22	38	13	3	0	54
Sipuncula		53	316	20	52	33	10	115
**Total**		**98**	**32517**	**15050**	**32150**	**14045**	**3811**	**65056**

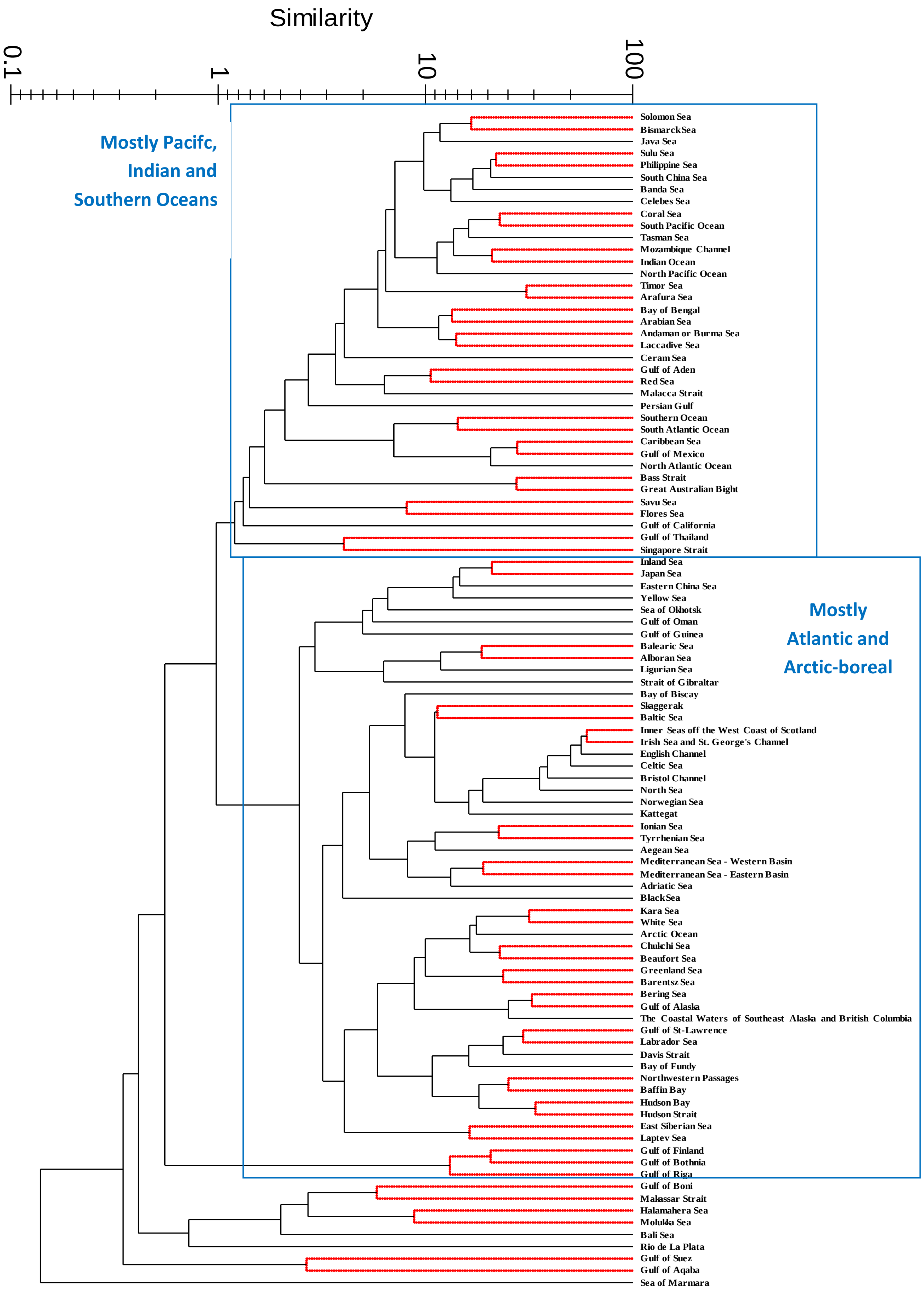

Supplementary Figure 1. Clustering of seas and oceans by their species composition (presence-only). Areas connected by red lines were not significantly different ($P < 0.05$) using SIMPROF text. Note that the similarity axis is on a log scale.

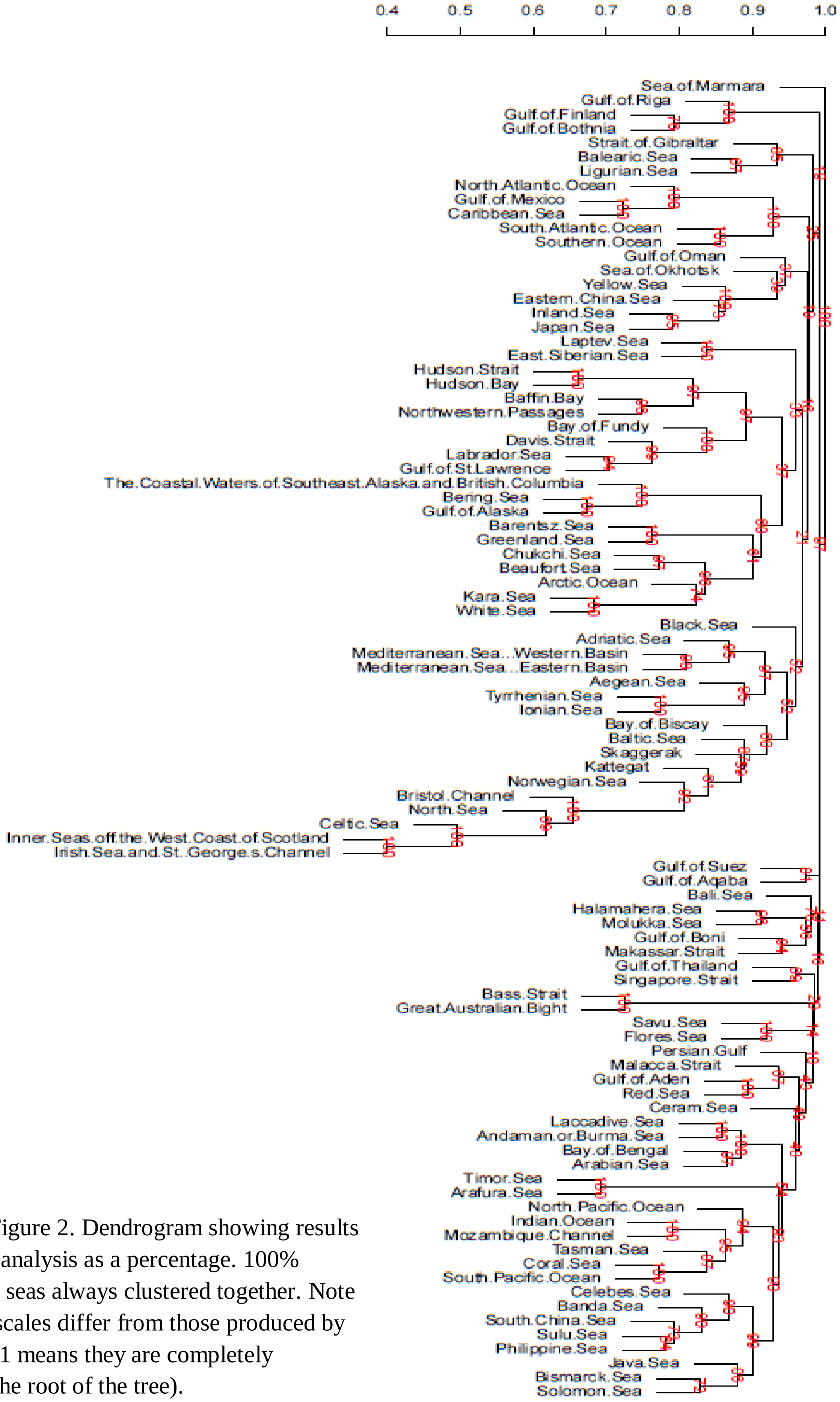

Supplementary Figure 2. Dendrogram showing results of bootstrapping analysis as a percentage. 100% indicates that the seas always clustered together. Note the dendrogram scales differ from those produced by PRIMER in that 1 means they are completely different (i.e. at the root of the tree).

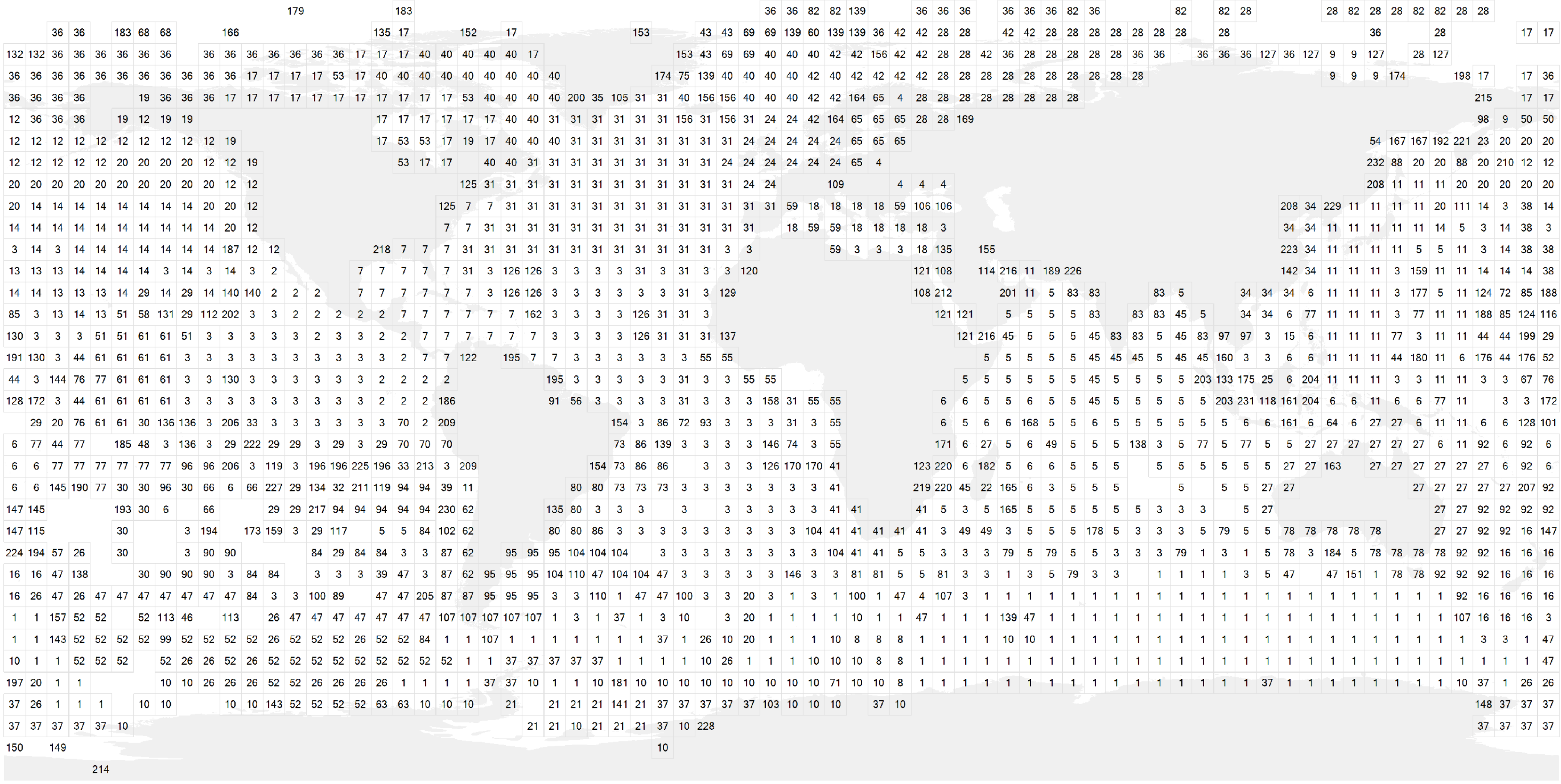

Supplementary Figure 3. Example of how the 5° areas were visualised to enable mapping of the realms. Each cell, in this case at the 6% level, was assigned a number indicating its group membership.
