# Supplementary material for: Marine biogeographic realms and species endemicity
Source: Nat Commun. 2017 Oct 20;8:1057. doi: 10.1038/s41467-017-01121-2 (PMC5648874; doi:10.1038/s41467-017-01121-2)
Supplement: Supplementary file 3 — Description of Additional Supplementary Files [file 41467_2017_1121_MOESM3_ESM.pdf]

Description of Additional Supplementary Files

File Name: Supplementary Data 1
Description: Classified list of species that distinguished the realms
